# Rhinoceros horn mineral and metal concentrations vary by sample location, depth, and color

**DOI:** 10.1038/s41598-024-64472-z

**Published:** 2024-06-14

**Authors:** Terri L. Roth, Sarah L. Rebolloso, Elizabeth M. Donelan, Louisa A. Rispoli, John P. Buchweitz

**Affiliations:** 1 Center for Conservation and Research of Endangered Wildlife (CREW), Cincinnati Zoo and Botanical Garden, 3400 Vine Street, Cincinnati, OH 45220 USA; 2 Veterinary Diagnostic Laboratory, College of Veterinary Medicine, Michigan State University, 4125 Beaumont Rd, Lansing, MI 489100 USA

**Keywords:** Physiology, Predictive markers

## Abstract

Poaching is again driving rhinos to the brink of extinction due to the demand for rhino horn products consumed for cultural, medicinal, and social purposes. Paradoxically, the same horn for which rhinos are killed may contain valuable clues about the species’ health. Analyses of horn composition could reveal such useful bioindicators while elucidating what people actually ingest when they consume horn derivatives. Our goals were to quantify minerals (including metals) in rhino horn and investigate sampling factors potentially impacting results. Horns (n = 22) obtained during necropsies of white (n = 3) and black (n = 13) zoo rhinos were sampled in several locations yielding 182 specimens for analysis. Initial data exposed environmental (soil) contamination in the horn’s exterior layer, but also confirmed that deep (≥ 1 cm), contaminant-free samples contained measurable concentrations of numerous minerals (n = 18). Of the factors examined in deep samples, color-associated mineral differences were the most profound with dark samples higher in zinc, copper, lead, and barium (*p* < 0.05). Our data demonstrate that rhino horns contain both essential and potentially toxic minerals that could be relevant to rhino health status, but low concentrations make their human health benefits or risks unlikely following consumption.

## Introduction

After recovering from near extinction once already, Africa’s white and black rhinoceroses (henceforth: rhinos) are again fighting for survival as poaching has escalated to a level that overshadows birthrates, causing numbers to decline in recent years^1^. The crisis is fueled by exorbitant profits that can be made from rhino horn which is consumed for cultural, medicinal, and social purposes^2–5^. When wild populations face extreme threats, attention often turns to maintaining robust, ex situ populations. However, ex situ environments such as those in zoos or on private ranches differ from wild habitats and often present new challenges when wild species are subjected to them.

One side-effect of ex situ management that may affect rhino health is iron overload disorder (IOD), a condition impacting both black and Sumatran rhinos (*Diceros bicornis* and *Dicerorhinus sumatrensis*, respectively) in human care. IOD was initially detected at necropsy presenting as significant iron deposits (hemosiderosis) in multiple organ tissues, especially liver and spleen^6–8^. Although rarely the cause of death^9,10^, it is ubiquitous in zoo-managed black rhinos^8,10,11^, presumed detrimental to general health, and should be minimized. Therefore, animal care staff try to monitor rhinos for iron load, but it has proven challenging. Unfortunately, IOD is insidious, and liver biopsies, the gold standard for assessing body iron loads, are too risky and difficult to perform routinely in rhinos. Serum biomarkers rely on species-specific antibodies, fluctuate independent of body iron loads, and/or sometimes produce invalid values^12–15^. An alternative method of accurately monitoring rhino iron load is highly desirable, and paradoxically, the same rhino horn targeted by poachers may provide helpful insight into IOD status and/or other health-related conditions.

Rhinos use their horns as daunting weapons during aggressive interactions with counterparts or other perceived threats, especially when protecting calves. Horns also are used in foraging behavior to break branches and for disrupting soil to form mud wallows or find water. Rhino horn is composed of keratin much like fingernails, toenails, and hair. Numerous scientific papers have already identified minerals and metals (henceforth referred to simply as minerals) that can be measured in the latter three, with most concluding that environmental concentrations of the minerals are positively correlated with those found in these keratin sources^16–20^. Of particular relevance, hair and/or nail iron concentrations are positively associated with increased body iron load caused by drinking iron-contaminated water^21^ or iron-associated medical conditions^22^. Iron concentrations are higher in nails compared to hair^21^ and represent accumulation over time which reduces the variability observed in serum samples from a single time point. Therefore, horn sampling and analysis may provide a relatively non-invasive method for monitoring body iron and other minerals in rhinos since a tiny piece of horn carved off the surface or a small amount of powder sanded from the horn’s exterior would provide enough material, and most rhinos are not averse to having their horns handled to some extent. However, there are many potential confounding factors involved in sampling and assessing rhino horn mineral content that need to be investigated prior to placing confidence in the results as a reflection of physiological status.

Rhino horn has been ingested by humans for thousands of years as one component of Traditional Chinese Medicine (TCM)^2,4^, yet just two publications from the English literature include some analyses of rhino horn mineral composition^23,24^. However, there could be papers in other languages that provide more information. As a protein, keratin consists of amino acids and is particularly cysteine-rich^25^. Cysteine attracts iron, and they work cooperatively to strengthen the keratin^26^. Because rhino horn is made of very dense keratin that arises from a vascularized matrix on the dorsal service of the rhino’s nose, it could accumulate iron accordingly, and like other forms of keratin, is likely to contain many additional minerals acquired from the circulatory system during emergence. In addition to amino acids, rhino horn reportedly contains sterols, amines, guanidine derivatives, sugar, phosphorous, and calcium^3,26^. A rhino horn CT scan produced images depicting dark patches in the center of the horn, and the authors attributed them to differences in melanin deposition likely accompanied by denser calcium concentrations^27^. In a study focused on rhino horn fingerprinting, inductively-coupled plasma mass spectrometry (ICP-MS) was reportedly used to assess mineral content as one component of the fingerprint, but the mineral data were not included^24^. Although some rhino horn mineral data were provided in a dissertation following analysis by inductively coupled plasma optical emission spectroscopy (ICP-OES)^23^, the sampling procedure itself was not well described. Several mineral values were much higher than expected in biological material, but results appeared to be based on the analysis of a single confiscated horn which made it difficult to interpret or contextualize accordingly.

In addition to the primary rationale for this work related to rhino health, detailing rhino horn elements could reveal what is truly being consumed when rhino horn is included as part of medical treatments. In addition to TCM, the recent expanded use of rhino horn for treating a variety of ailments including cancer, measles, and AIDS^4,28^ is somewhat alarming as use of a biological substance without curative properties could endanger humans requiring effective medical care^2,4^. The few reported studies on rhino horn’s medicinal properties focused primarily on antipyretic activity, and published results are contradictory with some reporting positive effects and others concluding no impact^3,29–31^. Regardless, no study has provided a logical scientific explanation regarding any rhino horn constituent’s ability to perform as an antipyretic agent in vivo after ingestion. However, other elements with potential health benefits have not been explored.

Accurately characterizing rhino horn minerals could be informative for several reasons. First, if minerals, and especially iron, can be measured reliably and reflect body loads, information from this relatively non-invasive sampling strategy may provide a window into rhino health that could facilitate ex situ management. Second, quantifying essential mineral concentrations in the horns would offer insight into potential health benefits aside from antipyretic activity when humans partake of TCM. Finally, such analysis could reveal potentially toxic compounds being ingested in horn derivatives. However, quality control in such research is important, and significant preliminary testing is required given that rhino horn is not homogeneous^27^ and sampling method/location will likely impact results. Therefore, the goals of this study were to: (1) identify minerals that can be measured in rhino horn using ICP-MS, (2) determine means and ranges in mineral concentrations found throughout the horns, and (3) detect factors that impact those concentrations such as location, color, distance from base, and anterior or posterior horn.

## Materials and methods

### Animal use statement

All biological samples utilized in this study were collected with the approval of the Cincinnati Zoo and Botanical Garden’s Institutional Animal Care and Use Committee (protocols # 20-163, 22-173, and 22-175) and the four zoological institutions from whence the biomaterials originated. All horns/horn pieces were obtained opportunistically, primarily post-mortem (or otherwise detailed below), and covered by protocol #22-175 entitled: Noninvasive and harmless opportunistic collection of biological samples from animals.

### Rhino horns

Two sets of rhino horns were acquired and sampled for the study. Group 1 Horns were all of known identity and consisted of six horns from six different rhinos of two species, white rhino (*Ceratatherium simum*; 1.0 = male.female) and black rhino (2.3). Five of these horns were whole, harvested at necropsy, and donated by one facility. The sixth was a male black rhino horn piece that had broken off of a living animal and was donated by another facility. Group 2 Horns consisted of 16 horns, 15 of which were whole, harvested at necropsy, and donated from the same facility but without individual identification information. Based on subsequent rhino horn DNA analysis (see below), it was determined that these horns originated from 9 different rhinos. One additional rhino horn piece was acquired from a second facility, so in total, Group 2 Horns represented 10 rhinos of two species, white rhino (0.2) and black rhino (5.3). All whole horns in both groups came from rhinos that had been living in Florida facilities antemortem, whereas the two horn pieces came from rhinos living in Ohio facilities.

Exam gloves were worn whenever handling the horns. To remove surface environmental contaminants (e.g., soil), all horns were scrubbed with mild dishwashing soap and warm tap water, rinsed thoroughly, and allowed to dry for at least 24 h before sampling. Because many horns were harvested during necropsies, a layer of dried skin often covered the base. It was removed when possible or simply avoided during sampling if complete removal failed.

#### Group 1 horn sampling

**Figure 1:**
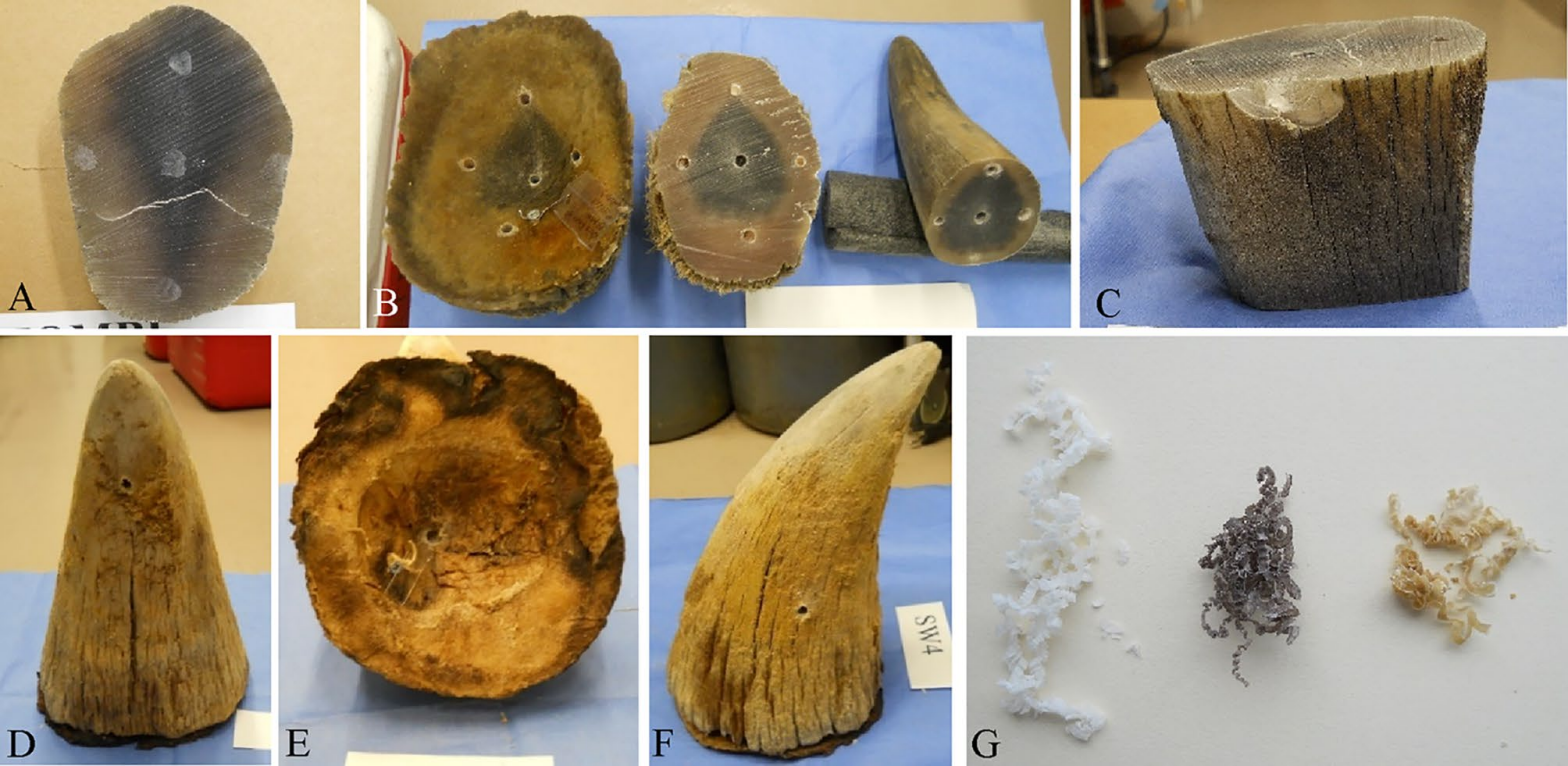
Examples of horn sampling locations and methods for Group 1 Horns (**A**–**C**) and Group 2 Horns (**D**–**F**). A. Horn bottom after cutting 8 cm from the base and sanding sample sites with Dremel prior to drilling. **B** Core sampling in center, dorsal, ventral and lateral positions different distances from base after cutting horn twice to produce samples at 0 cm, 5–15 cm and > 15 cm from base. **C** Example of a horn surface sample collected by slicing a thin surface horn sliver with a Dremel diamond blade. **D**–**F** display dorsal (20 cm from base), center (at base) and lateral (10 cm from base) samples drilled from Group 2 Horns. **G** Example of rhino horn coil colors (white, gray, rust).

At each sample site of Group 1 Horns, the outer surface of the horn was removed by sanding the area lightly with a diamond-covered wheel or burr bit on a Dremel tool (Fig. 1A). A 1/4″ titanium-coated drill bit on a variable speed drill set to its slowest speed produced the desired horn coils. The drill bit was cleaned, soaked in ethanol, rinsed in distilled water and dried between each new sample location. Horn coils were transferred with forceps into plastic tubes for storage. Each of the five whole horns were sampled from the base in the center, dorsal, ventral, right lateral and left lateral positions (Fig. 1B). If dried dermis was still attached to the base of the horn, sampling positions shifted slightly to avoid the dermis, or the hole was drilled through the dermis before coil collection commenced. After initial base sampling, horns were cut transversely with a band saw once or twice depending on their length and sampled similarly from the freshly cut surfaces at varied distances from the base (Fig. 1B). The cuts closest to the horn tip yielded a small base and sometimes just one center sample was taken. In total, 11–16 drilled samples were collected per horn. The one exception was the black rhino horn piece which was both short and narrow and was only sampled once from the existing base in the center, dorsal and ventral positions (n = 3). Additionally, samples were taken from the surface of each horn (n = 1–4 surface samples/horn) by sanding and collecting the powder on chemical weigh paper or by cutting a thin slice from the exterior surface of the horn with the Dremel diamond wheel tool (Fig. 1C).

#### Group 2 horn sampling

Group 2 Horns were given a numbered identification tag and sampled using the same drilling method described above with one modification. Horn coils collected as the drill bit progressed from the surface to 1 cm in depth “shallow” were placed in a plastic tube labeled “Sample 1”, the horn was flipped over and tapped several times to make sure all residual drilled horn came out of the hole, the drill bit was wiped clean with ethanol and rinsed in distilled water, and then drilling continued until the bit reached approximately 2 cm into the horn. All horn coils collected 1–2 cm “deep” were placed into another tube labeled “Sample 2”. Samples were collected at 2–5 locations from each horn, with a minimum of one center base sample and one surface sample per horn (Fig. 1D–F).

### Rhino horn DNA analysis

#### Samples and genomic DNA extraction

Reference samples were comprised of tissue, sperm, and horn derived from deceased and living black (n = 7), white (n = 3), and greater one-horned (GOH; n = 2) rhinos of known identification. Genomic DNA was extracted at CREW from the known and unknown samples using the GeneJet Genomic DNA purification kit (Thermo Fisher) as per the manufacturer’s instructions with modifications depending on the sample type (details in the Supplementary Material). During each extraction procedure, an extraction control was included which entailed a tube without any sample being handled and exposed to the same buffers and conditions as the samples undergoing extraction.

#### Sex identification assay

A multiplex PCR assay was utilized to determine the sex of the rhino from whence an unknown horn sample was derived. Each reaction contained oligonucleotides to amplify proteolipid protein 1 (PLP1; Supplementary Table S1) and sex-determining region Y (SRY; Supplementary Table S1). Details about oligonucleotide design can be found in the Supplementary Material. Extracted gDNA was assessed using experiment type presence/absence on an Applied Biosystems (ABI) QuantStudio 3 instrument with the PLP1 designated as the internal positive control gene (IPC; i.e., the gene present in all samples) and SRY designated as the target gene (i.e., could be present or absent). Details for the reaction and thermal cycling conditions can be found in the Supplementary Material. For the non-template control (NTC), Tris–EDTA (TE) buffer (10 mM Tris–HCl, 0.1 mM EDTA, pH 8.0) was substituted for gDNA. For negative control wells (i.e., IPC present and no template control for SRY), gDNA derived from known female tissue (mixed species) was utilized, whereas the positive control wells contained gDNA from known male rhinos (mixed species). The QuantStudio Design and Analysis Software (v1.5.1) automatically compared the pre- and post-read fluorescent values to determine the presence (male) or absence (female) of the target gene using the NTC and negative control wells to set fluorescent thresholds. Any sample without amplification of IPC was deemed undetermined. Reference horn samples were assessed by an individual blind to their origin to validate the assay; nine of nine samples were correctly identified.

#### Species identification assay

A modified version of the multiplex PCR assay described by Ewart et al.^32^ was utilized to determine the species of unknown horn samples. Specifically, species-specific primers for cytochrome b (Supplementary Table S2^32^) were combined and the resulting amplicon was visually assessed for size on an agarose gel. Amplicon length differed by species, black rhino ≈ 222 bp, white rhino ≈ 266 bp, and GOH ≈ 310 bp. Each assay run included a NTC with TE buffer substituted for the gDNA template. Other controls included the extraction control, and gDNA derived from known black, white, and GOH rhino samples. An individual blind to their origin assessed reference horn samples to validate the assay; nine of nine samples were correctly identified.

#### Individual identification assay

Twenty-three microsatellite loci were evaluated on horn samples of unknown origin using a modification of the procedure described by Harper et al.^33^ Direct labeling of primers was omitted for this assay, instead locus-specific forward primers were amended to include the sequence for one of four universal primer tags on the 5′ end (Supplementary Table S3) as described by Blacket et al.^34^ A 2-round multiplex PCR approach was utilized with the first round including locus-specific primers only, and the second run occurring after the addition of universal primers labelled with fluorescent dyes to the reaction mix. Each multiplex reaction was subjected to capillary electrophoresis on an ABI 3500 series Genetic Analyzer located in the DNA lab within the Cincinnati Museum Center. Known black, white, and GOH rhino control samples were included with each sample run to ensure the accuracy of the allele calls between runs. The resulting data were analyzed for peaks for each locus within a multiplex panel. Profiles for each horn sample were compared and those with matches across all four panels were determined to originate from the same individual. Detailed descriptions for the reactions, instrument settings, and peak analyses can be found in the Supplementary Material.

### Mineral analysis

Rhino horn samples (~ 100 μg/sample) were sent to Michigan State University for processing and mineral analysis. Samples from different horns and sampling locations were randomly assigned a simple numerical ID so the staff performing ICP-MS were blind to any detailed information about the samples. Group 1 Horn samples were examined for 22 minerals including aluminum (Al), antimony (Sb), arsenic (As), barium (Ba), cadmium (Cd), calcium (Ca), chromium (Cr), cobalt (Co), copper (Cu), iron (Fe), lead (Pb), magnesium (Mg), manganese (Mn), mercury (Hg), molybdenum (Mo), phosphorous (P), potassium (K), selenium (Se), sodium (Na), sulfur (S), thallium (TI), and zinc (Zn), whereas Group 2 Horn samples were examined for 12 minerals after dropping two that were not detected in the first analysis (Mn, TI) and excluding those that were unlikely to be associated with rhino health conditions due to toxicity or deficiency and/or were not useful as indicators of environmental contamination (S, Ca, P, Na, K, Mg, Mo, Sb).

#### Sample preparation

Rhino horn samples were weighed and digested with 2 mL concentrated 67–70% nitric acid (Aristar Plus, VWR, Radnor, PA, USA) at 95 °C overnight in 15 mL polypropylene digestion vessels. The digests were then diluted 1:100 in deionized water (ELGA Purelab Flex, Woodridge, IL, USA) prior to analysis.

The limits of quantitation for the elements of interest are as follows: Al 0.20 μg/g, As 0.005 μg/g, Ba 0.25 μg/g, Fe 0.20 μg/g, Cd 0.1 μg/g, Co 0.002 μg/g, Cr 0.05 μg/g, Cu 0.08 μg/g, Hg 0.5 μg/g, Mn 1.00 μg/g, Mo 0.02 μg/g, Pb 0.025 μg/g, Tl 0.1 μg/g, Se 0.02 μg/g, and Zn 0.2 μg/g.

#### ICP-MS mineral analysis

Sample digests were analyzed by ICP-MS (Agilent 7900 ICP-MS, Agilent Technologies, Santa Clara, CA, USA.). Elemental concentrations were calibrated using a 6-point linear curve of the analyte-internal standard response ratio. Calibration standards were from Inorganic Ventures (Christiansburg, VA, USA.) The National Institute of Standards and Technology (NIST, Gaithersburg, MA, USA) Standard Reference Materials (Bovine Liver 1577c and Mussel 2976) were used as controls. A second source calibration verification (High Purity Standards, North Charleston, SC, USA) was also used for quality control on the ICP-MS.

### Horn mineral comparisons between sample types

#### Surface and core samples (Group 1 Horns)

Mineral content of core horn samples obtained by drilling horn coils from the base of the horn was compared to that of surface horn samples collected in powder form by sanding the exterior surface of the horn or in thin slices cut along the surface of the horn. Surface sampling as described could be feasible from living rhinos that are not anesthetized, whereas drilling into the horn would likely require sedation or anesthesia, and base sampling could only be conducted post-mortem unless a living rhino knocked its horn off. Therefore, it was important to determine if results from all sample locations would be similar. A total of 88 samples contributed to this comparison. All six horns contributed both core (n = 74) and surface (n = 14) samples and 19 different minerals were evaluated.

#### Shallow and deep drilled samples (Group 2 Horns)

Group 2 Horn samples consisted of paired tubes of horn coils drilled from the same locations but collected at different depths, shallow (0–1 cm) and deep (1–2 cm), to determine if environmental contamination could be avoided by consistently sampling deeper into the horn regardless of where on the horn the sample was obtained. Therefore, these paired samples were first compared across all sampling locations to determine if a 1–2 cm drill depth was sufficient to avoid any environmental contamination in the mineral analysis. Twelve minerals were analyzed in a total of 94 samples originating from all 16 horns and 10 rhinos in this comparison, n = 47 shallow samples and the matched n = 47 deep samples.

#### Deep-drilled surface and base samples (Group 2 Horns)

Mineral concentrations in deep samples collected by drilling into the surface of the horn were compared to those in deep samples collected by drilling into the base of the horn. A total of 47 samples from 16 horns and all 10 rhinos were included in the analysis and were split between the two groups: base (n = 24) and surface (n = 23). Every horn contributed at least one base and one surface sample to the analysis.

#### Anterior and posterior horns (Group 2 Horns)

Because DNA analyses revealed that there were six matched sets of horns, mineral content of the anterior and posterior horns was compared to determine if there were significant differences between the two horns on the same rhino. Using only deep samples (n = 35) from those 12 horns, yielded 15 posterior and 20 anterior samples for comparison.

### Combined horn sample analyses

For the final three analyses to determine if there is an impact of sampling position (relative to the center of the horn), sample color, and distance from the base on mineral content, samples from Group 1 and 2 Horns were combined. Only samples considered free of environmental contamination based on the mineral results were analyzed which encompassed core samples from Group 1 Horns (n = 74) and all deep samples from Group 2 Horns (n = 47) for a total of 121 samples from 22 horns of 16 rhinos, 3 white rhinos (1.2) and 13 black rhinos (5.8).

#### Horn sampling position

To determine if the mineral composition of rhino horn differs depending on sampling orientation relative to the horn’s center core, deep samples drilled from the horn’s base but dorsal, ventral, and lateral (right and left) to the center, or from the surface in lateral, ventral or dorsal locations on the horn were compared for differences based on orientation within the horn (Fig. 1B). All 121 samples were included in the analysis: center (n = 34), dorsal (n = 21), ventral (n = 21) and lateral (n = 45).

#### Horn sample color

Rhino horn coil samples ranged in color from dark gray to white. Sample color was recorded at the time of collection and often consisted of more than one color (i.e., white/gold, gray/rust) or was accompanied by an adjective describing degree of color (i.e., dark gray, light rust). To analyze the impact of sample color on mineral content, horn samples had to be categorized into distinct colors. Therefore, if two colors were indicated, the sample category assigned was that of the first one since it would coincide with the dominant color. Additionally, adjectives describing degree of color were dropped so that samples listed as dark gray and light gray would simply fall into the gray category. Following this grouping strategy, there were only five samples in the “gold” category, so they were dropped from the analysis. A total of 116 samples were analyzed in three color categories; gray (n = 44), white (n = 49) and rust (n = 23) (Fig. 1G). Finally, because the classification of “rust” and “gray” was subjective and somewhat challenging, a final analysis of color groups was performed to simply compare light to dark horn samples with white and gold samples in the light category and rust and gray assigned to the dark category. All 121 deep samples were used for this final analysis: light (n = 54), and dark (n = 67).

#### Distance from base

In addition to sampling from the base of every whole horn, samples were taken at variable distances 5–30 cm up from the base of the horn that would represent different months/years of the rhino’s life. To determine if there was any significant shift in mineral content as samples were taken further from the base and closer to the tip of the horn, samples were divided into one of three groups based on their location measured from the base: A = at base or 0 cm from base (n = 51), B = 5–15 cm from base (n = 43), and C =  > 15 cm from base (n = 27). All 121 deep samples in the combined pool were included in the analysis.

### Statistical analyses

All data were analyzed using the statistical programming language R ver. 4.2.2^35^. Because there were several samples from horns of each rhino, linear mixed-effects regression^36^ was performed for each mineral after log-transformation if necessary to meet the assumption of normality. Post-hoc testing was performed for analyses with three or more categories using estimated marginal means^37^ and the Kenward–Roger degrees-of-freedom approximation method, with a Tukey *p* value adjustment method. The comparison of mineral concentrations in samples collected 0–1 cm compared to those collected 1–2 cm deep in the Group 2 Horns was conducted using a paired t-test. A *p* value < 0.05 was considered significant for all statistical tests.

## Results

### Group 1 horns

#### Surface and core horn sample comparison

It was readily apparent from the raw data that Group 1 Horn surface and core samples differed in their mineral content (Table 1). A total of 16 minerals differed between the two sample types, 4 were higher in core samples and 12 were higher in surface samples. Only 3 of the 19 measurable minerals did not differ (*p* > 0.05) between the two sample types (P, Se, Mo). In particular, Fe, Al, and Ba were approximately 10–100 times higher in surface samples compared to drilled core samples indicating the presence of environmental contamination.

**Table 1:** Mineral concentrations (means and ranges; μg/g) that differed between rhino horn samples from Group 1 Horns collected by drilling coils from the core (n = 74 samples from 6 rhinos) and those collected by sanding/slicing horn from the surface (n = 14 samples from 6 rhinos). *P* values from linear mixed effects models.

Statistical result	Mineral	Surface mean (range) μg/g	Core mean (range) μg/g	*p* Value
Core > surface	S	18,379 (12,639–30,122)	23,051 (14,911–32,372	0.008
	Na	403.9 (125.6–630.5)	637.4 287.2–1,316.7)	0.0004
	K	226.2 (80.0–352.0)	355.7 (169.0–770.0)	0.0022
	Zn	98.59 (55.97–158.5)	117.48 56.5–216.62)	0.018
Surface > core	Ca	2,132 (812–3,509)	1,713 (705–3,594)	0.0258
	Fe	283.07 (0.196–862.4)	9.163 (0.186–82.6)	0.0001
	Mg	275.98 (73.27–525.68)	140.85 (67.83–193.49)	0.0001
	Al	156.76 (0.20–527.95)	1.522 (0.20–39.68)	0.0001
	Cu	10.549 (2.494–75.77)	2.661 1.239–7.766)	0.0005
	Mn	4.03 (1.0–8.36)	< 1.0	0.0001
	Ba	2.376 (0.25–7.237)	0.2708 (0.247—0.816)	0.0001
	Cr	1.7151 (0.164–11.635)	0.2546 (0.113–0.726)	0.0001
	As	1.1458 (0.0374–11.0569)	0.1835 (0.0127–0.8139)	0.0014
	Pb	0.399 (0.10–1.81)	0.1023 (0.10–0.2595)	0.0001
	Co	0.3112 (0.0048–2.0203)	0.01628 (0.002–0.1021)	0.0001
	Sb	0.1016 (0.0245–0.8890)	0.0268 (0.0247–0.1208)	0.0034

#### Mineral characterization

Of the 22 minerals examined in the Group 1 Horn core samples, 4 were not present in detectable concentrations (Mn, Hg, Ca and Tl). The remaining 18 measured minerals are listed in order from highest to lowest mean concentrations in Table 2. Two of these minerals (Sb and Co), though detectable, were consistently present in negligible concentrations (< 0.125 μg/g). Of the 18 measured minerals, 12 are essential for human health and typically included in daily multivitamin/multimineral supplements. Table 3 displays the quantity of essential minerals based on our results that would be present in a typical TCM daily dose of rhino horn (50 mg/kg; 80 kg person; Laburn and Mitchell^30^) compared to daily dietary reference intakes^38^ (DRI) and the amount found in a typical adult daily multivitamin/multimineral supplement^38^.

**Table 2:** List of 22 minerals that were analyzed in rhino horn core samples (n = 74) from Group 1 Horns ranked in order from highest to lowest mean concentration. Concentrations of Mn, Hg, Cd and Tl always fell below detectable levels, whereas Sb and Co were present but in negligible concentrations.

Rank	Mineral	Mean (μg/g)	Range (μg/g)
1	S	23,051	14,911–32,372
2	Ca	1713	705–3594
3	P	985.9	255.0–2079.0
4	Na	637.4	287.2–1316.7
5	K	355.7	169.0–770.0
6	Mg	140.9	67.8–193.5
7	Zn	117.5	56.5–216.6
8	Fe	9.163	0.186–82.599
9	Cu	2.661	1.239–7.766
10	Al	1.522	0.200–39.683
11	Se	0.5385	0.2569–0.8082
12	Mo	0.3236	0.0836–1.2537
13	Ba	0.2708	0.2470–0.8160
14	Cr	0.2456	0.1130–0.3025
15	As	0.1835	0.0127–0.8139
16	Pb	0.1023	0.1000–0.2595
17	Sb	0.0268	0.0247–0.1208
18	Co	0.0163	0.0020–0.1021
19	Mn	< 1.0	NA
20	Hg	< 0.5	NA
21	Cd	< 0.1	NA
22	Tl	< 0.1	NA

**Table 3:** Concentrations of essential minerals found in rhinoceros horn compared to the dose typically found in an adult daily multivitamin/multimineral supplement* and dietary reference intakes^38^.

Essential minerals	Rhino horn (in 4 g)	Daily supplement (adult)	Dietary reference intakes
Ca	6.852 mg	200 mg	1000 mg
P	3.944 mg	20 mg	700 mg
Na	2.548 mg	0.0 mg	1500 mg
K	1.424 mg	80 mg	2600–3400 mg
Mg	0.564 mg	50 mg	320–420 mg
Zn	0.468 mg	11 mg	8–11 mg
Fe	0.0368 mg	18 mg	8–18 mg
Cu	10.644 μg	500 μg	900 μg
Se	2.154 μg	55 μg	55 μg
Mo	1.294 μg	45 μg	45 μg
Cr	0.9824 μg	35 μg	25–35 μg
Mn	< 1.0 μg	2.3 mg	1.8–2.3 mg

*Product information: Equate™ complete multivitamin/multimineral supplement for adults.

### Group 2 horns

#### Mineral characterization

Of the 12 minerals analyzed in Group 2 Horn samples collected 1–2 cm deep, only Hg and Cd were below detectable levels in all samples. The remaining 10 were present in concentrations similar to those reported for Group 1 Horn samples except for Pb which was lower (*p* < 0.001) in the Group 2 Horns (Supplementary Table S4).

#### Shallow and deep sample mineral comparisons

When matched shallow (0–1 cm) and deep (1–2 cm) samples were compared for mineral concentrations, only three minerals were significantly different: Fe, As, and Co (Table 4). Fe was higher in the shallow samples, whereas As and Co were higher in the deep samples. Cu also trended higher in the deep samples but did not quite reach significance (*p* = 0.052). Because depth of sampling slightly impacted these few mineral concentrations, potentially due to lingering environmental contamination near the horn’s surface, only deep samples were used for further comparisons to avoid the confounding impacts of depth/contamination.

**Table 4:** Mineral concentrations (means ± SEM) that differed in a paired t-test analysis comparing rhino horn samples from Group 2 Horns (n = 16 horns; n = 10 rhinos) collected by drilling shallow (0–1 cm; n = 47) versus deep (1–2 cm; n = 47) in each sampling location.

Mineral	Shallow (μg/g)	Deep (μg/g)	*p* Value
Fe	8.292 ± 0.661	6.529 ± 0.501	0.014
Cu	2.202 ± 0.122	2.294 ± 0.123	0.052
As	0.088 ± 0.007	0.112 ± 0.007	< 0.001
Co	0.018 ± 0.005	0.034 ± 0.012	0.045

#### Surface and base sample mineral comparisons

Mineral concentrations in deep samples drilled from the horn’s surface and base were similar except for Se which was higher (*p* = 0.013) in base samples. Cu also trended higher in base samples but was not statistically different (*p* = 0.054).

#### Anterior and posterior horn comparison

Mineral concentrations in deep samples from both anterior and posterior horns were similar with the exception of Cu which was higher (*p* = 0.047) in the posterior horn. Al trended higher in the anterior horn but did not quite reach significance (*p* = 0.051). Fe exhibited the strongest positive correlation (*p* = 0.93) between the two horns.

### Combined horn samples

#### Sampling position relative to center core

Sampling position was associated with differences in Cu, Zn, and Ba concentrations. Cu was higher in center samples than in dorsal (*p* = 0.013), lateral (*p* < 0.001), and ventral (*p* < 0.001) samples. Zn was higher in center samples compared to lateral (*p* < 0.001) and ventral (*p* = 0.0405) samples but not different than dorsal samples that also contained more Zn than lateral samples (*p* = 0.003). Finally, Ba was higher in center samples compared to lateral samples (*p* = 0.024).

#### Sample color

**Figure 2:**
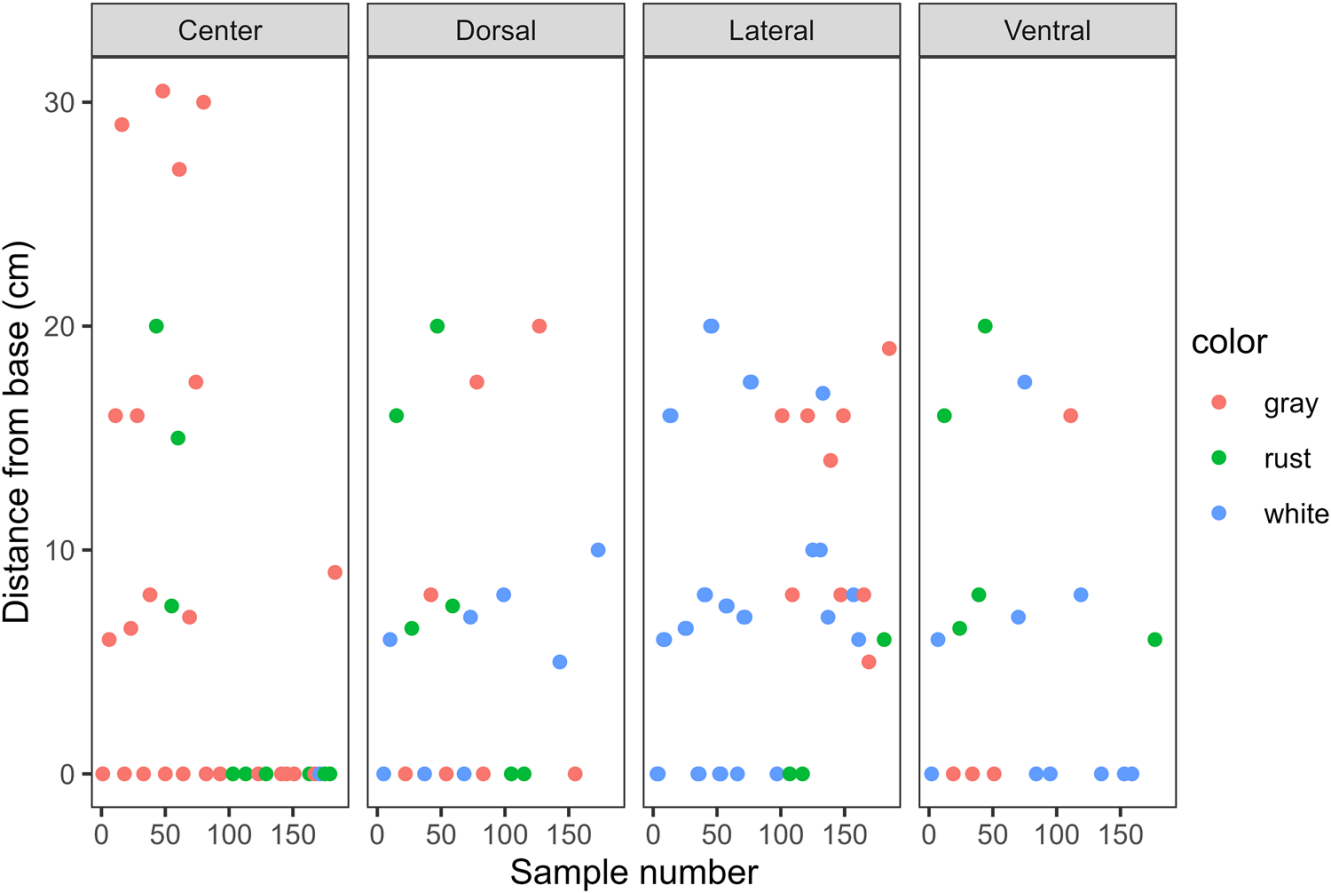
Scatter plots depicting the color of the rhino horn samples collected at different distances from the horn base, and sampled from the center, dorsal, lateral, or ventral positions.

Sample color was correlated with differences in four minerals: Cu, Zn, Pb, and Ba (Table 5). However, copper and zinc were the primary minerals associated with differences in sample color with Ba only different between gray and white samples (*p* = 0.009) by 0.039 μg/g and Pb differing between gray and white samples (*p* = 0.042) by 0.020 μg/g. Both Cu and Zn were substantially higher in the gray and rust samples (*p* < 0.001) compared to the white samples. Results of the dark versus light colored sample analysis were clear with all four of the above minerals significantly lower in light versus dark samples (*p* < 0.001 for Cu and Zn, *p* = 0.041 for Pb, *p* = 0.0164 for Ba). Because sample color and position appeared to be confounded, samples were graphed based on sampling distance from the base, orientation to the center, and sample color to visually demonstrate that lateral samples were more likely to be white, whereas center core samples were dark, and samples near the horn tip also were predominantly dark (Fig. 2).

**Table 5:** Mineral concentrations that differed between rhino horn samples of different colors. Results of the Tukey post-hoc following linear mixed-effects modeling (estimated difference, standard error, degrees of freedom and *p* value) are shown.

Mineral	Contrast	Estimate (μg/g)	SE	df	*p* Value
Cu	Gray–Rust	0.0807	0.182	103	0.8978
	Gray–White	0.8378	0.145	102	< 0.0001
	Rust–White	0.7572	0.181	103	0.0002
Zn	Gray–Rust	8.05	6.46	106	0.4288
	Gray–White	37.46	5.16	104	< 0.0001
	Rust–White	29.40	6.42	105	< 0.0001
Pb	Gray–Rust	0.0132	0.0103	107	0.4099
	Gray–White	0.0202	0.0083	105	0.0417
	Rust–White	0.0070	0.0103	106	0.7735
Ba	Gray–Rust	0.0352	0.0159	113	0.0742
	Gray–White	0.0389	0.0129	113	0.0088
	Rust–White	0.0037	0.0159	112	0.9714

#### Distance from base

In the original linear mixed effects analysis, several minerals differed significantly with distance from the base of the horn including Fe, Cu, As, Se, Hg, and Cr. However, the number of statistically significant Tukey post-hoc comparisons was reduced to just a couple. As was greater (*p* = 0.005) at the base compared to 5–15 or > 15 cm from the base, and Se was greater in samples from 0 to 15 cm from the base compared to samples collected > 15 cm from the base. In general, the differences were somewhat variable with no consistent pattern of mineral increase or decrease as sampling moved from the base up toward the tip of the horn.

## Discussion

This study is the first systematic assessment of rhino horn mineral concentrations. Intrinsic to the study were efforts to reveal sampling factors potentially impacting mineral values due to the heterogeneous nature of the horns. We discovered significant environmental contamination in the exterior layer of the horn but also succeeded in identifying numerous minerals consistently measurable in samples from deep within the horn that would have originated from the rhino as the horn keratin emerged. Results confirmed that several mineral concentrations differ depending on sample color and that a notable range in mineral concentrations exists among samples.

Morphological variation among rhino horns received for this study was substantial with some smooth and hard from base to tip, others cracked or splitting, and still others riddled with thin longitudinal creases around the base of the horn (Fig. 1D, F). In some cases, sections of the horn’s surface were adorned by an obvious layer of hard, bristly hair near the horn’s base. However, the center core of every horn base appeared dark compared to the perimeter (Fig. 1), and the lighter perimeter layer thinned from base to tip so that the end of long horns was completely dark. This dark core has previously been described following a CT scan of a white rhino’s horn^27^.

Of the 22 minerals targeted in the ICP-MS analysis, 18 were consistently within the limits of detection and four fell below those limits as shown in Table 2. However, there was a broad range in most mineral concentrations suggesting significant differences among samples and/or rhinos. In particular, surface samples from Group 1 Horns contained much higher concentrations of Fe (> 30x), Al (> 100x), Cu (~ 4x), Ba (> 8x) Cr (> 6x), As (> 6x), Pb (> 3x), Co (> 19x), and Sb (~ 4x) when compared to core samples. Because some of these mineral values far exceed concentrations typical for biological samples and better reflect those in soil^39^ we concluded that these surface samples were contaminated despite the cleansing process and light horn surface sanding prior to sampling. Given both the creased appearance of some horns, especially at the base where the new horn is emerging, and the resulting mineral variances, it became evident that rhinos incorporate soil from the environment/mud wallows into their somewhat porous external horn surface as it emerges, and that environmental contamination is not just superficial. This finding could explain the high values of Al, Fe and Hg reported by Appiah^23^ if the rhino horn samples in that study were not taken deep enough to avoid all environmental contamination. Fortunately, by drilling 1 cm into the horn we could avoid such contamination and collect samples that contained only mineral contributions originating from the rhino. In fact, just drilling into the horn versus sanding or chipping a sample from a cleaned and lightly sanded horn's exterior surface reduced environmental contamination significantly. However, iron was still higher in the shallow samples and Cu also trended higher suggesting some environmental contamination may still be impacting results in the first 1 cm of rhino horn (Table 4). Therefore, our subsequent analyses only included deep (1–2 cm) samples and those taken from the horn’s base far from the perimeter. Based on these findings, sampling horns on live rhinos for accurate mineral assessment will likely be difficult without sedation or anesthesia since drilling into the horn proved essential.

Although several potentially toxic minerals (Al, Cu, Ba, As, Pb, Co, Sb) were detected in most horn samples, concentrations generally were low and would not exceed allowable upper limits^40^ or pose significant human health risks if ingested at the typical 4–5 g daily dose prescribed to TCM patients^30^. However, it is important to note that surface samples with soil contamination did contain significantly higher concentrations of all toxic minerals (Table 1). Because there is no quality control testing of rhino horn products, samples would vary significantly in mineral concentrations with some potentially reflecting levels found in the rhino’s environment, especially the soil, rather than the rhino itself. In contrast, rhino horn also contained 12 essential minerals found in daily vitamin/mineral supplements (Table 3). Yet, the quantity of these essential minerals consumed in a typical TCM daily dose of rhino horn would be substantially lower than the DRIs^38^ and/or concentrations found in vitamin/mineral supplements. Therefore, it seems implausible that any health benefits via mineral supplementation from rhino horn exist. Had we found rhino horn high in Fe, one could consider the possibility that anemic, lethargic individuals would feel more energetic following a dose of rhino horn assuming the Fe was in a bioavailable form, but that is simply not the case as rhino horn’s Fe concentration is almost 500× lower than that provided in a daily vitamin supplement and not even close to the 8–18 mg DRI^38^.

Although far too low for any beneficial impact on human health, Fe concentrations in rhino horn were relatively high compared to other trace minerals examined with only Zn present in greater concentrations. Furthermore, the range in Fe concentrations far exceeded the range for any other trace mineral measured, suggesting significant variation between rhinos. Together, these data support the notion that horn Fe may vary depending on body load. Furthermore, since many minerals can be measured in rhino horn, it may provide biomarkers of health beyond IOD. The associations between human hair or nail biomarkers and disease have been studied for years^41,42^. For example, interesting relationships between human nail Se, furosine, and glycated proteins and type 2 diabetes have been reported^41^ and may likewise be of interest in rhinos since ex situ conditions appear to contribute to decreased insulin sensitivity^43^.

Sampling location comparisons were performed after excluding all samples potentially impacted by environmental contamination. Rhino horn can grow fairly rapidly (approximately 3–9 cm/year), and growth is affected by the individual’s age and sex^44,45^. Because rhino diets/environments may change throughout the year, it is possible that such changes are reflected by differences in horn mineral content depending on where the horn is sampled between the base and tip. We detected only a few significant differences in minerals as the sampling location moved from horn base to tip, but they were variable with no consistent pattern that might indicate a confounding factor involved. These differences were likely random or reflected small changes in rhino body mineral concentrations over time as expected with changing environmental/dietary conditions.

The heterogenous composition of the rhino horn could also impact mineral composition, but when central core samples from the base were compared to those collected dorsally, ventrally and laterally, very few differences were revealed among the 12 minerals analyzed in Group 2 horns. Those minerals that did differ between these sampling locations most likely did so because of the propensity for samples from certain locations to be light or dark in coloration. Core samples were invariably classified as rust or gray-colored (dark), whereas samples collected laterally were most often white (light) (Fig. 2). Our analysis of sample color clearly delineated these color-associated mineral differences. The simple comparison of light (white, gold) and dark (gray, rust) colored samples indicated that Cu, Zn, Pb, and Ba were present in higher concentrations in the dark samples. However, categorizing color into white, rust and gray helped to demonstrate that the darkest samples (gray) contained the highest concentration of all four minerals, and the rust samples contained more Cu and Zn than the white samples with no difference in Pb and Ba (Table 5). Hieronymus et al.^27^ suggested that the dark patches of the rhino horn core observed in the CT scan were due to increased melanin and Ca salts. Ca was not among the 12 minerals analyzed in Group 2 Horns, but for Group 1 Horns, it was almost twice as high in dark center core samples (2420 mg/g) compared to light lateral samples (1293 mg/g) supporting the assumptions of the 2006 publication. However, this study revealed that additional minerals beyond Ca also differ in the dark core versus light lateral samples of the horn.

Despite the mineral differences noted above, neither sampling location nor sample color impacted iron concentrations if samples were taken ≥ 1 cm from the horn’s surface to avoid environmental contamination. Furthermore, the mineral content of matched posterior and anterior horns was quite similar, especially with regard to Fe. Although Al and Cu trended differently, these variances may be random and could disappear when a larger number of horn pairs are analyzed. Therefore, anterior and posterior horn samples should provide comparable mineral composition data for individual rhinos.

Although a large number of rhino horns were sampled for this study and an extensive variety of sampling locations/methods were tested, there were some weaknesses worth noting. All horns came from zoo-maintained rhinos rather than wild rhinos, all zoo rhinos were from just four North American facilities, and a large majority (20/22) came from one of two states. Horns from rhinos living in different climates/states and on other continents could differ more in their mineral composition due to the extent of environmental and dietary variation associated with different regions of the world. Additionally, species and sex could influence horn mineral content, but there were too few white rhino horns to make those statistical comparisons. Finally, we did include samples from six matched sets of horns. Although we carefully controlled for rhino in all statistical analyses, it is still unlikely our results reflect the full extent of variation that exists in rhino horn mineral composition.

In conclusion, rhino horn contains numerous minerals, both essential and potentially toxic, that could serve as valuable bioindicators of rhino health following validation that they reflect systemic and/or tissue concentrations. However, all minerals are in such low concentrations that their benefits or risks to human health following ingestion of rhino horn products would be inconsequential. One exception could be samples harvested from the outer layer of the rhino horn due to substantial environmental contamination that results in much higher concentrations of some minerals commonly found in soil. When horns are sampled deeply, soil contamination can be avoided, but there is still some variation in mineral content depending on the color of the sample. Furthermore, there is a large range in mineral concentrations among samples and horns from different rhinos. Together, these findings indicate that rhino horn products ingested by humans are delivering an inconsistent package of compounds. Notably, when horns were sampled deeply, Fe concentrations were relatively high and consistent across sample colors, and in anterior and posterior horns of the same individual, but there was a large range in values among rhinos. Therefore, further study of rhino horn Fe as an IOD biomarker seems feasible and worthy of pursuit. For future studies of factors impacting horn mineral content, we recommend sampling deeply and comparing similarly colored samples to avoid environmental contamination and the variation in several minerals inherent to the heterogeneous composition of rhino horn.

### Supplementary Information

Supplementary Information.

## Data Availability

The datasets generated and/or analyzed during the current study are available from the corresponding author upon reasonable request. Any information identifying individual rhinos or institutions will be removed to ensure the anonymity of those who contributed to this study.
