# Supplementary material for: Rhinoceros horn mineral and metal concentrations vary by sample location, depth, and color
Source: Sci Rep. 2024 Jun 14;14:13808. doi: 10.1038/s41598-024-64472-z (PMC11178811; doi:10.1038/s41598-024-64472-z)
Supplement: Supplementary file 1 — Supplementary Information. [file 41598_2024_64472_MOESM1_ESM.pdf]

## Supplementary Material

## Genomic DNA Extraction Protocols

All extractions were performed using the GeneJET Genomic DNA purification kit (Thermo Fisher Scientific, Waltham, MA, USA) following the manufacturer's instructions. Protocols and modifications utilized for each sample type are detailed below. Molecular biology grade water and 100% ethanol were used to make the necessary buffers as indicated by the kit's manual. For all protocols, the amount of DNA recovered was assessed using a NanoDrop-One spectrophotometer and samples were diluted to 2-15 ng per microliter using TE (10 mM Tris-HCl, 0.1 mM EDTA, pH 8.0) before use.

**Tissue:** Approximately 10 mg of tissue was pulverized with a mortar and pestle chilled with liquid $N_2$ (cryo-cup grinder [BioSpec Products, Bartlesville, OK, USA] cleaned with 10% bleach and water between samples to avoid cross-contamination) and then extracted using the mammalian tissue and rodent tail protocol detailed in the kit's manual. A single modification was made to the procedure, samples were incubated overnight at 56°C with shaking for the digestion step (step 3 of the procedure).

**Sperm:** Approximately 1-10 million sperm cells in 160 µL PBS were extracted using a modification of the cultured mammalian cells protocol detailed in the kit's manual. To each sample was added 20 µL Proteinase K Solution, 40 µL 0.5 M TCEP pH 7.0 (user provided; Tris(2-carboxyethyl)phosphine hydrochloride [C4706, MilliporeSigma] in water), and 200 µL Lysis buffer with vortexing for 15 s between each addition. Samples were incubated with shaking for 1 h at 56°C. The remaining extraction process was performed as detailed in the instructions (steps 4 to 10 of the procedure), with the exception that 100 µL Elution Buffer was used to elute the gDNA from each column.

**Horn:** A portion of the drilled horn coils (≤20 mg) were pulverized with a mortar and pestle chilled with liquid $N_2$ (cryo-cup grinder [BioSpec Products, Bartlesville, OK, USA] cleaned with dilute dish soap and 100% ethanol between samples to avoid cross-contamination) and extracted using a modification of the mammalian tissue and rodent tail protocol. Specifically, samples were incubated in 20 µL Proteinase K Solution, 40 µL 0.5 M TCEP pH 7.0, and 340 µL Digestion buffer overnight with shaking at 56°C. After 16-20 h, 40 µL RNAse Solution was added to each tube. Samples were vortexed until completely mixed before incubating for 10 min at ambient temperature. Then 400 µL Lysis buffer and 270 µL 100% EtOH were added to each sample with vortexing for 15 s in between each addition. Samples were centrifuged for 30 s to pellet any remaining undissolved horn shavings. The supernatant was transferred to a purification column and centrifuged through as per manufacturer instructions. All flow-through and the collection tube were discarded once all the lysate had been passed through the column. The remaining extraction process was performed as detailed in kit's instructions (steps 8 to 11 of procedure), with the exception that 50 - 100 µL Elution Buffer was used to elute the gDNA from each column.

## Sex Identification Assay

**Oligonucleotide Design:** In silico analyses were performed to derive rhino-specific sequences for the predicted proteolipid protein 1 (PLP1), an X-linked gene. The genomic PLP1 sequence (Gene ID 101388648; [1]) obtained from the NCBI annotation (Release 101) of the white rhinoceros genome assembly (RefSeq accession GCF 000283155.1) was used to search the genome assemblies for black, GOH, and Sumatran rhinoceros. Unpublished genome assemblies and sequencing data for GOH rhinoceros were used with permission from the DNA Zoo Consortium (dnazoo.org [2]). The draft assembly was generated by the DNA Zoo team from short, insert-size, PCR-free, DNA-Seq data using w2rap-contigger [3], (see Dudchenko et al., [4] for details). The GOH rhinoceros genome assembly was loaded into Anaconda [5], and packages conda and bioProg were used for decompression prior to blasting with the white rhinoceros sequence and extracting the identified similar genome sequences. Genbank's blastn tool was used to interrogate the black (DicBicMic_v2_BIUU_UCD; [6]) and Sumatran (ASM284483v1 and NRM_Dsumatrensis_v1; [7,8]) rhinoceros assemblies and obtain the black and Sumatran rhino PLP1 genomic sequences. The multiple alignment program (MAFFT ver 7; [9]) was utilized to align the rhino sequences (Supplementary Fig. S1). Primers were designed against region 12,968 – 13,550 bp, which corresponds to exon 7 of the PLP1 gene, using the web application of Integrated DNA Technologies (IDT; Coralville, IA, USA). The Primer-BLAST site [10] was used to ensure that the identified oligonucleotide sets would amplify only the target gene, blast searches were performed against Refseq genomes within the NCBI database for rhinoceroses (taxid:9803) and equids (taxid:9796). Primers and probe (Supplementary Table S1) were synthesized by IDT with probe modified to have a 5' fluorescent tag (SUN) and two dye-quenchers (internal ZEN™ tag and 3' Iowa Black tag).

A similar approach as detailed above for the PLP1 oligonucleotides was utilized to identify the oligonucleotides needed to amplify the rhino sex determining region Y (SRY) gene. The equine sequence (Gene ID 100033824; [11]) was used to search the genome assemblies for black, white, GOH, and Sumatran rhinoceros. Black, GOH and Sumatran rhino sequences identified to be like the equine SRY were then aligned (Supplementary Fig. S2) and oligonucleotides designed. At the time of primer design, a sequence for the white rhino could not be located. Based on previous work by Stoops *et al.* (2018; [12]) it was expected that white rhino SRY sequence would not differ greatly from the other rhino species. Primers and probe (Supplementary Table S1) were synthesized by IDT with the probe modified to have a 5' fluorescent tag (6FAM) and two dye-quenchers (internal ZEN™ tag and 3' Iowa Black tag).

**PCR conditions**: A 20 µL reaction was comprised of 5 µL gDNA (diluted to between 2ng/µL and 10ng/µL), 0.8 µL primers (200 nM final concentration each primer), 1 µL probe (250 nM final concentration each probe), 3.25 µL nuclease-free $H_2O$ and 10 µL 2X Taqman Fast Advance Master Mix for qPCR (Applied Biosystems; ABI). Controls and unknowns were assessed in duplicate. Thermocycler was set to presence/absence for experiment type and to standard mode to run the following conditions: 1 cycle of 30 s at 60°C (fluorescence monitored, pre-read cycle), 1 cycle of 10 min at 95°C (enzyme activation cycle), 36 cycles of 15 s at 95° and 60 s at 58°C (amplification cycles), and 1 cycle of 30 s at 60°C (fluorescence monitored, post-read cycle).

**Species Identification Assay**

**PCR conditions**: A 25 µL reaction was comprised of 1X DreamTaq buffer. 200 nM dNTPs, 80 nM Rh-WR primers (final concentration each primer), 56 nM Rh-BR primers (final concentration each primer), 28 nM Rh-GOH primers (final concentration each), 2 – 15 ng gDNA and 0.625 U of DreamTaq Polymerase. An Eppendorf Mastercycler nexus gradient instrument (Enfield, CT, USA) was utilized to produce PCR products. Conditions consisted of 1 cycle of 5 min at 95°C, 10 cycles of 30 s at 95°C, 30 s at 70°C with a decrease of 1°C each cycle (touchdown) and 30 s of 72°C, 30 cycles of 30 s at 95°C, 30 s at 59°C and 30 s of 72°C, 1 cycle of 5 min at 72°C, and a hold at 10°C until removal from the instrument.

**Gel evaluation:** A portion (5-10 µL) of each reaction was mixed with a GelRed® nucleic acid prestain (Biotium; Fremont, CA, USA) before loading onto a 4% agarose-TAE gel. After 2-4 h of run time at 80V, the gel was imaged on an Odyssey Fc instrument (Li-Cor Biosciences; Lincoln, NE, USA) and bands sizes determined using Image Studio™ (ver. 5.2; Li-Cor). Each gel included at least one marker lane containing 100 bp DNA ladder (Thermo Fisher) to use for calibration of software.

**Individual Identification Assay**

**Validation of 3-primer system:** For each of the 23 loci, singleplex reactions were assembled using gDNA from known black, white and GOH rhinos. A NTC was also prepared substituting TE for gDNA. Each 10 µL reaction consisted of 1X DreamTaq buffer, 200 nM dNTPs, 0.25 U DreamTaq polymerase, 100 nM forward tailed primer, 200 nM reverse primer, 200 nM universal primer (matched to forward primer 5'tail), and ~6 ng gDNA (TE only for NTC). Reactions were run on an Eppendorf Mastercycler nexus gradient instrument using the following conditions: 1 cycle of 3 min at 95°C, 30 cycles of 30 s at 95°C, 30 s at 58°C and 1 min at 72°C, and 1 cycle of 3 min at 72°C. Gel electrophoresis was performed to assess whether appropriately sized products were produced from gDNA and no amplification occurred for the NTC reactions. Once confirmed that primer sets were compatible, multiplex reactions were assembled and conditions optimized to minimize non-specific product formation while maximizing production of target loci. It was determined that a 2-round PCR approach (Supplementary Fig. S3) met the requirements the best.

**Multiplex Panel PCR and capillary electrophoresis:** A 10 µL reaction consisted of 1X DreamTaq buffer, 200 nM dNTPs, 0.25 U DreamTaq polymerase, 50 nM forward tailed primer, 200 nM reverse primer, 200 nM universal primer (matched to forward primer 5'tail), and 2 – 15 ng gDNA (TE only for NTC). Round 1 reactions were run on an Eppendorf Mastercycler nexus gradient instrument set to following conditions: 1 cycle of 3 min at 95°C, 10 cycles of 30 s at 95°C, 30 s at 68°C with a decrease of 1°C each cycle (touchdown) and 1 min of 72°C, 20 cycles of 30 s at 95°C, 30 s at 58°C and 1 min of 72°C, 1 cycle of 3 min at 72°C, and a hold at 10°C until removal from the instrument. For round 2, 5 µL of a master mix consisting of 1X DreamTaq buffer. 200 nM dNTPs, 200 nM labeled universal primers (final concentration for each of the 4 primers), and 0.25U DreamTaq polymerase were added to each round 1 reaction. Tubes were returned to thermal cycler and subjected to 1 cycle of 3 min at 95°C, 10 cycles of 30 s at 95°C, 30 s at 60°C, and 1 min of 72°C, 1 cycle of 3 min at 72°C, and a hold at 10°C until removal from the instrument. PCR product from each reaction (3 µL) was combined with 9.7 µL Hi-Di™ formamide and 0.3 µL GeneScan™ 500 LIZ size standard (Life Technologies) and denatured for 5 min at 95°C before being run on an ABI 3500 Genetic Analyzer.

The resulting data were loaded into the Fisher Connect cloud platform (Thermo Fisher) and analyzed using cloud-based peak scanner (PS app, ver. 3.1.1-PRC-build07; Thermo Fisher) and microsatellite analysis software (MSA app, ver. 1.2-PRC-build08; Thermo Fisher). First, peaks for each

locus were identified using the PS app and internal size standard. Then the identified peaks were analyzed using the MSA app loaded with panels for each multiplex reaction comprised with fixed bin sizes for each locus (Supplementary Table S3) to determine and standardize the allele calls between samples. The resultant profiles were then compared for commonalities in allele assignment patterns between horn samples. Samples that match across all four panels were determined to originate from the same individual.

**References**


[1] Gene [Internet]. Bethesda (MD): National Library of Medicine (US). National Center for Biotechnology. 2004 - Gene ID 101388648 Southern White Rhino Proteolipid protein 1 (PLP1). Gene [Cited 2020 Nov 30] n.d. https://www.ncbi.nlm.nih.gov/gene/101388648.

[2] Dudchenko O, Batra SS, Omer AD, Nyquist SK, Hoeger M, Durand NC, et al. De novo assembly of the Aedes aegypti genome using Hi-C yields chromosome-length scaffolds. Science (80- ) 2017;356:92–5. https://doi.org/10.1126/SCIENCE.AAL3327.

[3] Clavijo BJ, Venturini L, Schudoma C, Accinelli GG, Kaithakottil G, Wright J, et al. An improved assembly and annotation of the allohexaploid wheat genome identifies complete families of agronomic genes and provides genomic evidence for chromosomal translocations. Genome Res 2017;27:885–96. https://doi.org/10.1101/GR.217117.116/-/DC1.

[4] Dudchenko O, Shamim MS, Batra SS, Durand NC, Musial NT, Mostofa R, et al. The Juicebox Assembly Tools module facilitates de novo assembly of mammalian genomes with chromosome-length scaffolds for under $1000. BioRxiv 2018:254797. https://doi.org/10.1101/254797.

[5] Anaconda Software Distribution 2020. https://docs.anaconda.com/.

[6] Assembly [Internet]. Bethesda (MD): National Library of Medicine (US) NC for BI. 2012 - Genome Assembly DicBicMic_v2_BIUU_UCD [Cited 2023 Aug 9] n.d. https://www.ncbi.nlm.nih.gov/datasets/genome/GCA_004027315.2/.

[7] Assembly [Internet]. Bethesda (MD): National Library of Medicine (US) NC for BI. 2012 - Genome Assembly ASM284483v1 [Cited 2023 Aug 9] n.d. https://www.ncbi.nlm.nih.gov/assembly/GCA_002844835.1.

[8] Assembly [Internet]. Bethesda (MD): National Library of Medicine (US) NC for BI. 2012 - Genome Assembly NRM-Dsumatrensis_V1 [Cited 2023 Aug 9] n.d. https://www.ncbi.nlm.nih.gov/assembly/GCA_014189135.1.

[9] Katoh K, Rozewicki J, Yamada KD. MAFFT online service: multiple sequence alignment, interactive sequence choice and visualization. Brief Bioinform 2019;20:1160–6. https://doi.org/10.1093/BIB/BBX108.

[10] Ye J, Coulouris G, Zaretskaya I, Cutcutache I, Rozen S, Madden TL. Primer-BLAST: a tool to design target-specific primers for polymerase chain reaction. BMC Bioinformatics 2012;13:134. https://doi.org/10.1186/1471-2105-13-134/FIGURES/5.

[11] Of G [Internet]. B (MD). NL of M (US). NC for BI. 2004 - Gene ID 100033824 Equus caballus (horse) sex determining region Y (SRY). Gene [Cited 2020 Nov 30] n.d. https://www.ncbi.nlm.nih.gov/gene/1000033824.

[12] Stoops MA, Winget GD, DeChant CJ, Ball RL, Roth TL. Early fetal sexing in the rhinoceros by detection of male-specific genes in maternal serum. Mol Reprod Dev 2018;85:197–204. https://doi.org/10.1002/mrd.22953.

**Supplementary Fig. S1.** Multiple sequence alignment of the proteolipid protein (PLP1) for Sumatran (Sum), black (Blk), white (Wht) and greater one-horned (GOH) rhinos. An asterisk indicates a position that is fully conserved across the 4 species. Oligonucleotide sequences used for qPCR assay indicated in red with bolded text to denote primer locations and underlined text for the probe location.

```
SumPLP1    GAGAAGAGGACAAAGATACTCAGAGAGAAAAAGTAAAGGACCGAAGAAGGAGACTGGAGA    60
BlkPLP1    GAGAAGAGGACAAAGATACTCAGAGAGAAAAAGTAAAGGACAGAAGAAGGAGACTGGAGA    60
WhtPLP1    GAGAAGAGGACAAAGATACTCAGAGAGAAAAAGTAAAGGACTGAAGAAGGAGACTGGAGA    60
GOHPLP1    GAGAAGAGGACAAAGATACTCAGAGAGAAAAAGTAAAGGACCGAAGAAGGAGACTGGAGA    60
           ***************************************** ******************

SumPLP1    GACCAGGATCCTTCCAGCTGAACAAAGTCAGCCGCAAAGCAGACTAGCCAGCCGGCTACA    120
BlkPLP1    GACCAGGATCCTTCCAGCTGAACCAAGTCAGCCGCAAAGCAGACTAGCCAGCCGGCTACA    120
WhtPLP1    GACCAGGATCCTTCCAGCTGAACCAAGTCAGCCGCAAAGCAGACTAGCCAGCCGGCTACA    120
GOHPLP1    GACCAGGATCCTTCCAGCTGAACAAAGTCAGCCGCAAAGCAGACTAGCCAGCCGGCTACA    120
           *********************** ************************************

SumPLP1    ATTGGAGTCAGAGTCCCAAAGACATGGGTGAGTTGCAAAAACTTTAGCATGGAAGATTCA    180
BlkPLP1    ATTGGAGTCCGAGTCCCAAAGACATGGGTGAGTTGCAAAAACTTTAGCATGGAAGATTCA    180
WhtPLP1    ATTGGAGTCCGAGTCCCAAAGACATGGGTGAGTTGCAAAAACTTTAGCATGGAAGATTCA    180
GOHPLP1    ATTGGAGTCAGAGTCGCAAAGACATGGGTGAGTTGCAAAAACTTTAGCATGGAAGATTCA    180
           ********* ***** ********************************************

SumPLP1    AGCAGGCACAAGAATTTGCAAGAGGATTCCCAACTTTGGGGTTCAGAGTTTCCAATGGTT    240
BlkPLP1    AGCAGGCACAAGAATTTGCAAGAGGATTTCCAACTTTGGGGTTCGGAGTTTCCAATGGTT    240
WhtPLP1    AGCAGGCACAAGAATTTGCAAGAGGATTTCCAACTTTGGGGTTCGGAGTTTCCAATGGTT    240
GOHPLP1    AGCAGGCACAAGAATTTGCAAGAGGATTTCCAACTTTGGGGTTCGGAGTTTCCAATGGTT    240
           **************************** ************** ***************

SumPLP1    TAAATGAGTTGTGTTTTGGCACTTGTTTTTTTTTTTTAATTCCTTAGCACCCAATTAGAT    300
BlkPLP1    TAAATGAGTTGTGTTTTGGCACTT---TTTTTTTTTTAATTCCTTAGCACCCAATTAGAT    297
WhtPLP1    TAAATGAGTTGCGTTTTGGCACTT----TTTTTTTTTAATTCCTTAGCACCCAATTAGAT    296
GOHPLP1    TAAATGAGTTGTGTTTTGGCACTT---GTTTTTTTTTAATTCCTTAGCACCCAATTAGAT    297
           *********** ************    ********************************
```

```
SumPLP1    TAACTCCTTCTCTTCTGTACTGAGAGTGATGCTTTATTGCCAAAGAAATCCAGCAGCACG    360
BlkPLP1    TAACTCCTTCTCTGCTGTACTGAGAATGATGCTTTATTGCCAAAGAACTCCAACAGCACG357
WhtPLP1    TAACTCCTTCTCTGCTGTACTGAGAATGATGCTTTATTGCCAAAGAACTCCAGCAGCACG    356
GOHPLP1    TAACTCCTTCTCTGCTGTACTGAG--TGATGCTTTATTGCCAAAGAACTCCAGCAGCACG 355
           ************* **********  ******************** **** *******

SumPLP1    TGGGGAGTCCCACAGATCCTTGGAGAGAGTGCCCCGTGGGCTGCATGTGGTCATGTGTCC    420
BlkPLP1    TGGGGAGTCCCACAGATCCTTGGAGAGAGTGCCCCGTGGGCTGCATGCGGTCATGTGTCC    417
WhtPLP1    TGGGGAGTCCCACAGATCCTTGGAGAGAATGCCCCGTGGGCTGCATGCGGTCATGTGTCC    416
GOHPLP1    TGGGGAGTCCCACAGATCCTTGGAGAGAGTGCCCCGTGGGCTGCATGTGGTCATGTGTCC    415
           **************************** ****************** ************

SumPLP1    TCCTACCCCCTCACTTCCCTCTACCTTCTCTTTTCCCCTGGTTCCCAGGCCCTGGTAATG  480
BlkPLP1    TCCTACCCCCTCACTTCCCTCTACCTTCTCTTTTCCCCTGGTTCCCAGGCCCTGGTAATG  477
WhtPLP1    TCCTACCCCCTCACTTCCCTCTACCTTCTCTTTTCCCCTGGTTCCCAGGCCCTGGTAATG  476
GOHPLP1    TCCTACCCCCTCACTTCCCTCTACCTTCTCTTTTCCCCTGGTTCCCAGGCCCTGGTAATG 475
           ************************************************************
```

```
SumPLP1     GCCAGGTCAGCTGACGTCCCTGGTTCTGCTGGAGGCACCTGAGTGTTTGCTAGCCCTCAG     540
BlkPLP1     GCCAGGTCAGCTGATGTCCCTGGTTCTGCTGGAGGCAACTGAGTGTTTGCTAGCCTTCAG537
WhtPLP1     GCCAGGTCAGCTGATGTCCCTGGTTCTGCTGGAGGCAACTGAGTGTTCGCTAGCCTTCAG     536
GOHPLP1     GCCAGGTCAGCTGACGTCCCTGGTTCTGCTGGAGGCAACTGAGTGTTCGCTAGCCTTCAG     535
            ************** ********************* ********** ******* ****

SumPLP1     ATGTACAAAATAACTGCTTGCTTAATACAAAATTTGGACCCTGTTTGGCATCAGAAGGAA     600
BlkPLP1     ATGTACAAAATAACTGCTTGCTTAATACAAAATTTGGACCCTGTTTGGCATCAGAAGGAA     597
WhtPLP1     ATGTACAAAATAACTGCTTGCTTAATACAAAATTTGGACCCTGTTTGGCATCAGAAGGAA     596
GOHPLP1     ATGTACAAAATAACTGCTTGCTTAATAAAAAAATTTGGACCCTGTTTGGCATCAGAAGGAA     595
            *************************** ********************************

SumPLP1     ATTGGGGAATTGGTCTAGATGGACTCAGGCTTGGCCATGGCTGAGGAGGCCAATCTCAGG     660
BlkPLP1     ATTGGGGAATTGGGCTAGATGGACTCAGGCTTGGCCATGGCTGAGGAGGCCAATCTCAGG     657
WhtPLP1     ATTGGGGAATTGGGCTAGATGGACTCAGGCTTGGCCATGGCTGAGGAGGCCAATCTCAGG     656
GOHPLP1     ATTGGGGAATTGGGCTAGATGGACTCAGGCTTGGCCATGGCTGAGGAGGCCAATCTCAGG     655
            ************* **********************************************

SumPLP1     TTCAAGAAATGAAAATAAAGTCCTCAATGGCTTTACCATGGGGAACGGATATGGGTAGGG     720
BlkPLP1     TTCAAGAAATGAAAATAAAGTCCTCAATGGCTTTACCATGGGGAACGGATATGGGTAGGG     717
WhtPLP1     TTCAAGAAATGAAAATAAAGTCCTCAATGGCTTTACCATGGGGAACGGATATGGGTAGGG     716
GOHPLP1     TTCAAGAAATGAAAATAAAGTCCTCAATGGCTTTACCATGGGGAATGGATATGGGTAGGG     715
            ********************************************* **************

SumPLP1     GGCTGCGTGTGTGTATGTGTATGTGCACATGTATAAGTGACCATATCTTGCAAATATCAG     780
BlkPLP1     GGGTGCGTGTGTGTATGTGTATGTGCACGTGTATAAGTGATCATTTCTTGCAAATATCAG 777
WhtPLP1     GGGTGCGTGTGTGTATGTGTATGTGCACGTGTATAAGTGATCATTTCTTGCAAATATCAG     776
GOHPLP1     GGGTGCGTGTGTGTATGTGTATGTGCACGTGTATAAGTGACCATATCTTGCAAATATCAG     775
            ** ************************* *********** *** ***************

SumPLP1     ACTTAGCTTTCCCAAGGACATGGCTCAGACTCTAAATCCCTGCTATGACAGGAGGCTGCT     840
BlkPLP1     ACTTAGCTTTCCCGAGGACATGGCTCAGACTCTAAATCCCTGCTATGACAGGAGGCTGCT     837
WhtPLP1     ACTTAGCTTTCCCGAGGACATGGCTCAGACTCTAAATCCCTGCTATGACAGGAGGCTGCT     836
```

GOHPLP1 ACTTAGCTTTCCCAAGGACATGGCTCAGACTCTAAATCCCTGCTATGACAGGAGGCTGCT 835
************* **********************************************

SumPLP1 GGAACCTTAGGGCTCTTGCTGACTCAGTTGAGTATTGAGGGCTTCTCTAGGTTTCATGAA 900
BlkPLP1 GGAATCTTAGGGCTCTTGCTGACTCAGTTGAGTATTGAGGGCTTCTCCAGGTTTCATGAA 897
WhtPLP1 GGAACCTTAGGGCTCTTGCTGACTCAGTTGAGTATTGAGGGCTTCTCCAGGTTTCATGAA 896
GOHPLP1 GGAACCTTAGGGCTCTTGCTGACTCAGTTGAGTATTGAGGGCTTCTCCGGTTTTATGAA 895
**** ******************************************  * *** *****

SumPLP1 CAGAGAGAAGCTACAAGGTCTGGTCTCTAGTAGTGTCTACTCAGAGCAAGGTGTGCAGAG 960
BlkPLP1 CAGAGAGAAGCTATGAGGTCTGGTCTCTAGTAGTGTCTACTCAGAGCAAGGTGTGCAGAG 957
WhtPLP1 CAGAGAGAAGCTATGAGGTCTGGTCTCTAGTAGTGTCTACTCAGAGCAAGGTGTGCAGAG 956
GOHPLP1 CAGAGAGAAGCTACGAGGTCTGGTCTCTAGTAGTGTCTACTCAGAGCAAGGTGTGCAGAG 955
*************  *********************************************

SumPLP1 CATGAGGGTCTTTGTCCACACAGGATGCCACAGAGATGTGTAGACATTGCTCACATTTGG 1020
BlkPLP1 CACGAGGGTCTTTGCCCACACAGGATGCCACAGAGTTGTGTAGACATTGCTCACATTTGG 1017
WhtPLP1 CACGAGGGTCTTTGTCCACACAGGATGCCACAGAGATGTGTAGACATTGCTCACATTTGG 1016
GOHPLP1 CACGAGGGTCTTTGTCCACACAGGATGCCACAGAGATGTGTAGACATTGCTCACATTTGG 1015
** *********** ******************** ************************

```
SumPLP1     CAAAACAAGAGTAACAGTTTGAATGCATCCTAAATGAAGTGAGAATCAATTGCCCTGTGT     1080
BlkPLP1     CAAAACAAGGGTAACAGTTTGAACGCATCCTAAATGAAGTGAGAATCAATTGCCCCGTGT     1077
WhtPLP1     CAAAACAAGGGTAACAGTTTGAACGCATCCTAAATGAAGTGAGAATCAATTGCCCCGTGT     1076
GOHPLP1     CAAAACAAGGGTAACAGTTTGAATGCATCCTAAATGAAGTGAGAATCAATTGCCCCGTGT     1075
            ********* ************* ******************************* ****

SumPLP1     ATTCACCACCAAATGTAGCACCTAGAAAATTCCAACAACATCCACAGAAATAGGCTCTCA     1140
BlkPLP1     ATTCACCACCAAATGTAGCACCTAGAAAATTCCAACAACATCCACAGAAATAGGCTCTCA     1137
WhtPLP1     ATTCACCACCAAATGTAGCACCTAGAAAATTCCAACAACATCCACAGAAATAGGCTCTCA     1136
GOHPLP1     ATTCACCACCAAATGTAGCACCTAGAAAATTCCAACAACATCCACAGAAATAGGCTCTCA     1135
            ************************************************************

SumPLP1     GTTATAATAAGCTGGTGTCAGCCTTTAGGTTGTTTCATTCTGAAACACATACATGTTCTT 1200
BlkPLP1     GTTATAATAAGCTGGTGTCAGCCTTTAAGTTGTTTCATTCTGAAACACATACATGTTCTT 1197
WhtPLP1     GTTATAATAAGCTGGTGTCACCCTTTAAGTTGTTTCATTCTGAAACACATACATGTTCTT 1196
GOHPLP1     GTTATAATAAGCTGGTGTCAGCCTTTAGGTTGTTTCATTCTGAAACACATACATGTTCTT     1195
            ******************** ****** ********************************

SumPLP1     TGAAGCATGTATTCAGGTAGAATAATTAAGAGGGCAGAGCATGGACTAGAAATCAACACA     1260
BlkPLP1     TGAAGCATGTATTCAGGTAGAATAATTAAGAGGGCAGAGCATGGACTAGAAATCAACACA     1257
WhtPLP1     TGAAGCATGTATTCAGGTAGAATAATTAAGAGGGCAGAGCATGGACTAGAAATCAACACA     1256
GOHPLP1     TGAAGCATGTATTCAGGTAGAATAATTAAGAGGGCAGAGCATGGACTAGAAATCAACACA     1255
            ************************************************************

SumPLP1     ACTAGGTTCGAATCCCAGATCTGCCAAGGTCATGAAGTTCATGACCTTGGGTTGGTCACT     1320
BlkPLP1     ACTAGGTTCGAATCCCAGATCTGCCAAGGTCATGAACTTCCTGACCTTGGGTTGGTCACT 1317
WhtPLP1     ACTAGGTTCGAATCCCAGATCTGCCAAGGTCATGAACTTCCTGACCTTGGGTTGGTCACT     1316
GOHPLP1     AATAGGTTCGAATTCCAGATCTGCCAAGGTCATGAACTTCTTGACCTTGGCTTGGTCACT     1315
            * *********** ********************** *** ********* *********

SumPLP1     GTGAATTATCACTTCATCTTCGGGAAAATGGGGATAACCATAACTGCTTCGTCTACTTTC     1380
BlkPLP1     GTGAATTATCACTTCATCGTCGGGAAAATGGGGATAACCATAACTGCTTCGTCTACTTTC 1377
WhtPLP1     GTGAATTATCACTTCATCATCGGGAAAATGGGGATAACCATAACTGCTTCGTCTACTTTC     1376
```

```
GOHPLP1    GTGAATTATTACTTCATCATCGGGAAAATGGGGATAACCATAACTGTTTCGTCTACTTTC    1375
           ********* ******** **************************** *************

SumPLP1    AGGGTTGTGAAAATCAAGTAATAATGATGTGGAAGTCCTTTGAAAATGGACAAGCTCTAT    1440
BlkPLP1    AGGGTTGTGAAAATCAAGTAATAATGATGTGGAAGTCGTTTGAAAATGGACGAGCTCTAT    1437
WhtPLP1    AGGGTTGTGAAAATCAAGTAATAATGATGTGGAAGTCGTTTGAAAATGGACAAGCTCTAT    1436
GOHPLP1    AGGGTTGTGAAAATCAAGTAATAATGATGTGGAAGTCCTTTGAAAATGGACAAGCTCTAT    1435
           ************************************* ************* ********

SumPLP1    AACAATTGTAAATCATGATAATACCAATTATTCAACAATAATGTGTGTACAGGCTGAATC    1500
BlkPLP1    AACAATTGTAAATCATGATAATACTGATTATTCAACAATAATGCGTGTACAGGCTGGATC    1497
WhtPLP1    AACAATTGTAAATCATGATAATACTGATTATTCAACAATAATGCGTGTACAGGCTGGATC    1496
GOHPLP1    AACAATTGTAAATCATGATAATACCGATTATTCAACAATAATGCGTGTACAGGCTGAATC    1495
           ************************  ***************** ************ ***

SumPLP1    TTACACTTTCCAAAGTGGTTGGGCTTTCTTTGTGTGCACTTTGTGCACTTGGCTAAGAAT 1560
BlkPLP1    TTACACTTTCCAAAGTGGTTGGGCTTTCTTTGTGTGCACTTTGTGCACTTGGCTAAGAAT 1557
WhtPLP1    TTACACTTTCCAAAGTGGTTGGGCTTTCTTTGTGTGCACTTTTTGCACTTGGCTAAGAAT 1556
GOHPLP1    TTACACTTTCCAAAGTGGTTGGGCTTTCTTTGTGTGCACTTTGTGCACTTGGCTAAGAAT    1555
           ****************************************** *****************
```

```
SumPLP1     CATTTGACCAATTGAGTGTGGTCAGGTGAGAGGTGAATGTTGGAGGAACTCAATTCCTGC     1620
BlkPLP1     CATTTGAGCAATTGAGTGTGGTCAGGTGAGAGGTGAATGTTGGAGGAACTCAATTCCTGC     1617
WhtPLP1     CATTTGAACAATTGAGTGTGGTCAGGTGAGAGGTGAATGTTGGAGGAACTCAATTCCTGC     1616
GOHPLP1     CATTTGAGCAATTGAGTGTGGTCAGGTGAGAGGTGAATGTTGGAGGAACTCAATTCCTGC     1615
            ******* ****************************************************

SumPLP1     TTTAGGTAACATCAGGAACCCTTCCTAATGTCAGGGCAGCTGATTAAGTAGGTTGTGAAA     1680
BlkPLP1     TTTAGGTAATATCAGGAACCTTTCCTAATGTCAGGGCAGCTGATTAAGTAGGTTGTGAAT     1677
WhtPLP1     TTTAGGTAATATCAGGAACGTTTCCTAATGTCAGGGCAGCTGATTAAGTAGGTTGTGAAT     1676
GOHPLP1     TTTAGGTAACATCAGGAACCCTTCCTAATGTCAGGGCAGCTGATTAAGTAGGTTGTGAAT     1675
            ********* *********  **************************************

SumPLP1     TGAAAACGGAGAGGGAGTAAAGAAGGATGCTGAATATTACTTCCTCAGGGGATGAAAGCC     1740
BlkPLP1     TGAAAACGGAGAGGGAGTAAAGAAAGATGTTGAATATTACTTTCTCAGGGGATGAAAGCC     1737
WhtPLP1     TGAAAACGGAGAGGGAGTAAAGAAAGATGTTGAATATTACTTTCGCAGGGGATGAAAGCC     1736
GOHPLP1     TGAAAACGGAGAGGGAGTAAAGAAAGATGCTGAATATTACTTCCTCAGGGGATGAAAGCC     1735
            ************************ **** ************ * ***************

SumPLP1     CTGAGGAAAAGGCAGGAACAAATAAGGTGACCACAGACAAGTCCCCTGGGGGTGGGTGAT     1800
BlkPLP1     CTGAGGAAAAGGCAGGAACAAATAAGGTGACCACAGGGAAGTCCCCTGGGGGTGGGTGAT     1797
WhtPLP1     CTGAGGAAAAGGCAGGAACAAATAAGGTGACCACAGGGAAGTCCCCTGGGGGTGGGTGAT     1796
GOHPLP1     CTGAGGAAAAGGCAGGAACAAATAAGGTGACCACAGGGAACTCCCCTGGGGGTGGGTGAT     1795
            ************************************  ** *******************

SumPLP1     ACTTGGGCCGGGTGCACATGATGGAGTAGCCACTGAGTTTTCAGTTATTTGATAACTATC     1860
BlkPLP1     ACTTGGGTCGAGTGCACATGATGGAGTAGCCACTGAGTTTTCAGTTATTTGATAACTATC 1857
WhtPLP1     ACTTGGGCCGGGTGCACATGATGGAGTAGCCACTGAGTTTTCAGTTATTTGATAACTATC     1856
GOHPLP1     ACTTGGGCAGGGTGCACGTGATGGAGTAGCCACTGAGTTTTCAGTTATTTGATAACTATC     1855
            *******  * ****** ******************************************

SumPLP1     TCAAATGCTTTGTAAATAATAGGCCATTTCTAACAAGATACTGACCTGTTAATCCTTATG     1920
BlkPLP1     TCAAATGCTTTGTAAATAATAGGCCATTTCTAACAAGATACTGACCTGTTAATCCTTCTG 1917
WhtPLP1     TCAAATGCTTTGTAAATAATAGGCCATTTCTAACAAGATACTGACCTGTTAATCCTTCTG1916
```

```
GOHPLP1     TCAAATGCTTTGTAAATAATAGGCCATTTCTAACAAGATACTGACCTGTTAATCCTTCTG          1915
            ********************************************************* **

SumPLP1     ATGTGGAGTGTGCTGCTGTTGAACAATTGTAGTAGACAAATTAGATCATGGAATCTGGGA          1980
BlkPLP1     ATGTGGAGTGTGCTGCTGTTGAACAATTGTAGTAGACAAATTAGATCACGGAATCTGGGA          1977
WhtPLP1     CTGTGGAGAGTGCTGCTGTTGAACAATTCTGGTAGACAAATTAGATCACAGAATCTGAGA          1976
GOHPLP1     ATGTGGAATGTGCCGCTGTTGAACAATAGTAGTAGACAAATTAGATCATGGAATCTGGGA          1975
            ******  **** ************* * *****************  ******* **

SumPLP1     TTGGAAGGAACTTTAGAAGCTGTCTGAGCCAACTCTCAATGCAGCAATCCCTTCTGCAAC          2040
BlkPLP1     TTGGAAGGAACTTTAGAAGCTGTCTGAGCCAACTCTCAATGCAGGAATCCCTTCTGCAAC          2037
WhtPLP1     TTGGAAGGAACTTTAGAAGCTGTCTGAGCCAACTCTCAATGCAGGAATCCCTTCTGCAAC          2036
GOHPLP1     TTGGAAGGAACTTTAGAAGCTGTCTGAGCCAACTCTCAATGCAAGAATCCCTTCTGCAAC          2035
            ******************************************  ***************

SumPLP1     AGCCAATTAAACATTCCTACAGTCTCTGCTTGATCACCTCCAGGAATAGGGAGCTCACTA          2100
BlkPLP1     AGCCAATTAAACATTCCTACAGTCTCTGCTTGATCACCTCCAGGAATAGGGAGCTCACTA          2097
WhtPLP1     AGCCAATTAAACATTCCTACAGTCTCTGCTTGATCACCTCCAGGAATAGGGAGCTCACTA          2096
GOHPLP1     AGCCAATTAAACATTCCTACAGTCTCTGCTTGATCACCTCCAGGAATAGGGAGCTCACTA          2095
            ************************************************************
```

```
SumPLP1    CCTCAACCATGTAGAATCTCATAGGTTTCGAAGTGAAGAGGAACCCTAAATTCAGTAGTG    2160
BlkPLP1    CCTCAACCATGTAGAATCTCATAGGTTTGGAAGTGAAGAGGAACCCTGAATTCAGTAGTG    2157
WhtPLP1    CCTCAACCATGTAGAATCTCATAGGTTTGGAAGTGAAGAGGAACCCTGAATTCAGTAGTG    2156
GOHPLP1    CCTCAACCATGTAGAATCTCATAGGTTTGGAAGTGAAGAGGAACCCTGAATTCAGTAGTG    2155
           **************************** ****************** ************

SumPLP1    TCTATGCAAAAAGTTTATGAGGGAAATGATAGTTCATTAAACCGTGTGCTGACTTGGGAG    2220
BlkPLP1    TCTATGCAAAAGGTTTATGAGGGAAATGATAGTTCATTAAACCGTGTGCTGACTTGGGAG    2217
WhtPLP1    TCTATGCAAAAGGTTTATGAGGGAAATGATAGTTCATTAAACCGTGTGCTGACTTGGGAG    2216
GOHPLP1    TCTATGCAAAAGGTTTATGAGGGAAATGATAGTTCATTAAACCGTGTGCTGACTTGGGAG    2215
           *********** ************************************************

SumPLP1    AGGGATGTTTCAAATGAGTCTTTAGGATAACAATGGGACCGGAAAAGAGCCAAAGAGCAG    2280
BlkPLP1    AGGGATGTTTCAAATGAGTCTTTGGGATAACAATGGAACCGGAAAAGAGCCAAAGAGCAG    2277
WhtPLP1    AGGGATGTTTCAAATGAGTCTTTGGGATAACAATGGAACCGGAAAAGAGCCAAAGAGCAG    2276
GOHPLP1    AGGAATGTTTCAAATGAGTCTTTGGGATAACAAT--ACCCGGAAAAGAGCCAAAGAGCAG    2273
           *** ******************* **********    **********************

SumPLP1    TAGCTGATTATTCTCTGCTCAGTAACACAGTGCCATTACTGAACAGCAAAACATTGGCTC    2340
BlkPLP1    TAGCTGATTATTCTCTGCTCAGTAACACAATGCCATTACTGAACAACAAAACATTGGCTC2337
WhtPLP1    TAGCTGATTATTCTCTGCTCAGTAACACAATGCCATTACTGAACAACAAAACATTGGCTC    2336
GOHPLP1    TAGCTGATTATTCTCTGCTCAGTAACACAATGCCATTACTGAACAACAAAACATTGGCTC    2333
           ***************************** *************** **************

SumPLP1    CCTCAAGCCTGTTGTCTGAGAACACTGGCTGTTACCCTGGTAACCAGACTATGATGCTTC    2400
BlkPLP1    CCTCAAGCCTGTTGTCTGAGAACACTGGCTGTTACCCTGGTAACCAGACTATGATGCTTC 2397
WhtPLP1    CCTCAAGCCTGTTGTCTGAGAACACTGGCTTTTACCCTGGTAACCAGACTATGATGCTTC    2396
GOHPLP1    CCTCAAGCCTGTTGTCTGAGAACACTGGCTGTTACCCTGGTAACCAGACTATGATGCTTC    2393
           ****************************** *****************************

SumPLP1    CCTCCAAACGTGGCTGTTTGCCGAGCATTGTAGTTTTGGCTGGCTCAGAGTAAGTGGGGG    2460
BlkPLP1    CCTCCAAACGTGGCTGTTTGCCGAGCAGTGTAGTTTTGGCTGGCTCAGAGTAAGTGGGGG    2457
WhtPLP1    CCTCCAAACGTGGCTGTTTGCCGAGCAGTGTAGTTTTGGCTGGCTCAGAGTAAGTGGGGG    2456
```

```
GOHPLP1     CCTCCAAACGTGGCTGTTTGCCGTGCAGTGTAGTTTTGGCTGGCTCAGAGTAAGTGGGGG     2453
            ********************* *** ********************************

SumPLP1     TAGCCCAGGCCAAAAGTGTGGAGGCAGAGCAGGGGTCCAGTTTTACAAAATGCCTGAAAA     2520
BlkPLP1     TAGCCCAGGCCAAAAGTGTGGAGGCAGAGCAGGGGTCCAGTTTTACAAAATGCCTGAAAA     2517
WhtPLP1     TAGCCCAGGCCAAAAGTGTGGAGGCAGAGCAGGGGTCCAGTTTTACAAAATGCCTGAAAA     2516
GOHPLP1     TAGCCCAGGCCAAAAGTGTGGAGGCAGAGCAGGGGTCCAGTTTTACAAAATGCCTGAAAA     2513
            ************************************************************

SumPLP1     CTAACTGAATCCCACCATGATGACAAGAACCCTGCAAGAATGCCAGGAGCAAAAAAGAAA     2580
BlkPLP1     CTAACTGAATCCCACCATGATGACAAGAATCCTGCAAGAATGCCAGGAGCTAAAAAGAAA     2577
WhtPLP1     CTAACTGAATCCCACCATGATGACAAGAATCCTGCAAGAATGCCAGGAGCTAAAAAGAAA     2576
GOHPLP1     CTAACTGAATCCCACCATGATGACAAGAATCCTGCAAGAATGCCAGGAGCTAAAAAGAAA     2573
            ***************************** ******************** *********

SumPLP1     CCAGATCCAAACCAGCATATTCACTCACTCTCTTCCTAGAGACGCTGACCGAAATGATCA     2640
BlkPLP1     CCAGATCCAAACCAGCATATTCACTTACTCTCTGCCTAGAGACGCTGACTGAAATGATCA     2637
WhtPLP1     CCAGATCCAAACCAGCATATTCACTTACTCTCTGCCTAGAGACGCTGACTGAAATGATCA     2636
GOHPLP1     CCAGATCCAAACCAGCATATTCACTTACTCTCTTCCTAGAGACGCTGACTGAAATGATCA     2633
            ************************* ******* *************** **********
```

```
SumPLP1     CGGACCGCCTCCCCCCACTCCCCACCTTGGCACTGCAGAGCCAGAGGTGCTCTTGGAGAC          2700
BlkPLP1     TGGACAGCATCCCCCTACTCCCCACCTTGGCACTGCAGAGCCAGAGGTGCTCTTGGAGAC          2697
WhtPLP1     CGGACAGCCTCCCCCTACTCCCCACCTTGGCACTGCAGAGCCAGAGGTGCTCTTGGAGAC          2696
GOHPLP1     CGGACAGCCTCCCCCCACTCCCCACCTTGGCACTGCAGAGCCAGAGGTGCTCTTGGAGAC          2693
            **** ** ****** *********************************************

SumPLP1     CCAGGCAGATTGGAAGGTCTGCTCTGCTTTGGGGGGCTTTTGCCCTGGCTCTGTGCTAGG          2760
BlkPLP1     CCAGGCAGATTGGAAGGTCTGCTCTGCTTCGGGGGGCTTTTGCCCTGGCTCTGTGCTAGG2757
WhtPLP1     CCAGGCAGATTGGAAGGTCTGCTCTGCTTCGGGGGGCTCTTGCCCTGGCTCTGTGCTAGG          2756
GOHPLP1     CCAGGCAGATTGGAAGGTCTGCTCTGCTTC--GGGGCTTTTGCCCCGGCTCTGTGCTAGG2751
            ****************************   ****** ****** **************

SumPLP1     AAAGCCAGAAGTCTACTCAACAGCCTGTCAATTAGAACTATTCTCTCAAAGCTATCTGAG          2820
BlkPLP1     AAACCCAGAAGTTTACTCAACAGCCTGTCAATTAGAACTATTCTCTCAAAGCTATCTGAG          2817
WhtPLP1     AAACCCAGAAGTTTACTCAATAGCCTGTCAATTAGAACTATTCTCTCAAAGCTATCTGAG          2816
GOHPLP1     AAAGCCAGAAGTTTACTCAACAGCCTGTCAATTAGAACTATTCTCTCAAAGCTATCTGAG          2811
            *** ******** ******* ***************************************

SumPLP1     TAGTAACAAGGCCCCCAAAATGCCTGGGAGATGGGTGGAGGGGAAATATAGTCTCCAAA          2880
BlkPLP1     TAGTAACAAGGCCCCCAAAATGCCTGGGAGATGGGTGGAGGGGAAATATAGTATCCAAA          2877
WhtPLP1     TAGTAACAAGGCCCCCAAAATGCCTGGGAGATGGGTGGAGGGGAAATATAGTATCCAAA          2876
GOHPLP1     TAGTAACAAGGTCCCCAAAATGCCTGGGAGATGGGTGGAGGGGAAATATAGTATCCAAA          2871
            *********** ***************************************** ******

SumPLP1     CCCCTCAAGGCAGAACCCCAGCCAGTGGTATATTTTTCCTTTGTTGATTTTTTGTGTATT 2940
BlkPLP1     CCCATCAAGGCAGAACCCCAGCCAGTGGTATCTTTTT-CTTTGTTGATTTTTTGTGTATT   2936
WhtPLP1     CCCATCAAGGCAGAACCCCAGCCAGTGGTATCTTTTT-CTTTGTTGATTTTTTGTGTATT  2935
GOHPLP1     CCCATCAAGGCAGAACCCCAGCCAGTGGTATCTTTTT-CTTTGTTGATTTTTTGTGTATT 2930
            *** ************************* ***** *********************

SumPLP1     ATCTTGAAGCCCTGAAAATC--ATCCCCAGGGTATAGTTCTTGGTCTTGAAAGAAAGCAA2998
BlkPLP1     ATCTTGAAGCCCTGAAAATC--ATCCCCAGGGTATAGTTCTTGGTCTTGAAAGAAAGCAA 2994
WhtPLP1     ATCTTGAAGCCCTGAAAATC--ATCCCCAGGGTATAGTTCTTGGTCTTGAAAGAAAGCAA2993
```

```
GOHPLP1    ATCTTGAAGCCCTGAAAAACTATACCTCAGGGTATAGTTCTTGGTCTTGAAAGAAAGCAA    2990
           ******************* *  ** **********************************

SumPLP1    CAGTAGAAATGCCCTCCTTGCCTACAGAACCTCAGGTCCAGAGAAGATCAAGTCAGGGCA    3058
BlkPLP1    AAGTAGAAATGCCCTCCTTGCCCACAGAACCTCAGGTCCAGAGAAGATCAAGTCAGGGCA    3054
WhtPLP1    AAGTAGAAATGCCCTCCTTGCCCACAGAACCTCAGGTCCAGAGAAGATCAAGTCAGGGCA    3053
GOHPLP1    CAGTAGAAATGCCCTCCTTGCCCACAGAACCTCAGGTCTAGAGAAGATCAAGTCAGGGCA    3050
           ********************** *************** *********************

SumPLP1    GTGACATGATCAGAAAGGGCCACATCAGCCATGGTAGATAAACAGAGCAGCTTTCCCTTC    3118
BlkPLP1    GTGACATGATCAGAAAGGGCCACATCAGCCATGGTAGATGAACAGAGCAGCTTTCCCTTC    3114
WhtPLP1    GTGACATGATCAGAAAGGGCCACATCAGCCATGGTAGATGAACAGAGCAGCTTTCCCTTC    3113
GOHPLP1    GTGACATGATCAGAAAGGGCCACATCAGCCATGGTAGACGAACAGAGCAGCTTTCCCTTC    3110
           **************************************  ********************

SumPLP1    CTTTCCCACTGTCAGAACCTGCCCAGTGCCAAAGTGACATCAAAAGCTCAGCAAGGACTG    3178
BlkPLP1    CTTTCCCACTGTCAGAGCCTGCCCAGTGCCAAAGTGACATCAAAAGCTCAGCACGGACTG    3174
WhtPLP1    CTTTCCCACTGTCAGAGCCTGCCCAGTGCCAAAGTGACATCAAAAGCTCAGCACGGACTG    3173
GOHPLP1    CTTTCCCACTGTCAGAGCCTGCCCAGTGCCAAAGTGACATCAAAAGCTCAGCAAGGACTG    3170
           **************** ************************************ ******
```

```
SumPLP1   GCTGACTAATTCTTCCTTGGCTATTTTAAAATAGTCCTAAGGGTGATCTCTCAACTCTGC   3238
BlkPLP1   GCTGACTAATTCTTCCTTGGCTATTTTAAAATAGTCCTAAGGGTGATCTCTCAACTCTGC   3234
WhtPLP1   GCTGACTAATTCTTCCTTGGCTATTTTAAAATAGTCCTAAGGGTGATCTCTCAACTCTAC   3233
GOHPLP1   GCTGACTAATTCTTCCTTGGCTATTTTAAAATAGTCCTAAGGGTGATCTCTCAACTCTGC   3230
          ********************************************************** *

SumPLP1   ACTGGCCTCTGAGCTCTCTGCCAGGCAGGGCTCTGGAAAAGAGTCAGTGACATCCCAAGG   3298
BlkPLP1   ACTGGCCTCTGAGCTCTCTGCCAGGCAGGGCTCTGGAAAAGAGTCAGTGACATCCCAAGG   3294
WhtPLP1   ACTGGCCTCTGAGCTCTCTGCCAGGCAGGGCTCTGGAAAAGAGTCAGTGACATCCCAAGG   3293
GOHPLP1   ACTGGCCTCTGAGCTCTCTGCCAGACAGGGCTCTGGAAAAGAGTCAGTGACATCCCAAGG   3290
          ************************ ***********************************

SumPLP1   AAGAGAACAGGTTCCCCAGATCAGCTTCACACTGCAACCAAGGAGAGAAGAGGAATTGGT   3358
BlkPLP1   AAGAGAACAGGTTCCCGAGATCAGCTTCACACTGCAACCAAGGAGAGAGGAGGAATTGGT   3354
WhtPLP1   AAGAGAACAGGTTCCCCAGATCAGCTTCACACTGCAACCAAGGAGAGAGGAGGAATTGGT   3353
GOHPLP1   AAGAGAACAGGTTCCCCAGATCAGCTTCACACTGCAACCAAGGAGAGAGGAGGAATTGGT   3350
          **************** ***************************** *************

SumPLP1   GGGAGACTGACCTAAGGAATAAGGCATGGAAACTGTGGTCTCTTAGCTTAGTGCCAGGTG   3418
BlkPLP1   GGGAGACTGACCTAAGGAATAAGGTGTGGAAACAGTGGTCTCTTAGCTTAGTGCCAGGTG   3414
WhtPLP1   GGGAGACTGACCTAAGGAATAAGGTGTGGAAACAGTGGTCTCTTAGCTTAGTGCCAGGTG   3413
GOHPLP1   GGGAGACTGACCTAAGGAATAAGGTGTGGAAACTGTGGTCTCTTAGCTTAGTGCCAGGTG   3410
          ************************  ******* **************************

SumPLP1   CTTCTCTTTCACCGTCCCATAAGTGGGCCAGGTGAAGACAATGTGCCAGGGATAGAGTTT   3478
BlkPLP1   CTTCTCTTTCACCATCCCATAAGTGGGCCAGGTGAAGACAATGTGCCAGGGACAGAGTTT   3474
WhtPLP1   CTTCTCTTTCACCATCCCATAAGTGGGCCAGGTGAAGACAATGTGCCAGGGATAGAGTTT   3473
GOHPLP1   CTTCTCTTTCACCTTCCCATAAGTGGGCCAGGTGAAGACAATGTGCCAGGGATAGAGTTG   3470
          ************* ************************************** ****** 

SumPLP1   AACAATCAGGCTTCTACCCTAACCTCCCAATGTTTGGGCATAAATTGGCACACG------   3532
BlkPLP1   AACAATCAGGCTGCTACCCTAACCTCCCAATGTTTGGGCATAAATTGGCATACG------   3528
WhtPLP1   AACAATCAGGCTGCTACCCTAACCTCCCAATGTTTGGGCATAAATTGGCATACGTGTGTG   3533
```

```
GOHPLP1     AACAATCAGGCTTCTACCCTAAACTCCCAATGTTTGGGCATAAATTGGCATAC------G    3524
            ************ ********* **************************** **

SumPLP1     ---------------------TGTGTGTGTGTGTGTGTGTGTGTGTGTGTGTTGGGTAG           3570
BlkPLP1     --TGTGTGTGTGTGTGTGTGTGTGTGTGTGTGTGTGTGTGTGTGTGTGTGTGTTGGGGAG    3586
WhtPLP1     TGTGTGTGTGTGTGTGTGTGTGTGTGTGTGTGTGTGTGTGTGTGTGTGTGTGTTGGGGAG3593
GOHPLP1     TTTGTGTGTGTGTGTGTGTGTGTGTGTGTGTGTGTGTGTGTGTGTGTGTGTTGGGGAG          3584
                       ************************************ **

SumPLP1     GAGTGGGGAGTATTGGAAACAAGGTAGTCCAGATGCTGTTGCCATGGTAATAATAATATC        3630
BlkPLP1     GAGTGGGGAGTATTGGAAACAAGGTAGTCCAGATGCTGTTGCCATGGTAATAATAATATC        3646
WhtPLP1     GAGTGGGGAGTATTGGAAACAAGGTAGTCCAGATGCTGTTGCCATGGTAATAATAATATC        3653
GOHPLP1     GAGTGGGGAGTATTGGAAACAAGGTAGTCCAGATGCTGTTGCCATGGTAATAATAATATC        3644
            ************************************************************

SumPLP1     TGTCCAAAGAAGAGGCTGAGATTCCTTTTTCTTCAATGCTGTCCACATGACTTGACTACC        3690
BlkPLP1     TGTCCAAAGAAGAGGCTGAGATTCCTTTTTCTTCAATGCTGTCCACATGACTTGACTACC 3706
WhtPLP1     TGTCCAAAGAAGAGGCTGAGATTCCTTTTTCTTCAATTCTGTCCACATGACTTGACTACC3713
GOHPLP1     TGTCCAAAGAAGAGGCTGAGATTCCTTTTTCTTCAATGCTGTCCACATGACTTGACTACC        3704
            ************************************* **********************
```

```
SumPLP1     CAGCAGCCTTGGAATCTCATATCCCAGCGACTGCAGGGCCCCTTTCCAGCACTTGAATGT     3750
BlkPLP1     CAGCAGGCTTGGAGCCTCATATCCCAGCGACTGCAGGGCCCCTTTCCAGCACTTGAATGT     3766
WhtPLP1     CAGCAGGCTTGGAGTCTCATATCCCAGCGACTGCAGGGCCCCTTTCCAGCACTTGAATGT     3773
GOHPLP1     CAGCAGGCTTGGAATCTCATATCCCAGACACTGCAGGGCCCCTTTCCAGCACTTGAATGT     3764
            ****** ******  ************  ********************************

SumPLP1     GGTATAAGTGCTGATTTCATGTAGGAAAGACAAGATTATAGGCA-GGCTTTGTCTGACCA     3809
BlkPLP1     GGTATAAGTGCTGATTTCATGTAGGAAAGACAAGATTATAGGCAGGGCTTTGTCTGACCA     3826
WhtPLP1     GGTATAAGTGCTGATTTCATGTAGGAAAGACAAGATTATAGGCAGGGCTTTGTCTGACCA     3833
GOHPLP1     GGTATAAGTGCTGATTTCATGTAAGAAAGACAAGATTATAGGCAGGGCTTTGTCTGACCA     3824
            *********************** ******************** ***************

SumPLP1     GATAAGGCCAGAAGCAAAAGTCTTTGTCTCTGGTACCTCCTGATGATTGTTGTTTTACTC     3869
BlkPLP1     GATAAGGCCAGAAGCAAAAGTCTTTGTCTCTGGTACCTCCTGATGATTGTTGTTTTACTC 3886
WhtPLP1     GATAAGGCCAGAAGCAAAAGTCTTTGTCTCTGGTACCTCCTGATGATTGTTGTTTTACTC3893
GOHPLP1     GATAAGGCCAGAAGCAAAAGTCTTTGTCTCTGGTACCTCCTGATGATTGTTGTTTTACTC     3884
            ************************************************************

SumPLP1     CTAGAACCCACCCACTGATACCCATCCATCCCCAGTACCAGTCACGGCTGCTCTAGCATC     3929
BlkPLP1     CTAGAACCCACTCACTGATCCCCATCCATCCCCAGTACCAGTCACGGCTGCTCTAGCATC3946
WhtPLP1     CTAGAACCCACCCACTGATCCCCATCCATCCCCAGTACCAGTCACGGCTGCTCTAGCATC     3953
GOHPLP1     CTAGAACCCACCCACTGATCCCCATCCATCCCCAGTACCAGTCACGGCTGCTCTAGCATC     3944
            *********** ******* ****************************************

SumPLP1     TTGGCCTTCTCAACTGCTATAGGCCCTGATCAAGGACGCCTCTGTTCATGGGCTGCGGTT     3989
BlkPLP1     TTGGCCTTCTCAACTGCTATAGGCCCTGATCAAGGACGCCTCTGTTCATGGGGTGAGGTT4006
WhtPLP1     TTGGCCTTCTCAACTGCTATAGGCCCTGATCAAGGACGCCTCTGTTCATGGGGTGAGGTT     4013
GOHPLP1     TTGGCCTTCTCAACTGCTATAGGCCCTGATCAAGGACGCCTCTGTTCATGGGGTGAGGTT     4004
            **************************************************** ** ****

SumPLP1     AAGGGGTGGGTTCTGGGAGGAGGGTAAGACTTTAGTGCAGATTAGCAACTTAGGGAGATG     4049
BlkPLP1     AAGGGGTGGGTTCTGGGAGGAGGGTAAGACTTTAGTGCAGATTAGAGACTTAGGGAGATG     4066
WhtPLP1     AAGGGGTGGGTTCTGGGAGGAGGGTAAGACTTTAGTGCAGATTAGAGACTTAGGGAGATG     4073
```

```
GOHPLP1     AAGGGGTGGGTTCTGGGAGGAGGGTAAGACTTTAGTGCAGATTAGAGACTTAGGGAGATG     4064
            *********************************************  *************

SumPLP1     GGGCCAGTTTTTCACTCAATGAGCCTGCCTGTTGTTTAGCTCTATTGTATAGGGTTCTTC 4109
BlkPLP1     GGGCCAGTTTTTCACTCAATGAGCCTGCCTGTTGTTTAGCTCTATTGTATAGGGTTCTTC 4126
WhtPLP1     GGGCCAGTTTTTCACTCAATGAGCCTGCCTGTTGTTTAGCTCTATTGTATAGGGTTCTTC 4133
GOHPLP1     GGGCCAGTTTTTCACTCAATGAGCCTGCCTGTTGTTTAGCTCTATTGTATAGGGTTCTTC     4124
            ************************************************************

SumPLP1     TATCTCTCCCTTTGTCAGCTGGATTCAAGGGACAGGCTGGGAATTGTGCAAGGCACAATT     4169
BlkPLP1     TATCTCTCCCTTTGTCAGCTGGATTCAAGGGACAGGCTGGGAATTGTGCAAGGCACAATT     4186
WhtPLP1     TATCTCTCCCTTTGTCAGCTGGATTCAAGGGACAGGCTGGGAATTGTGCAAGGCACAATT     4193
GOHPLP1     TATCTCTCCCTTTGTCAGCTGGATTCAAGGGACAGGCTGGGAATTGTGCAAGGCACAATT     4184
            ************************************************************

SumPLP1     GGTCTCTTTCTACCATCCCTTTCCATGAAAGCCCTTCCTCTCCTCCCTTTGATGAGTTCC 4229
BlkPLP1     GCTCTCTTTCTACCATCCCTTTCGATGAAAGCCCTTCCTCTCCTCCCTTTGATGAGTTCC 4246
WhtPLP1     GCTCTCTTTCTACCATCCCTTTCGATGAAAGCCCTTCCTCTCCTCCCTTTGATGAGTTCC 4253
GOHPLP1     GGTCTCTTTCTACCATCCCTTTCGATGAAAGCCCTTCCTCTCCTCCCTTTGATGAGTTCC 4244
            * ******************** *************************************
```

```
SumPLP1    CAGAGTCACACTGAATTTCCATCATGGAGCTGGTCCTTGAGGCGTGCCCAGGGCATGGGA    4289
BlkPLP1    CAAATTCACACTGAATTTCCATCATGGAGCTGGTCCTTGAGGCGTGCCCAGGGCATGGGA    4306
WhtPLP1    CAAATTCACACTGAATTTCCATCATGGAGCTGGTCCTTGAGGCATGCCCAGGGCATGGGA    4313
GOHPLP1    CAGATTCACACTGAATTTCCATCATGGAGCTGGTCCTTGAGGCGTGCCCAGGGCATGGGA    4304
           ** * ************************************** ****************

SumPLP1    AAGGAGGGAGGGCTTGGGAGCTCTTGGCTCCTGCTTAAAGAGACAGCATCAGGATCAACA    4349
BlkPLP1    AAGGAGGGAGGGCTTGGGAGCTCTTGGCTCCTGCTTAAACAGACAGCATCAGGATCAACA    4366
WhtPLP1    AAGGAGGGAGGGCTTGGGAGCTCTTGGCTCCTGCTTAAACAGACAGCATCAGGATCAACA    4373
GOHPLP1    AAGGAGGGAGGGCTTGGGAGCTCTTGGCTCCTGCTTAAACAGACAGCATCAGGATCAACA    4364
           *************************************** ********************

SumPLP1    TAGTCCCTTGGATAGACATGGATCTGGATAAGAGTCAGAACGGTCCTGTTGATCTCTGTT    4409
BlkPLP1    TAGTCCCTTGGATGGACATGGATCTGGATAAGAGTCAGAACGATCCTGTTGATCTCTGTC    4426
WhtPLP1    TAGTCCCTTGGATGGACGTGGATCTGGATAAGAGTCAGAACGATCCTGTTGATCTCTGTC    4433
GOHPLP1    TAGTCCCTTGGATGGACATGGATCTGGATAAGAGTCAGAACGATCCTGTTGATCTCTGTC    4424
           ************* *** ************************ ****************

SumPLP1    AGACTACTGTGAAGAGCATTGCCAAGAAGGGCTTGAAGGTCTATCACAGATTGATTAGAC    4469
BlkPLP1    AGACTCCTGTGAAGAGCATTGCCAAGAAGGGCTTGAAAGTCTATCACAGATTGATCAGAC    4486
WhtPLP1    AGACTCCTGTGAAGAGCATTGCCAAGAAGGGCTTGAAAGTCTATCACAGATTGATCAGAC    4493
GOHPLP1    AGACTCCTGTGAAGAGCATTGCCAAGAAGGGCTTGAAAGTCTATCACAGATTGATTAGAC    4484
           ***** ******************************* ***************** ****

SumPLP1    TAGTTTTGAGAGTCTGTAGATAAATCTAGCCAACACTGAATATTTCCCTTTTGCTTACAT 4529
BlkPLP1    TAGTTTTGAGAGTCTGTAGATAAATCTAGCCAACACTGAATATTTCCCTTTTGCTTACAT 4546
WhtPLP1    TAGTTTTGAGAGTCTGTAGATAAATCTAGCCAACACTGAATATTTCCCTTTTGCTTACAT 4553
GOHPLP1    TAGTTTTGAGAGTCTGTAGATAAATCTGGCCAACACTGAATATTTCCCTTTTGCTTACAT    4544
           *************************** ********************************

SumPLP1    TTTGCCCACAGGACTTGGTTATAAACATTTTGGCACTAGATTATAGGTAGTTTTTGTCTT 4589
BlkPLP1    TTTGCCCACAGGACTTGGTTATACACATTTTGACACTAGATTATAGGTAGTTTTTGTCTT 4606
WhtPLP1    TTTGCCCACAGGACTTGGTTATACACATTTTGACACTAGATTATAGGTAGTTTTTGTCTT 4613
```

```
GOHPLP1     TTTGCCCACAGGACTTGGTTATACACATTTTGACACTAGATTATAGGTAGTTTTTGTCTT     4604
            ********************** ******** *************************

SumPLP1     TGATTCACAGACATAACTTTATTTTCCCCAATAAGACTGAGGTTTTTGAAGAAAGGGACT     4649
BlkPLP1     TGATTCACAGACATAACTTTATTTTCCCCAATAAGACTGAGGTTTTTGAAGAAAGGGACT     4666
WhtPLP1     TGATTCACAGACATAACTTTATTTTCCCCAATAAGACTGAGGTTTTTGAAGAAAGGGACT     4673
GOHPLP1     TGATTCACAGACATAACTTTATTTTCCCCAATAAGACTGAGGTTTTTGAAGAAAGGGACT     4664
            ************************************************************

SumPLP1     GGGCCTTATAATTCCGTTTTGGTTCTCATAGCGTATAAATTACTGCTAACGTACTTAATA 4709
BlkPLP1     GGGTCTTATAATTCCCTTTTGGTTCTCATAGCGTATAAATTACTGCCAACGTACTTGATA 4726
WhtPLP1     GGGTCTTATAATTCCCTTTTGGTTCTCATAGCGTATAAATTACTGCCAACGTACTTGATA 4733
GOHPLP1     GGGCCTTATAATTCCCTTTTGGTTCTCGTAGCGTATAAATTACTGCTAACGTACTTAATA     4724
            *** *********** *********** ****************** ********* ***

SumPLP1     ATTGTACTGAATCCACATGAGCAGAAATGAAAGGAAAACACTTGGGAGTGTCATTATTTT     4769
BlkPLP1     ATTGTACTGAATCCACATACACAGAAATGAAAGGAAAACACTTGGGAGTGTCATTATTCT     4786
WhtPLP1     ATTGTACTGAATCCACATACGCAGAAATGAAAGGAAAACACTTGGGAGTGTCATTATTTT     4793
GOHPLP1     ATTGTACTGAATCCACATAAGCAGAAATGAAAGGAAAACACTTGGGAGTGTCATTATTTT     4784
            ******************   ************************************* *
```

```
SumPLP1     GCTCTATGACCTCTCATC-AAGTCAGGTGGTTTAATCCAACCTGGGTAATATTACTATAT 4828
BlkPLP1     GCTCTATGACCTCTCATCAAAGTCAGGTGGTTTAATCCAACCTGGGTAATATTACTATAT 4846
WhtPLP1     GCTCTATGACCTCTCCTCAAAGTCAGGTGGTTTAATCCAACCTGGGTAATATTACTATAT4853
GOHPLP1     GCTCTATGACCTCTCATCAAAGTCAGGCGGTTTAATCCAACCTGGGTAATATTACTATAT 4844
            ************** ** ******** *********************************

SumPLP1     TAGCATTGGGTAATATTACTATATAGTTTTTTATGCCAACTCTACCATGTTGCAGTCTG 4888
BlkPLP1     TAGCATTGGGTAATATCACTATATAGTTTTTTATGCCAACGCTACCACGTTGCAGTCTG 4906
WhtPLP1     TAGCATTGGGTAATATTACTATATAGTTTTTTATGCCAACGCTACCACGTTGCAGTCTG 4913
GOHPLP1     TAGCATTGGGTAATATTACTATATAGTTTTTTATGCCAACTCTACCATGTTGCAGTCTG 4904
            *************** ********************** ****** ***********

SumPLP1     TACTTAGCCTGGAAATGGGAGAATGTCTCCTGAGAGAGCCAAGGAACTTCTCTAGAATCC 4948
BlkPLP1     TACTTAGCCTGGAAATGGGAGAATGTCTCCTGAGAGAGCTAAGGAACTTCTCTAAAATCC 4966
WhtPLP1     TACTTAGCCTGGAAATGGGAGAATGTCTCCTGAGAGAGCTAAGGAACTTCTCTAAAATCC 4973
GOHPLP1     TACTTAGCCTGGAAATGGGAGAATGTCTCCTGAGAGAGCCAAGGAACTTCTCTAGAATCC 4964
            ************************************** ************* *****

SumPLP1     CTCACAGCTTTCCTGAATCAAACCTATACCATTTCAATGATGGATTCTGAAATGGAGAGC 5008
BlkPLP1     CTCACAGCTTTTCTGAATCAAACCTATACCATTTCAACGATGGATTCTGAAATTGAGAGC5026
WhtPLP1     CTCACAGCTTTTCTGAATCAAACCTATACCATTTCAACGATGGATTCTGAAATTGAGAGC 5033
GOHPLP1     CTCACAGCTTTCCTGAATCAAACCTATACCATTTCAATGATGGATTCTGAAATGGAGAGC 5024
            *********** *********************** ************** ******

SumPLP1     CCGTAGCTTTAGGCTATTGTTCACACATTCCCAAGGACATTTTTTGGGCTGTGAAAAATC 5068
BlkPLP1     CTGTGGCTTTAGGCTATTTTTCACACATTCCCAAGGACATTTTTTGGGCTGTGAAAAATC 5086
WhtPLP1     CTGTGGCTTTAGGCTATTTTTCACACATTCCCAAGGACATTTTTTGGGCTGTGAAAAATC 5093
GOHPLP1     CTGTGGCTTTAGGCTATTGTTCACACATTTCCAAGGACATTTTTTGGGCTGTGAAAAATC 5084
            * ** ************ ********* ****************************

SumPLP1     AAAGTGAACCCAATGGACCTTTATCAGAATGTTTTGATTGTCAACTACTTTGATGAAGAC 5128
BlkPLP1     AAAGTGAACCCAATGGACCTTTCTCAGAATGTTTCGATTGTCAACTACTTAGATGAAGAC 5146
WhtPLP1     AAAGTGAACCCAATGGACCTTTCTCAGAATGTTTTGATTGTCAACTACTTAGATGAAGAC 5153
```

```
GOHPLP1     AAAGTGAACCCAATGGACCTTTCTCGGAATGTTTTGATTGTCAACTACTTTGATGAAGAC          5144
         ********************* ** ******** ************** *********

SumPLP1     ACAATTTCTTGCATGTTCTGAGTTTTTCCTAGTTGTAATCCAAAATTTGATTTTTTGCTT  5188
BlkPLP1     ACAATTTCTTGCATGTTCTGAGTTTTTCCTAGTTGTAATCCAAAATTTGATTTTTTGCTT  5206
WhtPLP1     ACAATTTCTTGCATGTTCTGAGTTTTTCCTAGTTGTAATCCAAAATTTGATTTTTTGCTT  5213
GOHPLP1     ACAGTTTCTTGCATGTTCTGAGTTTTTCCTAGTTGTAATCCAAAATTTGATTTTTTGCTT  5204
         *** ******************************************************

SumPLP1     GGACAGACTAACTGAGATTCTTCACTTCTGCTGAACTTAGATGCCCCCAGAACAAACGAA          5248
BlkPLP1     GGACAGACTAACTGAGATTCTTCACTTCTGCTGAACTTAGATGCCCCCAGAACAAAC---  5263
WhtPLP1     GGACAG----ACTGAGATTCTTCACTTCTGCTGAACTTAGATGCCCCCAGAACAAAC---  5266
GOHPLP1     GGACAGACTAACTGAGATTCTTCACTTCTGCTGAACTTAGATGCCCCCAGAACAAAC---5261
         ******    **********************************************

SumPLP1     TGAAAAAAGATTTCATTTCCATTGTGAAGAGTTGGACATCTGAAGCTTATCCTGTTATTG          5308
BlkPLP1     ---------ATTTCATTTCCATTGTGAAGAGTTGGACATCTGATGCTTATCCTGATATTG  5314
WhtPLP1     ---------ATTTCATTTCCATTGTGAAGAGTTGGACATCTGATGCTTATCCTGATATTG  5317
GOHPLP1     ---------ATTTCATTTCCATTGTGAAGAGTTGGACATCTGATGCTTATTCTGATATTG  5312
              ********************************* ****** *** *****
```

SumPLP1 TTGAACTGTTTATAGCTCAAGAGTGATAGATTTATCCAGGGCCATTTATTTCCAACTCCA 5368
BlkPLP1 TTGAACTGTTTATAGCTCAAGAGTGATAGATTCATCCAGGGCCATTTATTTCCAACTCCA 5374
WhtPLP1 TTGAACTGTTTATAGCTCAAGAGTGATAAATTCATTCAGGGCCATTTATTTCCAACTCCA 5377
GOHPLP1 TTGAACTGTTTATAGCTCAAGAGTGATAGATTCATCCAGGGCCATTTATTTCCAACTCCA 5372
*************************** *** ** ***********************

SumPLP1 TATTAGAGACTCATTAAAACCTATGTCCTTCTTGTCCCTTGCTTTGAGTGGCAGTGGAGC 5428
BlkPLP1 TATTAGAGACTCATTAAAGCCTATGTCCTTCTTGTCCCTTGCTTTGAGTGGCAGTGGAGC 5434
WhtPLP1 TATTAGAGACTCATTAAAGCCTATGTCCTTCTTGTCCCTTGCTTTGAGTGGCAGTGGAGC 5437
GOHPLP1 TATTAGAGACTCATTGAAGCCTATGTCCTTCTTGTCCCTTGCTTTGAGTGGCAGTGGAGC 5432
************** ** ******************************************

SumPLP1 AGAAAAGGGGGAGCTAAGTTATTTGAGTTCAAAGAAAATAGAGGAAAAATGGATGGAAAA 5488
BlkPLP1 AGAAAAGGGGGAGCTAAGTTATTTGAGCTCAAAGAAAATAGAGGAAAAATGGATGGAAAA 5494
WhtPLP1 AGAAAAGGGGGAGCTAAGTTATTTGAGCTCAAAGAAAATAGAGGAAAAATGGATGGAAAA 5497
GOHPLP1 AGAAAAGGGGGAGCTAAGTTATTTGAGCTCAAAGAAAATAGAGGAAAAGTGGATGGAAAA 5492
************************** ******************* ***********

SumPLP1 AGAAAGAGTATCTGTTGGTGATTCAGAGTTGCTCCCAAGGGTTAGCCCTTTATCCCCCAG 5548
BlkPLP1 AGAAAGAGTATCAGTTGGTGATTCAGAGTTGCTCCCAAGGCTTAGCCCTTTATCCGCCAG 5554
WhtPLP1 AGAAAGAGTATCAGTTCGTGATTCAGAGTTGCTCCCAAGGCTTAGCCCTTTATCCGCCAG 5557
GOHPLP1 AGAAAGAGTATCAGTTGGTGATTCAGAGTTGCTCCCAAGGCTTAGCCCTTTATCCCCCAG 5552
************ *** ********************** ************** ****

SumPLP1 TAAGAACACTACATATGACTCTCTGGTTATTTGGATGATGATCCAGGGTTCAGACATGCT 5608
BlkPLP1 TAAGAACACTACATATGACTCTCTGGTTATTTGGATGATGATCAAGGGATCAGACACGCT 5614
WhtPLP1 TAAGAACACTACATATGACTCTCTGGTTATTTGGATGATGATCAAGGGATCAGACACGCT 5617
GOHPLP1 TAAGAACACTACATATGACTCTCTGGTTATTTGGATGATGATGAAGGGATCAGACACGCT 5612
******************************************  **** ******* ***

SumPLP1 AAAGAGGATTTTTCCTCATCTTTACAACCCTGGAGGGGGCATCAGCTAAAAATTAGAGTG 5668
BlkPLP1 AAAGAGGATTTTTCCTCATCTTTACAACCCTGGAGGGAGCATCAGCTAAAAATTAGAGTG 5674
WhtPLP1 AAAGAGGATTTTTCCTCATCTTTACAACCCTGGAGGGAGCATCAGCTAAAAATTAGAGTG 5677

```
GOHPLP1    AAAGAGGATTTTTCCTCATCTTTACAAACCTGGAGGGAGCATCAGCTAAAAATTAGAGTG    5672
           *************************** ********* **********************

SumPLP1    GAATTTTTTGTTTGGTCAACAAGAGTTCAAGTTCCTCCATCCCTGATCACACCTGCACCA    5728
BlkPLP1    GAATTTTTTGTTTGGTCAGCAAGAGTTCAAGTTCCTCCATCCCTGATCACACCTGCACCA 5734
WhtPLP1    GAATTTTTTGTTTGGTCAGCAAGAGTTCAAGTTCCTCCATCCCTGATCACACCTGCACCA5737
GOHPLP1    GAATTTTTTGTTTGGTCAGCAAGAGTTCAAGTTCCTCCATCCCTGATCACACCTGCACCA    5732
           ****************** *****************************************

SumPLP1    CCATTTAGCCCTTCCCAAGGTGAAAAATGTGGGGTGAGGGGGCACAGGACGGTCACTTGC    5788
BlkPLP1    CCATTTAGCCCTTCTCAAGGTGAAAAAGCTGGGGTGAGGGGGCACAGGACGGTCACTTGC    5794
WhtPLP1    CCATTTAGCCCTTCCCAAGGTGAAAAAGCTGGGGTGAGGGGGCACAGGACGGTCACTTGC    5797
GOHPLP1    CCATTTAGCCCTTCCCAGGGTGAAAATGCTGGGGTGAGGGGGCACAGGACGGTCACTTGC    5792
           ************** ** ********   *******************************

SumPLP1    TAGGGTGTATGGCACTCTTTACTCTGGGCTAAACAAACAGTTCTGCCATTGTTCCCTGGG    5848
BlkPLP1    TAGGGTGTATGGCACTCTTTACTCTGGGCTAAACAAACAGTTCTGCCATTGTTCCCTGGG 5854
WhtPLP1    TAGGGTGTATGGCACTCTTTACTCTGGGCTAAACAAACAGTTCTGCCATTGTTCCCTGGG    5857
GOHPLP1    TAGGGTGTATGGCACTCTTTACTCTGGGCTAAACAAACAGTTCTGCCATTGTTCCCTGGG    5852
           ************************************************************
```

```
SumPLP1     TCTGACCCCTCCCCACCCCCAGGCCTGTGGGCCCTTGCAGAATACCAATGAGGCTCCCA     5908
BlkPLP1     TCTGACCCCTCCCCACCCCCAGGCCTGTGGGCCCCTGCAGAATACCAATGAGGCTCCCG     5914
WhtPLP1     TCTGACCCCTCCCCACCCCCAGGCCTGTGGGCCCCTGCAGAATACCAATGAGGCTCCCG     5917
GOHPLP1     TCTGACCCCTCCCCACCCCCAGGCCTGTGGGCCCCTGCAGAATACCAATGAGGCTCTCG     5912
            ********************************** ********************* *

SumPLP1     AGATCTCGGGGAAAAACAAGGCTTCTTTGTCCAAGGGAGCTATGGAGGGCTCTGTCCAAG    5968
BlkPLP1     AGATCTCAGGGAAAAACAAGGCTTCTTTGTCCAAGGGAGCTATGGAGGGCTCTGTCCAAG    5974
WhtPLP1     AGATCTCAGGGAAAAACAAGGCTTCTTTGTCCAAGGGAGCTATGGAGGGCTCTGTCCAAG    5977
GOHPLP1     AGATCTCAGGGAAAAACAAGGTTTCTTTGTCCAAGGGAGCTATGGAGGGCTCTGTCCAAG    5972
            ******* ************* **************************************

SumPLP1     CCCTGACCCTGTGCTGAGGAGAGAGGGAACAGTAGGGTCACTGACCTTACCCTCCCCCCA    6028
BlkPLP1     CCCTGACCCTGTGCTGGGGAGAGAGGGAACAGTGGGGTCACTGACCTTACCCTCCCCCCA    6034
WhtPLP1     CCCTGACCCTGTGCTGGGGAGAGAGGGAACAGTGGGGTCACTGACCTTACCCTCCCCCCA    6037
GOHPLP1     CCCTGACCCTGTGCTGGGGAGAGAGGGAACAGTGGGGTCACTGACCTTACCCTCCCCCCA    6032
            **************** **************** **************************

SumPLP1     CCCCCAGGCTTCAGAGACCTTCTTTACTAGAAGTGTCTAAGAGTTTGGGGGTGGGGAAGA    6088
BlkPLP1     CCCCCAGGCTTCAGAGACCTTCTTTACTAGAAGAGTCTAAGAGTTTGGGGGTGGGGAAGA    6094
WhtPLP1     CCCCCAGGCTTCAGAGACCTTCTTTACTAGAAGAGTCTAAGAGTTTGGGGGTGGGGAAGA    6097
GOHPLP1     CCCGCAGGCTTCAGAGATCTTCTTTACTAGAAGAGTCTAAGAGTTTGGGGGTGGGGAAGA    6092
            *** ************* *************** **************************

SumPLP1     GTTGGATAGGCGGAGAAGGAAAATGGCAGCACTGTTTATTTCCAAGGTCCTTTTCTTGCC    6148
BlkPLP1     GTTGGATAGGCGGAGAAGGAAAATGGCAGCACTGTTTATTTCCAAGCTCCTTTTCTTGAC    6154
WhtPLP1     GTTGGATAGGCGGGGAAGGAAAATGGCAGCACTGTTTATTTCCAAGCTCCTTTTCTTGAC    6157
GOHPLP1     GTTGGATAGGCAGAGAAGGAAAATGGCAGCACTGTTTATTTCCAAGCTCCTTTTCTTGAC    6152
            *********** * ******************************** *********** *

SumPLP1     TTACAGGATACATCTAGTATCTTAACCTGTTGAGAAAGAGGAAGATCAATTTTTGAGAAC    6208
BlkPLP1     TTACAGGATACATCTAGTATCTTAACCTGTTGAGAAAGAGAAAGATCAATTTTTGAGAAC    6214
WhtPLP1     TTACAGGATACATCTAGTATCTTAACCTGTTGAGAAAGAGGAAGATCAATTTTTGAGAAC    6217
```

```
GOHPLP1     TTACAGGATACATCTAGTATCTTAACCTGTTGAGAAAGAGGAAGATCAATTTTTGAGAAC        6212
            **************************************** *******************

SumPLP1     AACGATCAATGTCAACCCCTGTGTTTTTCTCTCTGGTATACATATCCGTGCATGTGCACA6268
BlkPLP1     AACGATCAATGTCAACCGCTGTGTTTTTCTCTCTGGTATACACATCCGTGCATGTGCACA 6274
WhtPLP1     AACGATCAATGTCAACCGCTGTGTTTTTCTCTCTGGTATACACATCCGTGCATGTGCACA6277
GOHPLP1     AACGATCAATGTCAACCGCTGTGTTTTTCTCTCTGGTATCCACATCCATGCATGTGCACA        6272
            **************** ******************** ** **** ************

SumPLP1     TTTGTGTGAATGCATGTGTGTGTGTGTGCATGTATGCGTCTGCATGCACGCACACGGGCG        6328
BlkPLP1     TTTGTGTGAATGCATGTGTGTGTGTGTGCATGTATGCGTCTACATGCACGCACGCGCGCG6334
WhtPLP1     TTTGTGTGAATGCATGTGTGTGTGTGTGCATGTATGCGTCTACATGCACGCACGCGCGCG        6337
GOHPLP1     TTTGTGTGAATGCATGTGTGTGTGTGTGCATGTATGCATCTGCATGCATGCACGCGCGCG        6332
            ************************************ *** ****** **** ** ***

SumPLP1     AG------------------CACACACACACACATCCCATGGGCCAGCCCTGATGAGGGA     6370
BlkPLP1     AGCACACACACACACACACACACACACACACACATCCCATGGGCCAGCCCTGATGAGGCA        6394
WhtPLP1     AG----------CACACACACACACACACACACATCCCATGGGCCAGCTCTGATGAGGCA        6387
GOHPLP1     AG------------------CACACACACACACATCCCATGGGCCAGCCCTGATGAGGCA     6374
            **       **************************** ********* *
```

```
SumPLP1    CTCTTACCAAGCACTATTGCAAAGTTCATACTAAGTAAACTCTGCTTCCCCTTACAACAG    6430
BlkPLP1    CTCTTACCAAGCACTATTGCAAAGTTCGTACTAAGTAAACTCTGCTTCCACTTACAACAG 6454
WhtPLP1    CTCTTACCAACCACTATTGCAAAGTTCATACTAAGTAAACTCTGCTTCCACTTACAACAG    6447
GOHPLP1    CTCTTACCAAGCACTACTGCAAAGTTCATACTAAGTAAACTCTGCTTCCACTTACAACAG    6434
           ********** ***** ********** ********************* **********

SumPLP1    AACAGCTACAAATTGATTGCTTCCCATAGAGCCCACGTTCTCTGACAGCAGTGCTTTACT    6490
BlkPLP1    AACAGCTACAAATTGATTGCTTCCCATAGAGGCCACGTTCTCTGACAGCAGTGCTTTAGT6514
WhtPLP1    AACAGCTACAAATTGATTGCTTCCCATAGAGGCCACGTTCTCTGACAGCAGTGCTTTAGT    6507
GOHPLP1    AACAGCTACAAATTGATTGCTTCCCATAAAGGCCACATTCTCTGACAGCAGTGCTTTAGT    6494
           **************************** ** **** ********************* *

SumPLP1    TCTTGGAAGAGGAAGGAAATTCAGTTCTCCTGCAAATGTTAGTGACTTTCTTTGCTTGGC    6550
BlkPLP1    TCTTGGAAGAGGAAGGAAATTCAGTTCTCCTGCAAATGTTAGTGACTTTCTTTGCTTGGC 6574
WhtPLP1    TCTTGGAAGAGGAAGGAAATTCAGTTCTCCTGCAAATGTTAGTGACTTTCTTTGCTTGGC    6567
GOHPLP1    TCTTGGAAGAGGAAGGAAATTCAGTTCTCCTGCAAATGTTAGTGACTTTCTTTGCTTGGC    6554
           ************************************************************

SumPLP1    TTGGACTTCTGAGCCTCACCTCTGGTGTGGCATACAAGAATCTCTGAGGACCAAGGAAGG    6610
BlkPLP1    TTGGACTTCTGAGCCTCAGCTCTGGTGTGGCATACAAGAATCTCTGAGGACCAAGGAAGG    6634
WhtPLP1    TTGGACTTCTGAGCCTCACCTCTGGTGTGGCA-ACAAGAATCTCTGAGGACCAAGGAAGG    6626
GOHPLP1    TTGGACTTCTGAGCCTCACCTCTGGTGTGGCATACAAGAATCTCTGAGGACCAAGGAAGG    6614
           ****************** ************* ***************************

SumPLP1    GCTCTCATTTTAGGGGACAGTGGGTAATAGGACTCACTTGTTCTGTGGCTCTGAGAACAG    6670
BlkPLP1    GCTATCATTTTAGGGGACAGTGGGTAATAGGACTCATTTGTTCTGTGGCTCTGAGAACAG    6694
WhtPLP1    GCTATCATTTTAGGGGACAGTGGGTAATAGGACTCACTTGTTCTGTGGCTCTGAGAACAG    6686
GOHPLP1    TCTATCATTTTAGGGGACAGTGGGTAATAGGACTCGTTTGTTCTGTGGCTCTGAGAACAG    6674
            ** ********************************  ***********************

SumPLP1    AGACAAATGACTTTACCTCAACTTTCCCATGATAGAGATAATTTTGTCCTTCAAAATCTG    6730
BlkPLP1    AGACAAATGCCTTTACCTCAACTTTCCCATGATAGAGATAATTTGGTCCTTCAAAATCTG 6754
WhtPLP1    AGACAAATGTCTTTACCTCAACTTTCCCATGATAGAGATAATTTGGTCCTTCAAAATCTG    6746
```

```
GOHPLP1     AGACAAATGCCTTTACCTCAACTTTCCCATGATAGACATAATTTTGTCCTTCAAAATCTG          6734
            ********* ************************* ******* ***************

SumPLP1     TTCTTATTGATGAAATTCAGC-------------CATGCAAATCACCAGATGTTCAAGCA 6777
BlkPLP1     TTCTTATTGATGAAATTCAGC-------------CATGCAAATCACCAGATGTTCAAGCA  6801
WhtPLP1     TTCTTATTGATGAAATTCAGC-------------CATGCAAATCACCAGATGTTCAAGCA 6793
GOHPLP1     TTCTTATTGATGAAATTCACCAGATGTTCTTATTGATGAAAATCACCAGATGTTCAAGCA          6794
            ****************** *    *** ********************

SumPLP1     CTTGGGGTAAAAAAAAAACCTGTACTAATATTAAGAAGGTCAGACAAAGGACTCCCCTAGT         6837
BlkPLP1     CTTGGGAT-AAAAAAAACCTGTACGAATATTAAGAAGGTCAGACAAAGGACTCCCCTAGT          6860
WhtPLP1     CTTGGGAT-AAAAAAAACCTGTACTAATATTAAGAAGGTCAGACAAAGGACTCCCCTAGT          6852
GOHPLP1     CTTGGGAT-AAAAAAAACCTGTACTAATATTAAGAAGGTCAGACAAAGGACTCCCCTAGT          6853
            ****** * ************** **********************************

SumPLP1     CCCTGGTACCCTGGTATAGACAGGTGAGCTTTGGATAAAAATCCAAACCTAAAGATTAAT          6897
BlkPLP1     CCCTGGTACCCTGGTATAAACAGGTGAGCTTTGGATAAAAATCCAAACCTAAAGATTAAT          6920
WhtPLP1     GCCTGGTACCCTGGTATAAACAGGTGAGCTTTGGATAAAAGTCCAAACCTAAAGATTAAT          6912
GOHPLP1     CCCTGGTACCCTGGTATAAACAGGTGAGCTTTGGATAAAAATCCAAACCTAAAGATTAAT          6913
             **************** ******************** ******************
```

```
SumPLP1     ACAATTTATTTTCTTTTTATAAGGAGCCCCAATGTACTTTTCCAAAATAGCAGCTGCTCA 6957
BlkPLP1     ACAATTTATTTTCTTTTTATAAGGAGCCCCAATGTGCTTTTCCAAAATAGCAGCTGCTCA 6980
WhtPLP1     ACAATTTATTTTCTTTTTATAAGGAGCCCCAATGTGCTTTTCCAAAACAGCAGCTGCTCA 6972
GOHPLP1     ACAATTTATTTTCTTTTTATAAGGAGGCCCAATGTGCTTTTCCAAAATAGCAGCTGCTCA 6973
            ************************* ******** *********** ************

SumPLP1     GTGCATTTGCTGATTATTTGTGTGTGTGTGTGTGTGTGTATACAAATAATTCATATAC 7017
BlkPLP1     GTGCATTTGCTGATTATT--TGTGTGTGTGTGTGTGTATATACAAATAATTCATATAC 7038
WhtPLP1     GTGCATTTGCTGATTATT------TGTGTGTGTGTGTATATACAAATAATTCATATAC 7026
GOHPLP1     CTGCATTTGCTGATTATT----------TTTGTGTGTGTATATACAAATAATTCATATAC 7023
             ***************** ********* *******************

SumPLP1     ACATATA--ATATATAAATACAAAAATAATTCATATATCATACCTATATATAGTATGATA 7075
BlkPLP1     ACATATAATATATATAAATACAAAAATAATTCGTATATCATATCTATATATAGTATGATA 7098
WhtPLP1     ACATATAATATATATAAATACAAAAATAATTCGTATATCATATCTATATATAGTATGATA 7086
GOHPLP1     ACATATAATATATATAAATACAAAAATAATTCATATATCATATCTATATATAGTATGATA 7083
            *******  ********************** ********* ****************

SumPLP1     TATGAATAGATGTATAAAATCAGTTATATGTGTATATGTATGTGTATTCTTTTCTGAAGA 7135
BlkPLP1     TATGAATAGATGTATAAAATCAGTTATATGTATATATGTATGTGTATTCTTTTCTGAAGA 7158
WhtPLP1     TATGAATAGATGTATAAAATCAGTTATATGTATATATGTATGTGTATTCTTTTCTGAAGA 7146
GOHPLP1     TATGAATAGATGTATAAAATCAGTTATATGTATATATGTATGTGTATTCTTTTCTGAAGA 7143
            ***************************** **************************

SumPLP1     GCTTAACATTTTCATTTTTTCCACATTTTGGGGATCTGGCCAAGAGGCCAGAGGGAGCCA 7195
BlkPLP1     GCTTAACATTTTCATTTCTTCCACATTTTGGGGATCTGGCCAAGAGGCCAGAGGGAGCCA 7218
WhtPLP1     GCTTAACATTTTCATTTCTTCCACATTTTGGGGATCTGGCCAAGAGGCCAGAGGGAGCCA 7206
GOHPLP1     GCTTAACATTTTCATTTCTTCCACATTTTGGGGATCTGGCCAAGAGGCCAGAGGGAGCCA 7203
            **************** *****************************************

SumPLP1     TCCTCTCCCCAACTAGTCAGTCATCTACTTGAAATTTGTTAGGTTTCACTTCATGCAGCA 7255
BlkPLP1     TCCTCTCCCCAACTAGTCAGTCATCTACTTGAAATTTGTTAGGTTTCACTCCATGCAGCA 7278
WhtPLP1     TCCTCTCCCCAACTAGTCAGTCATCTACTTGAAATTTGTTAGGTTTCACTCCATGCAGCA 7266
```

```
GOHPLP1   TCCTCTCCCCAACTAGTCAGTCATCTACTTGAAATTTGTTAGGTTTCACTCCATGCGGCA   7263
          ************************************************** ***** ***

SumPLP1   CCTTAGAGTGTGGGGCTGGTACTTGGGGCACAGCTGCTCTCAGAGCCTGTGACTTCTTGT   7315
BlkPLP1   CCTTAGAGTGTGGGGCTGGTACTTGGGGCACAGCTGCTCTCAGAGCCTGTGACTTCTTGT   7338
WhtPLP1   CCTTAGAGTGTGGGGCTGGTACTTGGGGCACAGCTGCTCTCAGAGCCTGTGACTTCTTGT   7326
GOHPLP1   CCTTAGAGTATGGGGCTGGTACTTGGGGCACAGCTGCTCTCAGAGCCTGTGACTTCTTGT   7323
          ********* **************************************************

SumPLP1   GTGCCCTTCCTGTTTCTCAGCAGCTTTGGCGTGGGGCCTGCAAGACTAATATCTCTGGCC   7375
BlkPLP1   GTGCCTTTCCTGTTTCTCAGCAGCTTTGGCGTGGGGCCT-CAAGACTAATATCTCTGGCC   7397
WhtPLP1   GTGCCTTTCCTGTTTCTCAGCAGCTTTGGCGTGGGGCCT-CAAGACTAATATCTCTGGCC   7385
GOHPLP1   GTGCCTTTCCTGTTTCTCAGCAGCTTTGGCGTGGGGCCTGCAAGACTAATATCTCTGGCC   7383
          ***** ********************************* ********************

SumPLP1   AGCACACATTCCTTCCTGGAACTCGAAGTGCATTTTCCCTATGTAACCACTTACTTGGCT   7435
BlkPLP1   AGCACACATTCCTTCCTGGAAGTCTAAGTGCATTTTCCCTATGTAACCACTTACTTGGCT   7457
WhtPLP1   AGCACACATTCCTTCCTGGAAGTCTAAGTGCATTTTCCCTATGTAACCACTTACTTGGCT   7445
GOHPLP1   AGCACACATTCCTTCCTGGAACTCTAAGTGCATTTTCCCTATGTAACCACTTACTTGGCT   7443
          ********************* ** ***********************************
```

SumPLP1 GCCTGGAATTGTTAGTTGAAAATATTAGGCCTGCCTTGCTCATTCTTCCCATATGGCAGA 7495
BlkPLP1 GCCTGGAATTGTTAGTTGAAAATACTAGGTCTGCCTTGCTCATTCTTCCCATATGGCAGA 7517
WhtPLP1 GCCTGGAATTGTTAGTTGAAAATACTAGGTCTGCCTTGCTCATTCTTCCCATATGGCAGA 7505
GOHPLP1 GCCTGGAACTGTTAGTTGAAAATACTAGGTCTACCTTGCTCATTCTTCCCATATGGCAGA 7503
******** *************** **** ** ***************************

SumPLP1 GTCTATGTCTTTTATGTTTTTACATCCCACATAACACCTAGCACAATGCTGGACACATAG 7555
BlkPLP1 GTCTATGTCTTTTATGTCTTTACATCCCACATAACACCTAGCACAATGCCGAACACATAG 7577
WhtPLP1 GTCTATGTCTTTTATGTCTTTACATCCCACATAACACCTAGCACAATGCTGGACACATAG7565
GOHPLP1 GTCTATGTCTTTTATGTCTTTACATCCCACATAACACCTAGCACAATGCGGGACACATAG 7563
***************** ******************************* * ********

SumPLP1 TAAGTACTCCATGAGAGTTGATGAATGACTAACACAGACAAATCAGAATGATAAGAAAGG 7615
BlkPLP1 TAAGTACTCCATGACAGTTGATAAATGACTAACACAGACAAATCAGAATGATAAGAAAGG 7637
WhtPLP1 TAAGTACTCCATGACAGTTGATAAATGACTAACACAGACAAATCAGAATGATAAGAAAGG 7625
GOHPLP1 TAAGTACTCCATGAGAGTTGATGAATGACTAACACAGACAAATCAGAATGATAAGAAAGG 7623
************** ******* *************************************

SumPLP1 AGAGAATGATTACATAATCTCTCTAGCTTGCTACTTGAATTACTGATAATTTAAAAAAA 7675
BlkPLP1 AGAGAATGATTACATAATCTCTCTAGCTTGCTACTTGAATTACTGATAATTTAAAAAAA 7697
WhtPLP1 AGAGAATGATTACATAATCTCTCTAGCTTGCTACTTGAATTACTGATAATTTAAAAAAA 7685
GOHPLP1 AGAGAATGATTACATAATCTCTCTAGCTTGCTACTTGAATTACTGATAATTTAAAAAAAA 7683
*************************************************** *******

SumPLP1 CATAAATTCTGGGAATTTAAGGTAAAGATTTATTTTCAAGAATGGAACAAAGAATGCTTT 7735
BlkPLP1 CATAAATTCTGGGAATTTAAGGTAAAGATTTATTTTCAAGAATGGAACAAAGAATGCTTT 7757
WhtPLP1 CATAAATTCTGGGAATTTAAGGTAAAGATTTATTTTCAAGAATGGAACAAAGAATGCTTT 7745
GOHPLP1 CACAAATTCTGGGAATTTAAGGTAAAGATTTATTTTCAAGAATGGAACAAAGAATGCTTT 7743
** *********************************************************

SumPLP1 TGGGGTCAATCTCACATTCCAGAAGCCTAAAGCTCTAGTCTCTCCTGCTTTTGAACTCAG 7795
BlkPLP1 TGGGGTCAATCTCACATTCCAGAAGCCTAAAGCTCTAGTCTCTCCTGCTTTTGAACTCAG 7817
WhtPLP1 TGGGGTCAATCTCACATTCCAGAAGCCTAAAGCTCTAGTCTCTCCTGCTTTTGAACTCAG 7805

```
GOHPLP1    TGGGGTCAATCTCACATTCCAGAAGCCTAAAGCTCTAGTCTCTCCTGCTTTTGAACTCAG    7803
           ************************************************************

SumPLP1    GAGTGACCGCTCTATCCATGCATGCGAGCACAGGGTCTGACTGGTCTGGCAGAGAGGTAT    7855
BlkPLP1    GAGTGACCGCTCTATCCGTGCATGCGAGCACAGGATCTGACTGGTCTGGCAGAGAGGTAT    7877
WhtPLP1    CAGTGACCGCTCTATCCGTGCATGCGAGCACAGGATCTGACTGGTCTGGCAGAGAGGTAT    7865
GOHPLP1    GAGTGACCGCTCTATCTGTGCATGCGAGCACAGGATCTGACTGGTCTGGCAGAGAGGTAT    7863
            ***************  **************** *************************

SumPLP1    GAGTGGCACAAACTACCTATTGTACTTCCCTGACTGTTTCCCCTCCTTCTTTCTCAGGCT 7915
BlkPLP1    GAGTGGCACAAACCACCTATTGTAGTTCCCTGACTGTTTCCCCTCCTTCTTCCTCAGGCT 7937
WhtPLP1    GAGTGGCACAAACTACCTATTGTAGTTCCCTGACTGTTTCCCCTCCTTCTTCCTCAGGCT 7925
GOHPLP1    GAGTGGCACAAGCTACCTATTGTACTTCCCTGACTGTTTCCCCTCCTTCTTCCTCAGGCT    7923
           *********** * ********** ************************** ********

SumPLP1    TGTTAGAGTGCTGTGCAAGATGTCTTGTAGGGGCCCCCTTTGCTTCCCTGGTGGCCACTG    7975
BlkPLP1    TGTTAGAGTGCTGTGCAAGATGTCTTGTAGGGGCCCCCTTTGCTTCCCTGGTGGCCACTG 7997
WhtPLP1    TGTTAGAGTGCTGTGCAAGATGTCTTGTAGGGGCCCCCTTTGCTTCCCTGGTGGCCACTG    7985
GOHPLP1    TGTTAGAGTGCTGTGCAAGATGTCTTGTAGGGGCCCCCTTTGCTTCCCTGGTGGCCACTG    7983
           ************************************************************
```

SumPLP1 GATTGTGTTTCTTTGGGGTGGCACTGTTCTGTGGCTGTGGACATGAAGCACTCACTGGCA 8035
BlkPLP1 GATTGTGTTTCTTTGGGGTGGCACTGTTCTGTGGCTGTGGACATGAAGCACTCACTGGCA 8057
WhtPLP1 GATTGTGTTTCTTTGGGGTGGCACTGTTCTGTGGCTGTGGACATGAAGCACTCACTGGCA 8045
GOHPLP1 GATTGTGTTTCTTTGGGGTGGCACTGTTCTGTGGCTGTGGACATGAAGCACTCACTGGCA 8043
************************************************************

SumPLP1 CAGAAAAGCTAATTGAGACCTATTTCTCCAAAAACTACCAGGACTATGAGTATCTCATCA 8095
BlkPLP1 CAGAAAAGCTAATTGAGACCTATTTCTCCAAAAACTACCAGGACTATGAGTATCTCATCA 8117
WhtPLP1 CAGAAAAGCTAATTGAGACCTATTTCTCCAAAAACTACCAGGACTATGAGTATCTCATCA 8105
GOHPLP1 CAGAAAAGCTAATTGAGACCTATTTCTCCAAAAACTACCAGGACTATGAGTATCTCATCA 8103
************************************************************

SumPLP1 ATGTGTAAGTACCTGTCGTCTCATACAAACCCCTCTCTTTCCCTCTCTCCATCCTGAAGA 8155
BlkPLP1 ATGTGTAAGTACCTGTCGTCTCATACAAACCCCTCTCTTTCCCTCTCTCCATCCTGAAGA 8177
WhtPLP1 ATGTGTAAGTACCTGTCGTCTCATACAAACCCCTCTCTTTCCCTCTCTCCATCCTGAAGA 8165
GOHPLP1 ATGTGTAAGTACCTGTCGTCTCATACAAATCCTTCTCTTTCCCTCTCTCCATCCTGAAGA 8163
***************************** ** ***************************

SumPLP1 TACAGAACTCATCAGTACCTTAGTGACTAGTAGGAGATTGGTGTGGAGCCAGATAGGATT 8215
BlkPLP1 TACAGAACTCATCAGTACCTTAGTGACTAGTAGGAGATTGGTGTGGAGCCAGATAGGATT 8237
WhtPLP1 TACAGAACTCATCAGTACCTTAGTGACTAGTAGGAGATTGGTGTGGAGCCAGATAGGATT 8225
GOHPLP1 TACAGAACTCATCAGTACCTTAGTGACTAGTAGGAGATTGGTGTGGAGCCAGATAGGATT 8223
************************************************************

SumPLP1 CCTGAGTCTTCCCTCTGTGCGCAGATTCCACCTATGCCACATCGGAAGCCCTAAAGGAAG 8275
BlkPLP1 CCTGAGTCTTCCCTCTGTGTGCAGATTCCACCTATGCCACATCTGAAGCCCTAAATGAAG 8297
WhtPLP1 CCTGAGTCTTCCCTCTGTGTGCAGATTGCACCTATGCCACATCTGAAGCCCTAAATGAAG 8285
GOHPLP1 CCTGAGTCTTCCCTCTGTGTGCAGATTCCACCTATGCCACATCTGAAGCCCTAAATGAAG 8283
******************* ******* *************** *********** ****

SumPLP1 GAAAAGTGCCAAATTCAGAAAGGGGAGGTTTTCCTGCCTGCCAGCCCACTCTCATCCCTC 8335
BlkPLP1 GAAAAGTGCCAAATTCAGAAAGGGGAGATTTTCCTGCCTGCCAGCCCACTCTCATCACTT 8357
WhtPLP1 GAAAAGTGCCAAATTCAGAAAGGGGAGGTTTTCCTGCCTGCCAGCCCACTCTCATCACTC 8345

```
GOHPLP1     GAAAAGTGCCAAATTCAGAAAGGGGAGGTTTTCCTGCCTGCCAACCCACTCTCATCACTC     8343
            *************************** *************** ************ **

SumPLP1     CCCCTTACCCACCAGTGCCCTTGATTTGTCCCACAGGTTCTAACTTCTGCTTGGTAGGGT 8395
BlkPLP1     CCCCTCACCCCCCACTGCCCTTGCTTTGTCCCACAGGTTCTAACTTCTGCTTGGTAGGGT 8417
WhtPLP1     CCCCTCACCCCCCACTGCCCTTGCTTTGTCCCACAGGTTCTAACTTCTGCTTGGTAGGGT 8405
GOHPLP1     CCTCTCACCCCCCACTGCCCTTGCTTTGTCCCACAGGTTCTAACTTCTGCTTGGTAAGGT     8403
            ** ** **** *** ******** ******************************** ***

SumPLP1     TAACAATGATCGAGTTGAAGCCCCTGTGGTGGTAGGTATGGAGAACTCAGGGGAGGCACA     8455
BlkPLP1     TAACAATGATCGAGTTGAAGCCCCTGTGGTGGTAGGTATGGAGAACTCAGGGGAGGCACA     8477
WhtPLP1     TAACAATGATCGAGTTGAAGCCCCTGTGGTGGTAGGTATGGAGAACTCAGGGGAGGCACA     8465
GOHPLP1     TAATAATGATCGAGTTGAAGCCCCTGTGGTGATAGGTATGGAGAACTCAGGGGAGGCACA     8463
            *** *************************** ****************************

SumPLP1     ATGATGGGACAGAGTGGAGTTGCTGCACTTGGTTTGACACACTGCTTGAGTCTGCTCAGG     8515
BlkPLP1     ATGATGGGACAGAGTGGAGTTGCTGCACTTGGTTTGACACACTGCTTGAGTCTGCTCAGG     8537
WhtPLP1     ATGATGGGACAGAGTGGAGTTGCTGCACTTGGTTTGACACACTGCTTGAGTCTGCTCAGG     8525
GOHPLP1     ATGATGGGACAGAGTGGAGTTGCTGCACTTTGTTTGACACACTGCTTGAGTCTGCTCAGG     8523
            ****************************** *****************************
```

SumPLP1 CAGTACTAAGCCTGTCCACCTAGAGCAAGTTAAACTCCCATTCCTTCATCTCTCCTCTTT 8575
BlkPLP1 CAGTACTAAGCCTGTCCTCCTAGAGCAAGTTAAACTCCCATTCCTTCATCTCTCCTCTTT 8597
WhtPLP1 CAGTACTAAGCCTGTCCTCCTAGAGCAAGTTAAACTCCCATTCCTTCATCTCTCCTCTTT 8585
GOHPLP1 CAGTACTAAGCCTGTCCTCCTAGAGCAAGTTAAACTCCCATTCCTTCATCTCTCCTCTTT 8583
***************** ******************************************

SumPLP1 CTTGTCTAGCTTCTCTCTCCTCTCTCCTCTTAGGGAGCGTCTCTGAAGGGTCAGGCCAAG 8635
BlkPLP1 CTTGTCTAGCTTCTCTCTCCTCTCTCTTCTTAGGGAGCGTCTCTGAAGGGTCAGGCCAAG 8657
WhtPLP1 CTTGTCTAGCTTCTCTCTCCTCTCTCTTCTTAGGGAGCGTCTCTGAAGGGTCAGGCCAAG 8645
GOHPLP1 CTTGTCTAGCTTCTCTCTCCTCTCTCCTCTTAGGGAGCATCTCTGAAGGGTCAGGCCAAG 8643
************************** *********** *********************

SumPLP1 GAGAGTAGGGCCCCGGTTCTTCAAGTCACAGAGTAAGCAAGGTGTGGGTGGAAGAGCATG 8695
BlkPLP1 GAGAGTAGGGCCCCGGTTCTTCAAGTCACAGAGTAAGCAAGGTGTGGGTGGAAGAGCATG 8717
WhtPLP1 GAGAGTAGGGCCCTGGTTCTTCAAGTCACAGAGTAAGCAAGGTGTGGGTGGAAGAGCATG 8705
GOHPLP1 GAGAGTAGGGCCCCGGTTCTTCAAGTCACAGAGTAAGCAAGGTGTGGGTGGAAGAGCATG 8703
************* **********************************************

SumPLP1 TGCTGCTGGCCACATGCTCCCTGTCTTCCTTTACTTCAATAAAGATAAGGTGGAAGCATG 8755
BlkPLP1 TGCTGCTGGCCATGTGCTCCCTGTCTTCCTTTACTTCAATAAAGATAAGGTGGAAGCATG 8777
WhtPLP1 TGCTGCTGGCCATGTGCTCCCTGTCGTCCTTTACTTCAATAAAGATAAGGTGGAAGCATG 8765
GOHPLP1 TGCTGCTGGCCACATGCTCCCTGTCTTCCTTTACTTCAATAAAGATAAGGTGGAAGCATG 8763
************  *********** **********************************

SumPLP1 GAGGTGGAACCTAGTGATGCCTTTCAAGATTCCCTAGCCTTGTTTAGGTGCCCACTCAAG 8815
BlkPLP1 GAGGTGGAACCTAGTGATGCCTTTCAGGAATCCCTAGCCTTGTTTAGGTGCCCACTCAAG 8837
WhtPLP1 GAGGTGGAACCTAGTGATGCCTTTCAGGAATCCTTAGCCTTGTTTAGGTGCCCACTCAAG 8825
GOHPLP1 AAGGTGGAACCTAGTGATGCCTTTCAAGAATCCCTGGCCTCGTTTAGGTGCCCACTCAAG 8823
 ************************* ** *** * **** *******************

SumPLP1 TGTCTACCTTACTTATGCAGGAAAGGAACTGGGCCCTCAATTAGTAAGATTCTCTGGTCT 8875
BlkPLP1 TGTCTACCTTACTTATGCAGGAAAGGAACTGGGCCCTCAATTAGTAAGACTCTCTGGTCT 8897
WhtPLP1 TGTCTACCTTACTTATGCAGGAAAGGAACTGGGCCCTCAATTAGTAAGATTCTCTGGTCT 8885

```
GOHPLP1    TGTCTATCTTACTTATGCAGGAAAGGAACTGGGCCCTCAATTAGTAAGATTCTCTGGTCT    8883
           ****** ****************************************** **********

SumPLP1    GTTTTGTCTATCTGTTAATGCAGGATCCATGCCTTCCAGTATGTCATCTATGGAACTGCC 8935
BlkPLP1    GTTTTGTGTATCTGTTAATGCAGGATCCATGCCTTCCAGTATGTCATCTATGGAACTGCC 8957
WhtPLP1    GTTTTGTCTATCTGTTAATGCAGGATCCATGCCTTCCAGTATGTCATCTATGGAACTGCC 8945
GOHPLP1    GTTTTGTCTATCTGTTAATGCAGGATCCATGCCTTCCAGTATGTCATCTATGGAACTGCC    8943
           ******* ****************************************************

SumPLP1    TCTTTCTTCTTCCTTTATGGGGCCCTCCTGCTGGCTGAGGGCTTCTACACCACCGGCGCA 8995
BlkPLP1    TCTTTCTTCTTCCTTTATGGGGCCCTCCTGCTGGCTGAGGGCTTCTACACCACCGGCGCA 9017
WhtPLP1    TCTTTCTTCTTCCTTTATGGGGCCCTCCTGCTGGCTGAGGGCTTCTACACCACCGGCGCA 9005
GOHPLP1    TCTTTCTTCTTCCTTTATGGGGCCCTCCTGCTGGCTGAGGGCTTCTACACCACCGGCGCA    9003
           ************************************************************

SumPLP1    GTCAGGCAGATCTTTGGCGACTACAAGACCACCATCTGCGGCAAGGGCCTGAGCGCAACG    9055
BlkPLP1    GTCAGGCAGATCTTTGGCGACTACAAGACCACCATCTGCGGCAAGGGCCTGAGCGCAACG    9077
WhtPLP1    GTCAGGCAGATCTTTGGCGACTACAAGACCACCATCTGCGGCAAGGGCCTGAGCGCAACG    9065
GOHPLP1    GTCAGGCAGATCTTTGGCGACTACAAGACCACCATCTGCGGCAAGGGCCTGAGCGCAACG    9063
           ************************************************************
```

SumPLP1 GTAACAGGGGGCCAGAAGGGGAGGGGTTCCAGAGGCCAACATCAAGCTCATTCTTTGGAG 9115
BlkPLP1 GTAACAGGGGGCCAGAAGGGGAGGGGTTCCAGAGGCCAACATCAAGCTCATTCTTTGGAG 9137
WhtPLP1 GTAACAGGGGGCCAGAAGGGGAGGGGTTCCAGAGGCCAACATCAAGCTCATTCTTTGGAG 9125
GOHPLP1 GTAACAGGGGGCCAGAAGGGGAGGGGTTCCAGAGGCCAACATCAAGCTCATTCTTTGGAG 9123
************************************************************

SumPLP1 CGGGTGTGTCATTGTTTGGGAAAATGGCTAGGACATCCCGACAAGGTGATCATCCTCAGG 9175
BlkPLP1 CGGGTGTGTCATTGTTTGGGAAAATGGCTAGGACATCCCGACAAGGTGATCATCCTCAGG 9197
WhtPLP1 CGGGTGTGTCATTGTTTGGGAAAATGGCTAGGACATCCCGACAAGGTGATCATCCTCAGG 9185
GOHPLP1 CGGGTGTGTCATTGTTTGGGAAAATGGCTAGGACATCCCGACAAGGTGATCATCCTCAGG 9183
************************************************************

SumPLP1 ATTTTGTGGCAATAACAAGGGGTGGGGGACAATTGGGCGTGAGTCTGTGGCCTCACCTCC 9235
BlkPLP1 ATTTTGTGGCAATAACAAGGGGTGGGGGACAATTGGGCGTGAGTCTGTGGCCTCACCTCC 9257
WhtPLP1 ATTTTGTGGCAATAACAAGGGGTGGGGGACAAATGGGCGTGAGTCTGTGGCCTCACCTCC 9245
GOHPLP1 ATTTTGTGGCAATAACAAGGGGTGGGGGACAATTGGGCGTGAGTCTGTGGCCTCACCTCC 9243
********************************* **************************

SumPLP1 ACTCAAGGCTGGGTCCTCTCTAGGGACCTGGCTTTTGAGTGAGGAAGTGATGGCTACAGC 9295
BlkPLP1 ACTCAAGGCTGGGTCCTCTCTAGGGGCCTGGCTTTTGAGTGAGGAAGTGATGGCTACAGC 9317
WhtPLP1 ACTCAAGGCTGGGTCCTCTCTAGGGGCCTGGCTTTTGAGTGAGGAAGTGATGGCTACAGC 9305
GOHPLP1 ACTCAAGGCTGGGTCCTCTCTAGGGGCCTGGCTTTTGAGTGAGGAAGTGATGGCTACAGC 9303
************************* **********************************

SumPLP1 CGAACGAGAAGGTCAGGAAGAACGTGGTGCCCAGCTGGCTTAGCCTCACCTTTCAAAAGT 9355
BlkPLP1 CGAACGAGAAGGTCAGGAAGAACGTGGTGCCCAGCTGGCTTAGCCTCACCTTTCAAAAGT 9377
WhtPLP1 AGAACGAGAAGGTCAGGAAGAACGTGGTGCCCAGCTGGCTTAGCCTCACCTTTCAAAAGT 9365
GOHPLP1 CGAACGAGAAAGTCAGGAAGAACGTGGTGCCCAGCTGGCTTAGCCTCACCTTTCAAAAGT 9363
 ********* *************************************************

SumPLP1 TCCCTAGAAAATTTCGTCTCAAAGTTAAAAGCATGAGTTTTATGAGATTCTTTGTATAAT 9415
BlkPLP1 TCCCTAGAAAATTTCA-----AAGTTAAAAGCATGAGTTTTATGAGATTCTTTGTATAAT 9432
WhtPLP1 TCCCTAGAAAATTTCATCTCAAAGTTAAAAGCATGAGTTTTATGAGATTCTTTGTATAAT9425

```
GOHPLP1     TCCCTAGAAAATTTCATCTCGAAGTTAAAAGCATGAGTTTTATGAGATTCTTTGTATAAT          9423
            ***************   ****************************************

SumPLP1     CAGACCATTTCTAAGTCATCTGTTGGTATGCCTTTATTCCCCTCCTAGCAAGGACCACAA          9475
BlkPLP1     CAGACCATTTCTAAGTCATCTGTTGGTATGCCTTTATTCCCCTCCTAGCAAGGACCACAA 9492
WhtPLP1     CAGACCATTTCTAAGTCATCTGTTGGTATGCCTTTATTCCCCTCCTAGCAAGGACCACAA9485
GOHPLP1     CAGACCATTTCTAAGTCATCTGTTGGTATGCCTTTATTCCCCTCCTAGCAAGGACCACAA          9483
            ************************************************************

SumPLP1     GAGTAGCTCTGATTGCCCAAGAGTCTAAAATGCTTCTCATGGAGTTGAGACACAGGTACC          9535
BlkPLP1     GAGTAGCTCTGATTGCCCAAGAGTCTAAAATGCTTCTCCTGGAGTTGAGACACAGGTACC          9552
WhtPLP1     GAGTAGCTCTGATTGCCCAAGAGTCTAAAATGCTTCTCCTGGAGTTGAGACACAGGTACC          9545
GOHPLP1     GAGTAGCTCTGATTGCCCAAGAGTCTAAAATGCTTCTCATGGAGTTGAGACACAGGTACC          9543
            ************************************** *********************

SumPLP1     TGATCTGAGAGGGAGGGTGGATCATGGAAATTAAATAATTGACTCCAAGGTAGAATTCAC          9595
BlkPLP1     TGATCTGAGAGGGAGGGTGGATCATGGAAATTAAATAATTGACTCCAAGGTGGAATTCAC          9612
WhtPLP1     TGATCTGAGAGGGAGGGTGGATCATGGAAATTAAATAATTGACTCCAAGGTGGAATTCAC          9605
GOHPLP1     TGATCTGAGAGGGAGGGTGGATCATGGAAATTAAATAATTGACTCCAAGGTGGAATTCAC          9603
            *************************************************** ********
```

SumPLP1 AACATTCTGGGATTCTACACTGTTAGAGATCCCAGAGAGAACTAGTGTAAATCTGCATAT 9655
BlkPLP1 AATATTCTGGGATTCTACACTGTTAGAGATCCCAGAGAGAACTAGTGTAAATCTGCATAT 9672
WhtPLP1 AATTTTCTGGGATTCTACACTGTTAGAGATCCCGGAGAGAACTAGTGTAAATCTGCATAT 9665
GOHPLP1 AATATTCTGGGATTCT--ACTGTTAGAGATCCCAGAGAGAACTAGTGTAAATCTGCATAT 9661
** ************ *************** **************************

SumPLP1 TTAATGCTTCATCTTTGTCTGAGGGCTATCCATGCCTCTAGTGAGGCACACACTGGTTTT 9715
BlkPLP1 TTAATGCTTCATCTTTGTCTGAGGGCTATCCATGCCTCTAGTGAGGCACACACTGGTTTT 9732
WhtPLP1 TTAATGCTTCATCTTTGTCTGAGGGATATCCATGCCTCTAGTGAGGCACACACTGGTTTT 9725
GOHPLP1 TTAGTGCTTCATCTTTGTCTGAGGGCTATCCACGCCTCTAGTGAGGCACACACTGGTTTT 9721
*** ********************* ****** ***************************

SumPLP1 CAGTCTCCAGATTTAAAGATCTTTGGGGGCTGAACATTTGTGGGTTGTGATGATGTCTTT 9775
BlkPLP1 CAGTCTCCAGAGTTAAAGATCTTTGGGGGCTGAACATTTGTGGGTTGTGATGATGTCTTT 9792
WhtPLP1 CAGTCTCCAGAGTTAAAGATCTTTGGGGGCTGAACATTTGTGGGTTGTGATGATGTCTTT 9785
GOHPLP1 CAGTCTCCAGAGTTAAAGATCTTTGGGGGCTGAACATTTGTGGGTTGTGATGATGTCTTT 9781
*********** ************************************************

SumPLP1 TGTGGCCAGCAGTTGGCTTAGAAAGGTTTTTGGATCTGAAATTGAAATGTCCTATTGGCT 9835
BlkPLP1 TGTAGCCAGCAGTTGGCTTAGAAAGGTTTTTGGATCTGAAATTGAAATGTCCTATTGGCT 9852
WhtPLP1 TGTGGCCAGCAGTTGGCTTAGAAAGGTTTTTGGATGTGAAATTGAAATGTCCTATTGGCT 9845
GOHPLP1 TGTGGTCAGCAGTTGGCTTAGAAAGGTTTTTGGATCTGAAATTGAAATGTCCTATTGGTT 9841
*** * ****************************** ********************** *

SumPLP1 TTGTTCAATGACTAGGGATCAAAAGTCCTGTGACAGGGAACATTTTCTAAGCTCAGCCTA 9895
BlkPLP1 TTGTTCAATGACTAGAGATCAAAAGTCCTGTGACAGGGAACATTTTCTAAGCTCAGCCTA 9912
WhtPLP1 TTGTTCAATGACTAGAGATCAAAAGTCCTGTGACAGGGAACATTTTCTAAGCTCAGCCTA 9905
GOHPLP1 TTGTTCAATGACTAGGGATCAAAAGTCCTGTGACAGGGAACATTTTCTAAGCTCAGCCTA 9901
*************** ********************************************

SumPLP1 AGGCAAGAAATGGCACGACTTTCTTTCAAGATCTGTTTTGATTTCTTCACATCTTATCTG 9955
BlkPLP1 AGGCAATAAATGGCACGACTTTCTTTCAAGATCTGTTTTGATTTCTTCACATCTTATCTG 9972
WhtPLP1 AGGCAAGAAATGGCACGACTTTCTTTCAAGATCTGTTTTGATTTCTTCACATCTTATCTG 9965

```
GOHPLP1     AGGCAAGAAATGGCACGACTTTCTTTCAAGATCTGTTTTGATTTCTTCACATCTTATCTG          9961
            ****** *******************************************************

SumPLP1     CCCAAAAAATCTCCTGAAGCCTCTCTAACCCAGGGATCCTCCTCACTCCAACCCCTACCC         10015
BlkPLP1     CCCAAAAAATCTCCTGAAGCCTCTCTAACCCAGGGATCCTCCTCACTCCAACCCCTACCC 10032
WhtPLP1     CCCAAAAAATCTCCTGAAGCCTCTCTAACCCAGGGATCCTCCTCACTCCAACCCCTACCC         10025
GOHPLP1     CCCAAAAAATCTCCTGAAGCCTCTCTAACCCAGGGATCCTCCTCACTCCAACCCCTACCC         10021
            ************************************************************

SumPLP1     ATTCCCCCCACCCTCTGTCACACTTGGGGCCAGTTCTCTAGTAGATACTGCCAATGACAT         10075
BlkPLP1     ATTCCCCCCACCCTCTGTCACACTTGGGGCCAGTTCTCTAGTAGATACTGCCAATGACAT 10092
WhtPLP1     ATTCCCCCCACCCTCTGTCACACTTGGGGCCAGTTCTCTAGTAGATACTGCCAATGACAT         10085
GOHPLP1     ATTCCCCCCACCCTCTGTCACACTTGGGGCCAGTTCTCTAGTAGATACTGCCAATGACAT         10081
            ************************************************************

SumPLP1     TTGGCAAATATGCCCTGCTTACTAATTTCATTTGGTGGAAATTCCTGGAATCTGGTTTCA 10135
BlkPLP1     TTGGCAAATATGCCCTGCTTACTAATTTCATTTGGTGGAAATTCCTGGAATCTGGTTTCA 10152
WhtPLP1     TTGGCAAATATGCCCTGCTTACTAATTTCATTTGGTGGAAATTCCTGGAATCTGGTTTCA 10145
GOHPLP1     TTGGCAAATATGCCCTGCTTACTAATTTCATTTGGTGGAAATTCCTGGAATCTGGTTTCA         10141
            ************************************************************
```

SumPLP1 TGTCTGGCACATGTCACTCCAGGATCTCCCAGTTTGTGTTTCAATGTCTGCAGGCTGATG 10195
BlkPLP1 TGTCTGGCACACGTCACACCAGGATCTCCCAGTTTGTGTTTCAACGTCTGCAGGCTGATC 10212
WhtPLP1 TGTCTGGCACATGTCACTCCAGGATCTCCCAGTTTGTGTTTCAATGTCTGCAGGCTGATC 10205
GOHPLP1 TGTCTGGCACATGTCACTCCAGGATCTCCCAGTTTGTGTTTCAACGTCTGCAGGCTGATG 10201
*********** ***** ************************* **************

SumPLP1 CTGATTTCTAACCACCCCATGTCAATTGTTTTAGTTTGTGGGCATCACCTATGCCCTGAC 10255
BlkPLP1 CTGATTTCTAACCACCCCGTGTCAATTGTTTTAGTTTGTGGGCATCACCTATGCCCTGAC 10272
WhtPLP1 CTGATTTCTAACCACCCCGTGTCAATTGTTTTAGTTTGTGGGCATCACCTATGCCCTGAC 10265
GOHPLP1 CTGATTTCTAACCACCCCATGTCAATTGTTTTAGTTTGTGGGCATCACCTATGCCCTGAC 10261
****************** *****************************************

SumPLP1 CATTGTGTGGCTCCTGGTTTTTGCCTGCTCTGCTGTGCCTGTGTACATTTACTTCAACAC 10315
BlkPLP1 CATTGTGTGGCTCCTGGTTTTTGCCTGCTCTGCTGTGCCTGTGTACATTTACTTCAACAC 10332
WhtPLP1 CATTGTGTGGCTCCTGGTTTTTGCCTGCTCTGCTGTGCCTGTGTACATTTACTTCAACAC 10325
GOHPLP1 CATTGTGTGGCTCCTGGTTTTTGCCTGCTCTGCTGTGCCTGTGTACATTTACTTCAACAC 10321
************************************************************

SumPLP1 GTGGACCACCTGCCAGTCGATTGCCTTCCCCAGCAAGACCTCTGCCAGTATAGGCAGTCT 10375
BlkPLP1 GTGGACCACCTGCCAGTCTATTGCCTTCCCCAGCAAGACCTCTGCCAGTATAGGCAGTCT 10392
WhtPLP1 GTGGACCACCTGCCAGTCTATTGCCTTCCCCAGCAAGACCTCTGCCAGTATAGGCAGTCT 10385
GOHPLP1 GTGGACCACCTGCCAGTCTATTGCCTTCCCAAGCAAGACCTCCGCCAGTATAGGCACTCT 10381
****************** *********** *********** ************ ***

SumPLP1 CTGTGCTGATGCCAGAATGTATGGTGAGTTAGGGCATACGTGCCTTGGCTCCCCTACCCC 10435
BlkPLP1 CTGTGCTGATGCCAGAATGTATGGTGAGTTAGGGCATGGGTGCCTTGGCTCCCCTACCCC 10452
WhtPLP1 CTGTGCTGATGCCAGAATGTATGGTGAGTTAGGGCATGGGTGCCTTGGCTCCCCTACCCC 10445
GOHPLP1 CTGTGCTGATGCCAGAATGTATGGTGAGTTAGGGCATGGGTGCCTTGGCTCCCCTACCCC 10441
*************************************  *********************

SumPLP1 CTATGGAAGCACTGTATATTTGGTTCTTTACTTAGGGTAAGGAGGGTGGTAATTATGGGA 10495
BlkPLP1 CTATGGAAGCACTGTATATTTGGTTCTTTACTTAGGGTAAGGAGGGTGGTAATTATGGGA 10512
WhtPLP1 CTATGGAAGCACTGTATATTTGGTTCTTTACTTAGGGTAAGGAGGGTGGTAATTATGGGA 10505

```
GOHPLP1     CTATGGAAGCACTGTATATTTGGTTCTTTACTTAGGGTAAGGAGGGTGGTAATTATGGGA     10501
            ************************************************************

SumPLP1     AAAAATATTATGGATGCCTGGATCTTAGTTTGTTAATCCCTCTCCACTGAAACCGGGGAG     10555
BlkPLP1     AAAAATATTATGGATGCCTGGATCTTAGTTTGTTAATCCCTCTCCACTGAAACCGGAGAG     10572
WhtPLP1     AAAAATATTATGGATGCCTGGATCTTAGTTTGTTAATCCCTCTCCACTGAAACCGGAGAG     10565
GOHPLP1     AAAAATATTATGGATGCCTTGATCTTAGTTTGTTAATCCCTCTCCACTGAAACTGGAGAG     10561
            ******************* ********************************* ** ***

SumPLP1     ATTTCTTCCCTTGGAAGGGAACTTCTTGGAAGTGGTGAGAGTTTTCTTGGCCATTAACAC     10615
BlkPLP1     ATTTCTTCCCTTGGAAGGGAACTTCTTGGAAGTAGTGGGAGTTTTCTTGGCCGTTAACAC 10632
WhtPLP1     ATTTCTTCCCTTGGAAGGGAACTTCTTGGAAGTGGTGGGAGTTTTCTTGGCCATTAACAC     10625
GOHPLP1     ATTTCTTCCCTTGGAAGGGAACTTCTTGGAAGTGGTGGGAGTTTTCTTGGCCGTTAACAC     10621
            ********************************* *** ************* ********

SumPLP1     TGCTCTACTCTAGTTGACTGCTGTTCCCAGCCCAGAAGCAGCACATTTCAATAACAAACA     10675
BlkPLP1     TGCTCTACCCTAGTTGACTGCTGTTCCCAGCCCAGAAGCAGCACATTTCAATAACAAACA     10692
WhtPLP1     TGCTCTACCCTAGTTGACTGCTGTTCCCAGCCCAGAAGCAGCACATTTCAATAACAAACA     10685
GOHPLP1     TGCTCTACCCTAGTTGACTGCTGTACCCAGCCCAGAAGCAGCACATTTCAATAACAAACA     10681
            ******** *************** ***********************************
```

SumPLP1 CTAGATTTCACAACTGTTCAATACCACCTTCTATTTTTGATAAATCTTCAGAAAAGAAGA 10735
BlkPLP1 CTAGATTTCACAACTGTTCAATACCACCTTCTATTTTTGATAAATCTTCAGAAAAGAAGA 10752
WhtPLP1 CTAGATTTCACAACTGTTCAATACCACCTTCTATTTTTGATAAATCTTCAGAAAAGAAGA 10745
GOHPLP1 CTAGATTTCACAACTGTTCAATACCACCTTCTATTTTTGATAAATCTTCAGAAAAGAAGA 10741
************************************************************

SumPLP1 TCCTGATTGTTAGTAGGATCCAACTTTTCAGCCAGAATGATTAAAGATGGAAGAAAGGCA 10795
BlkPLP1 TCCTGATTGTTAGTAGGATCCAACTTTTCAGCCAGAACGATTAAAGATGGAAGAAAGGCA 10812
WhtPLP1 TCCTGATTGTTAGTAGGATCCAACTTTTCAGCCAGAACGATTAAAGATGGAAGAAAGGCA 10805
GOHPLP1 TCCTGATTGTTAGTAGGATCCAACTTTTCAGCCAGAATGATTAAAGATGGAAGAAAGGCA 10801
************************************* **********************

SumPLP1 CTGAAGGAAAGTCTCCGTGTAGCCCCACAGCTCTGTGAAGCTCACCCTGGTTGACTTGTG 10855
BlkPLP1 CTGAAGGAAATTCTCCGTGTAGCCCCACAGCTCTGTGAAGCTCACCCTTGTTGACTTGTG 10872
WhtPLP1 CTGAAGGAAATTCTCCGTGTAGCCCCACAGCTCTGTGAAGCTCACCCTTGTTGACTTGTG 10865
GOHPLP1 CTGAAGGAAAGTCTCCGTGTAGCCCCACAGCTCTGTGAAGCTCACTCTTGTTGACTTGTG 10861
********** ********************************** ** ***********

SumPLP1 TGTCTTACTTAGGTGTTCTCCCATGGAATGCTTTCCCTGGCAAGGTGTGTGGCTCCAACC 10915
BlkPLP1 TGTCTTACTTAGGTGTTCTCCCATGGAATGCTTTCCCTGGCAAGGTGTGTGGCTCCAACC 10932
WhtPLP1 TGTCTTACTTAGGTGTTCTCCCATGGAATGCTTTCCCTGGCAAGGTGTGTGGCTCCAACC 10925
GOHPLP1 TGTCTTACTTAGGTGTTCTCCCATGGAATGCTTTCCCTGGCAAGGTGTGTGGCTCCAACC 10921
************************************************************

SumPLP1 TTCTGTCCATCTGCAAAACAGCTGAGGTGAGTGGGCCATTGGGATTACTTTACAAAAGAA 10975
BlkPLP1 TTCTGTCCATCTGCAAAACAGCTGAGGTGAGTGGGCCATTTGGATTACTTTACAAAGGAA 10992
WhtPLP1 TTCTGTCCATCTGCAAAACAGCTGAGGTGAGTGGGCCATTTGGATTACTTTACAAAGGAA 10985
GOHPLP1 TTCTGTCCATCTGCAAAACAGCTGAGGTGAGTGGGCCATTTGGATTACTTTACAAAGGAA 10981
**************************************** *************** ***

SumPLP1 TGGCTAGTACTATACAAATTCCTACCCATGGCCTTCAATTTTAAGGACCACAGTTTCTCT 11035
BlkPLP1 TGGCTAGTACTATACAAATTCCTGCCCATGGCCTTCAATTTTAAGGACAACAGTTTCTCT 11052
WhtPLP1 TGGCTAGTACTATACAAATTCCTACCCATGGCCTTCAATTTTAAGGACAACAGTTTCTCT 11045

```
GOHPLP1     TGGCTAGTACTATACAAATTCCTACCCATGGCCTTCAATTTTAAGGACAACAGTTTCTCT          11041
            *********************** ************************ ***********

SumPLP1     TTGCTGGATTTTGAGTTAGCAGATTGCCTTCTGTAAAATATTGGCTGAGTATGCCTGAGC          11095
BlkPLP1     TTGCTGGATTTTGAGTTAGCAGATTGCCTTCCATAAAATACTGGCTGAGTATGCCTGAGC 11112
WhtPLP1     TTGCTGGATTTTGAGTTAGCAGATTGCCTTCCATAAAATACTGGCTGAGTATGCCTGAGC          11105
GOHPLP1     TTGCTGGATTTTGAGTTAGCAGATTGCCTTCCATAAAATATTGGCTGAGTATGCCTGAGC          11101
            *******************************  ******* *******************

SumPLP1     CAATGAACATAGATGGTAAAATATCTATTTTCCCTAGAGCTGCATAGAAAGCCTTCCATT          11155
BlkPLP1     CAATGAACATAGATGATAAAATATCTATTTTCCCTAGAGCTGCATAGAAAGCCTTCCATT          11172
WhtPLP1     CAATGAACATAGATGGTAAAATATCTATTTTCCCTAGAGCTGCATAGAAAGCCTTCCATT          11165
GOHPLP1     CAATGAACATAGATGGTAAAATATCTATTTTCCCTACAGCTGCATAGAAAGCCTTCCATT          11161
            *************** ******************** ***********************

SumPLP1     CCAAGACTTCCTCCCTTAAAAGAGAAATTTGTAGCTTTAGATAAAGATAGAGCTTAATGG          11215
BlkPLP1     CCAAGACTTCTTCCCTTAAAAGAGAAATTTGTAGCTTTAGGTAAAGATAGAGCTTAATGG          11232
WhtPLP1     CCAAGACTTCTTCCCTTAAAAGAGAAATTTGTAGATTTAGGTAAAGATAGAGCTTAATGG          11225
GOHPLP1     CCAAGACTTCCTCCCTTAAAAGAGAAATTTGCAGCTATAGGTAAAGATAGAGCTTAATGG          11221
            ********** ******************** ** * *** *******************
```

```
SumPLP1     AGAAATTCCCCAAATGAAAATTTTCATTCTTTTAGTTAAAAACGTTATCAAGAAAATGAG          11275
BlkPLP1     AGAAATTCCCCAAATGAAAATTTTCATTCTTTTAGTTAAAAACGTTATCAAGAAAATGAG          11292
WhtPLP1     AGAAATTCCCCAAATGAAAATTTTCATTCTTTTAGTTAAAAACGTTATCAAGAAAATGAG          11285
GOHPLP1     AGAAATTCCCCAAATGAAAATTTTCATTCTTTTAGTTAAAAACGTTATCAAGAAAATGAG          11281
            ************************************************************

SumPLP1     TTGTGCATAGAGACATTCATAGATTTACAAAACACTTTCACTTTGCAATTTCTAAATCT 11335
BlkPLP1     TTGTGCATAGAGACATTCATAGATTTACAAAATACTTTCGCTTTGGAATTTCTAAATCT 11352
WhtPLP1     TTGTGCATAGAGACATTCATAGATTTACAAAATACTTTCACTTTGGAATTTCTAAATCT 11345
GOHPLP1     TTGTGCATAGAGACATTCATAGATTTACGAAACACTTTCACTTTGGAATTTCTAAATCT          11341
            **************************** *** ****** ***** *************

SumPLP1     GTAATGAGAAAT--AAAAAAAAGTACATGTGAAAAAGGAAAATACTATATTGAGAATGGA          11393
BlkPLP1     CTAATGAGAAAT-AAAAAAAAAGTACATGTGAAAAAGGAAAATACTGTATTGAGAATGGA          11411
WhtPLP1     CTAATGAGAAATAAAAAAAAAAGTACATGTGAAAAAGGAAAATACTGTATTGAGAATGGA          11405
GOHPLP1     CTAATGAGAAATTAAAAAAAAAGTACACGTGAAAAAGGAAAATACTGTATTGAGAATGGA          11401
             ***********  ************* ****************** *************

SumPLP1     TATTATACACAAATGAGGCAAGGGAATAAAAATAAATCCTAAGGAAGAAGGCAGTAAATT          11453
BlkPLP1     TATTATACACAAATGAGGCAAGGGAATAAAAATAAATCCTAAAGAAGAAGGCAGTAAATT          11471
WhtPLP1     TATTATACACAAATGAGGCAAGGGAATAAAAATAAATCCTAAGGAAGAAGGCAGTAAATT          11465
GOHPLP1     TATTATACACAAATGAGGCAAGGGAATAAAAATAAATCCTAAGGAAGAAGGCAGTAAATT          11461
            ****************************************** *****************

SumPLP1     ATTTTTTTGAAAGAAAAATATTGACTTTATATTTGGGCATCCTGGGCACAACTGTAGGGA          11513
BlkPLP1     ATTTTTTTGAAAGCAAAATATTGACTTTATATTTGGGCATCCTGGGCACAACTGTAGGGA 11531
WhtPLP1     ATTTTTTTGAAAGCAAAATATTGACTTTATATTTGGGCATCCTGGGCACAACTGTAGGGA          11525
GOHPLP1     ATTTTTTTGAAAGCAAAATATTGACTTTATATTTGGGCATCCTGGGCACAACTGTAGGGA          11521
            ************* **********************************************

SumPLP1     ACCAATTGATTAAACCATTTTATGAGTATTTTATGAGTATTCCTTGCTCTGGGTGCTATA 11573
BlkPLP1     ACCAATTGATTAAAACATTTTATGAGTATTTTATGAGTATTCCTTGCTCTGGGTCCTATA 11591
WhtPLP1     ACCAATTGATTAAAACATTTTATGAGTATTTTATGAGTATTCCTTGCTCTGGGTGCTATA 11585
```

GOHPLP1 ACCAATTGTTTAAAACATTTTATGAGTATTTTATGAGTATTCCTTGCTCTGGGTGCTATA 11581
******** ***** *************************************** *****

SumPLP1 GGGCTAGATGCTATGGTGTACATTGAAGAATGGAGGCCTAGACTCAGGGAAGCAACAAC 11633
BlkPLP1 GGGCTAGATGCTATGGTGTACATTGAAGAGTTGGAGGCCTACAATCAGGGAAG---CAAC 11648
WhtPLP1 GGGATAGATGCTATGGTGTACATTGAAGAGTTGGAGGCCTACAATCAGGGAAGCAACAAC 11645
GOHPLP1 GGGCTAGATGCTATGGTGTACACTGAAGAGTGGGAGGCCTACAATCAGGGAAGCAACAAC 11641
*** ****************** ****** * ********* * ********* ****

SumPLP1 TTAGAGATGCAGAATTAACACACAGAAAGATATCAACACATTCAGAAAGCTGCATTCACG 11693
BlkPLP1 TTAGAGATGCAGAATTAACACACAGAAATATATCAACACATTCAGAAAGCTGCATTCACG 11708
WhtPLP1 TTAGAGATGAAGAATTAACACACAGAAAGATATCAACGCATTCAGAAAGCTGCATTCACG 11705
GOHPLP1 TTAGAGATGCAGAATTAACACACAGAAAGATATCAACACATTCAGAAAGCTGCATTCACA 11701
********* ****************** ******** *********************

SumPLP1 ATCTACAGTAGAATATATTTTTGCTGTTGCAAGAAACAGTTCTCCCTTTTGCATTTTCTT 11753
BlkPLP1 ATCTACAGTAGAACATATTTTTGCTGTTGCAAGAAACAGTTCTCCCTTTTGCATTTTCTT 11768
WhtPLP1 ATCTACAGTAGAACATATTTTTGCTGTTGCAAGAAACAGTTCTCCCTTTTGCATTTTCTT 11765
GOHPLP1 ATCTACAGTAGAACATATTTTTGCTGTTGCAAGAAACAGTTCTCCCTTTTGCATTTTCTT11761
************* **********************************************

```
SumPLP1   GCAGTTCCAAATGACCTTCCACCTGTTTATTGCTGCGTTTGTGGGGGCTGCAGCCACACT   11813
BlkPLP1   GCAGTTCCAAATGACCTTCCACCTGTTTATTGCTGCGTTTGTGGGGGCTGCAGCCACACT 11828
WhtPLP1   GCAGTTCCAAATGACCTTCCACCTGTTTATTGCTGCGTTTGTGGGGGCTGCAGCCACACT   11825
GOHPLP1   GCAGTTCCAAATGACCTTCCACCTGTTTATTGCTGCGTTTGTGGGGGCTGCAGCCACACT   11821
          ************************************************************

SumPLP1   GGTTTCCCTGGTGAGTTGTCTTTGAATGATCTTGGCAAATAAATGGTCCTGGGATAGCTT   11873
BlkPLP1   GGTTTCCCTGGTGAGTTGTCTTTGAATGATCTTGGCAAATAAATGGTCCTGGGATAGCTT 11888
WhtPLP1   GGTTTCCCTGGTGAGTTGTCTTTGAATGATCTTGGCAAATAAATGGTCCTGGGATAGCTT   11885
GOHPLP1   GGTTTCCCTGGTGAGTTGTCTTTGAATGATCTTGGCAAATAAATGGTCTTGGGATAGCTT   11881
          ************************************************ ***********

SumPLP1   GGGTACAGCTATGCTGAAAAGCAAAAATATAGACTCTTTCTAACCTTGAAATGCTAGAGA   11933
BlkPLP1   GGGTACAGCTATGCTGAAAGGCAAAAATATAGACTCTTTCTAACATTGAAATGCTAGAGA   11948
WhtPLP1   GGGTACAGCTATGCTGAAAGGCAAAAATATAGACTCTTTCTAACCTTGAAATGCTAGAGA   11945
GOHPLP1   GGGTACAGCTATGCTGAAAGGCAAAAATATAGACTCTTTCTAACCTTGAAATGCTAGAGA   11941
          ******************* ************************ ***************

SumPLP1   GAAGCTACTGGGAAGAGACAGCAAAGTGTAGAGACTGTGCCCTTCTACTTTGTGTTGGCA   11993
BlkPLP1   GAAGCTACTGGGAAGAGACAGTAAAGTGTAGAGACTGTGCCCTTCTACTTTGGGTTGGCA   12008
WhtPLP1   GAAGCTACTGGGAAGAGACAGTAAAGTGTAGAGACTGTGCCCTTCTACTTTGGGTTGGCA   12005
GOHPLP1   GAAGCTACTGGGAAGAGACAGCAAAGTGTAGAGACTGTGCCCTTCTACTTTGGGTTGGCA   12001
          ********************* ****************************** *******

SumPLP1   AGGTAGAGAAACTTCAGAAATTAGATAGGAACAAGGAAAGTGGTATGAAGAAAGAGGAGG   12053
BlkPLP1   AGGTAGAAAAACTTCAGAAATTAGATAGGAACAAGAAAAGTGGTATGAAGAAAGAGGAGG   12068
WhtPLP1   AGGTAGAAAAACTTCAGAAATTAGATAGGAACAAGAAAAGTGGTATGAAGAAAGAGGAGG   12065
GOHPLP1   AGGTAGAAAAACTTCAGAAATTAGATAGGAACAAGAAAAGTGGTATGAGGAAAGAGGAGG   12061
          ******* ************************** ************* ***********

SumPLP1   TAAGATATTAATAGTCAATAATAGTAATATTTGGCATTTACAGAGCATGTAATATTGCCA   12113
BlkPLP1   TAAGATATTAATAGTCAATAATAATAATATTTGGCATTTACAGAGCATGTAATATTGCCA   12128
WhtPLP1   TAAGATATTAATAGTCAATAATAATAATATTTGGCATTTACAGAGCATGTAATATTGCCA   12125
```

```
GOHPLP1     TAAGATATTAATAGTCAATAATAATAATATTTGGCATTTACAGAGCATGTAATATTGCCA     12121
            ********************* **************************************

SumPLP1     AGGACTTTGCAGTCATAAATTATGGTTAATTCTCAACACATCTTTGTAAGATGGGTGATA     12173
BlkPLP1     AGGACTTTGCAGTCATAAATTATGGTTAATTCTCAACACATCTTTGTCAGATGGGTGATA 12188
WhtPLP1     AGGACTTTGCAGTCATAAATTATGGTTAATTCTCAACACATCTTTGTCAGATGGGTGATA     12185
GOHPLP1     AGGACTTTGCAGTCATAAATTATGGTTAATGCTCAACACATCTTTGTCAGATGGGTGATA     12181
            ****************************** **************** ************

SumPLP1     AGAACACTGAAACATTACTAAACATGATTTTCCAAAGCCTGGAATTCTATATACTGTAAA     12233
BlkPLP1     AGAACACTGAAACATTACTAAACATGATTTTCCAAAGCCTGGAATTCTATATACTGTAAA     12248
WhtPLP1     AGAACACTGAAACATTACTAAACATGATTTTCCAAAGCCTGGAATTCTATATACTGTAAA     12245
GOHPLP1     AGAACACTGAAACATTACTAAACATGATTTTCCAAAGCCTGGAATTCTATATACTGTAAA     12241
            ************************************************************

SumPLP1     TATGAATCCATCTTGGACACTCTCCAAGAGATTTAGTTACTCTTCCAAAAATCCTGGAAG     12293
BlkPLP1     TATGAATCCATCTTGGACACTCTCCAAGAGATTTAGTTACTCTTCCAAAAATCCTGGAAG12308
WhtPLP1     TATGAATCCATCTTGGACACTCTCCAAGAGATTTAGTTACTCTTCCAAAAATCCTGGAAG     12305
GOHPLP1     TATGAATCCATCTTGGACACTCTCCAAGAGATTTAATTACTCTTCCAAAAATCCTGGAAG     12301
            *********************************** ************************
```

```
SumPLP1   GACCTCCTAACATTTAGAATTGTTACTACTATAATGCAATTCTATGTCCCAGGTCCTGCT 12353
BlkPLP1   GACCTCCTAACATTTAGAATTGTTACTACTATAATGCAATTCTATGTCCCAGGTCCTGCT 12368
WhtPLP1   GACCTCCTAACATTTAGAATTGTTACTACTATAATGCAATTCTATGTCCCAGGTCCTGCT 12365
GOHPLP1   GACCTCCTAACATTTAGAATTGTTACTACTATAATGCAATTCTATGTCCCAGGTCCTGCT 12361
          ************************************************************

SumPLP1   GATGGTGTTACAGTGCAGCGTCAATGTAGTGCATTCATTGTTCAGATTAAAAACCTAGGG 12413
BlkPLP1   GATGGTGTTACAGTGCAGTGTCAATGTAGTGCATTCATTGTTCAGATTAAAAACCTAGGG 12428
WhtPLP1   GATGGTGTTACAGTGCAGTGTCAATGTAGTGCATTCATTGTTCAGATTAAAAACCTAGGG 12425
GOHPLP1   GATGGTGTTACAGTGCAGTGTCAATGTAGTGCATTCATTGTTCAGATTAAAAACCTAGGG 12421
          ****************** *****************************************

SumPLP1   CACTGAAGGATGAAACTATTTATCTGTAGTCATTTGAATAAATGGTGAATGTTCAAGGAT 12473
BlkPLP1   CACTGAAGGATAAAACTATTTATCTGTAGTCATTTGAATAAATGGTGAATGTTCAAGGAT 12488
WhtPLP1   CACTGAAGGATAAAACTATTTATCTGTAGTCATTTGAATAAATGGTGAATGTTTAAGGAT 12485
GOHPLP1   CACTGAAGGATGAAACTATTTATCTGTAGTCATTTGAATAAATGGTGAATGTTCAAGGAT 12481
          *********** ****************************************** ******

SumPLP1   AAACTCGCAAAAATCCTAGCCCCTACTGCTGAAGCTTTTATACGGAGTCGAGTTGTTTCT 12533
BlkPLP1   AAGCTCGCAAAAATCCTAGCCCGTACTGCTGAAGCTTTTATACGGAGTTGAGTTGTTTCT 12548
WhtPLP1   AAGCTCGCAAAAATCCTAGCCCCTACTGCTGAAGCTTTTATACGGAGTTGAGTTGTTTCT 12545
GOHPLP1   AAACTCGCAAAAATCCTAGCCCCTACTGCTGAAGCTTTTATACGGAGTCGAGTTGTTTCC 12541
          ** ****************** ************************** ********** 

SumPLP1   CATTTTGTGAAATAAATTGATGCATTAAGCAGGGGAGGGCTGTGATGCACAGGGTGGCTG 12593
BlkPLP1   CATTTTGTAAAATAAATTGATGCATTAAGCAGGGGAGGGCTGTGATGCACACAGTGGCTG 12608
WhtPLP1   CATTTTGTAAAATAAATTGATGCATTAATCAGGGGAGGGCTGTGATGCACACAGTGGCTG 12605
GOHPLP1   CATTTTTAAAAATAAATTGATGCATTAAACAGGGGAGGGCTGTGATGCACAGGGTGGCTG 12601
          ******   ******************* **********************  *******

SumPLP1   TGTGTCTGCAGCAAGTACCCTATGACTTCAGGTCATTCAAACCTGTGGCTATGTTGATGG 12653
BlkPLP1   TGTGTCTGCAGCAAGTACCCTATGACTTCAGGTCATTCAAACCTGTGGCTATGTTGATGG 12668
WhtPLP1   TGTGTCTGCAGCAAGTACCCTATGACTTCAGGTCATTCAAACCTGTGGCTATGTTGATGG 12665
```

```
GOHPLP1   TGTGTCTGCAGCAAGTACCCTATGACTTCAGGTCATTCAAACCTGTGGCTATGTTGATGG   12661
          ************************************************************

SumPLP1   CAGAGCCCACGTCTTGTTCACTTCCCTGAGGAAAATTCAGTACTAGGCTGAGCATAAGTG   12713
BlkPLP1   CAGAGCCCACGTCTTGTTCACTTCCCTGAGGAAAATTCAGTACTAGGCTGAGCATAAGTG   12728
WhtPLP1   CAGAGCCCACGTCTTGTTCACTTCCCTGAGGAAAATTCAGTACTAGGCTGAGCATAAGTG   12725
GOHPLP1   CAGAGCCCACATCTTGTTCACTTCCCTGAGGAAAATTCAGTACTAGGCTGAGCATAAGTG   12721
          ********** *************************************************

SumPLP1   TGGGATAAATCTTTGCCGAGTTCATTCTCTGGGCCTGGGCCATCGGAAGGAAACGTCTCA   12773
BlkPLP1   TGGAATAAATCTTTGCCGAGTTCATTCTCTGGGCCTGGGCCATCGGAAGGAAACGTCTCA   12788
WhtPLP1   TGGAATAAATCTTTGCCGAGTTCATTCTCTGGGCCTGGGCCATCGGAAGGAAACGTCTCA   12785
GOHPLP1   TGGAATAAATCTTTGCTGAGTTCATTCTCTGGGCCTGGACCATTGGAAGGAAATGTCTCA   12781
          *** ************ ********************* **** ********* ******

SumPLP1   GAGAGGACTTCTTGTCAAGTATGTGAAGTAAAAGCCTGGGACGTGCTGCTTTCTGCAGAG   12833
BlkPLP1   GAGAGGACTTCTTGTCAAGTGTGTGAAGTAAAAGCCTGGGACGTGCTGCTTTCTGCAGAG   12848
WhtPLP1   GAGAGGACTTCTTGTCAAGTGTGTGAAGTAAAAGCCTGGGACGTGCTGCTTTCTGCAGAG   12845
GOHPLP1   GAGAGGACTTCTTGTCAA-TATGTGAAGTAAAAGCCTGGGACGTACTGCTTTCTGCAGAG   12840
          ****************** * *********************** ***************
```

```
SumPLP1   TAGGGGTTTTCCGTTTATTTAGCTATGACCTTATTTCTACCCTTCCTCATTCCCAAAAGG 12893
BlkPLP1   TAGGGGTTTTCCGTTTATTTAGCTATGACCTTATTTCTACCCTTCCTCATTCCCAAAAGG 12908
WhtPLP1   TAGGGGTTTTCCGTTTATGTAGCTATGACCTTATTTCTACCCTTCCTCATTCCCAAAAGG 12905
GOHPLP1   TAGGGGTTTTCCGTTTATTTAGCTATGACCTTATTTCTACCCTTCCTCATTCCCAAAAGG 12900
          ***************** ******************************************

SumPLP1   ATTGGAGGAGGGAGAGATTCCTTTTCTACTGTCACTCATATTCTCTCTTCTGTTCCCTAC 12953
BlkPLP1   ATTGGAGGAGGGAGAGCTTCCTTTTCTACTGTCACTCATATTCTCTCTTCTGTTCCCTAC 12968
WhtPLP1   ATTGGAGGAGGGAGAGCTTCCTTTTCTACTGTCACTCATATTCTCTCTTCTGTTCCCTAC 12965
GOHPLP1   ATTGGAGGAGGGAGAGCTTCCTTTTCTACTGTCACTCATATTCTCTCTTCTGTTCCCTAC 12960
          **************** *******************************************

SumPLP1   AGCTCACCTTCATGATTGCTGCCACTTACAACTTTGCCGTCCTTAAACTCATGGGCCGAG 13013
BlkPLP1   AGCTCACCTTCATGATTGCTGCCACTTACAACTTTGCCGTCCTTAAACTCATGGGCCGAG 13028
WhtPLP1   AGCTCACCTTCATGATTGCTGCCACTTACAACTTTGCCGTCCTTAAACTCATGGGCCGAG 13025
GOHPLP1   AGCTCACCTTCATGATTGCTGCCACTTACAACTTTGCCGTCCTTAAACTCATGGGCCGAG 13020
          ************************************************************

SumPLP1   GCACCAAGTTCTGATCTCCTGTAGAAATTTCCCTTTCTCTAATAGCGAGGCTCTAACCTA 13073
BlkPLP1   GCACCAAGTTCTGATCTCCTGTAGAATTTTCCTTTTCTCTAATAGCGAGGCTCTAACCTA 13088
WhtPLP1   GCACCAAGTTCTGATCTCCTGTAGAATTTTCCTTTTCTCTAATAGCGAGGCTCTAACTTA 13085
GOHPLP1   GCACCAAGTTCTGATCTCCTGTAGAAATTTCCCTTTCTCTAATAGTGAGGCTCTAACCTA 13080
          ************************** ***** ************ *********** **

SumPLP1   CACAGCCTACAACTCTGCATCTCCCGTGTTAACTCTTTGCCTTTGCCACTGACTGGCCTT 13133
BlkPLP1   CACAGCCTACAACTCTGCGTCTCCTGTGTTAACTCTTTGCCTTTGCCACTGACTGGC--- 13145
WhtPLP1   CACAGCCTACAACTCTGCGTCTCCTGTGTTAACTCTTTGCCTTTGCCACTGACTGGCCTT 13145
GOHPLP1   CACAGCCTACAACTCTGCGTCTCCCGTGTTAACTCTTTGCCTTTGCCACTGACTGGCCTT 13140
          ****************** ***** ********************************

SumPLP1   CTTCTTACTTGATGAGTGTAACAAGAAAGGAGAGTCTTGTGGTGATTAACGTCTCTCTGT 13193
BlkPLP1   CTTCTTACTTGATGAGTGTAACAAGAAAGGAGAGTCTTGCGGTGATTAACGTCTCTCTGT 13205
WhtPLP1   CTTCTTACTTGATGAGTGTAACAAGAAAGGAGAGTCTTGCGGTGATTAACGTCTCTCTGT 13205
```

```
GOHPLP1     CTTCTTACTTGATGAGTGTAACAAGAAAGGAGAGTCTTGCAGTGATTAACGTCTCTCTGT          13200
            ************************************* ******************

SumPLP1     GGACTCTCCCCTCTTACATACCTCTTTTAGTCATTTTGCTTCACAGCTCGTTCCTGCTAG 13253
BlkPLP1     GGACTCTCCCCTCTTACATACCTCTTTTAGTCATTTTGCTTCACAGCTGGTTCCTGCTAG 13265
WhtPLP1     GGACTCTCCCCTCTTACATACCTCTTTTAGTCATTTTGCTTCACAGCTGGTTCCTGCTAG 13265
GOHPLP1     GGACTCTCCCCTCTTACATACCTCTTTTAGTCATTTTGCTTCACAGCTGGTTCCTGCTAG 13260
            ********************************************** ***********

SumPLP1     AAATGGGAAATGCTTAAGATGGTGACTCCTCAACTGCAAGTCACAAAGAAATGGAGGCTC          13313
BlkPLP1     AAATGGGAAATGCTTAAGATGGTGACTCCTCAACTGCAAGTCACAAAGAAATGGAGGCTC          13325
WhtPLP1     AAATGGGAAATGCTTAAGATGGTGACTCCTCAACTGCAAGTCACAAAGAAATGGAGGCTC          13325
GOHPLP1     AAATGGGAAATGCTTAAGATGGTGACTCCTCAACTGCAAGTCATAAAGAAATGGAGGCTC          13320
            ****************************************** ****************

SumPLP1     TAATTCAATTTTGAAGCATCTCCTGAGGACCAGAAAGTGTTTCTTCTCAAAGGGCTCTT           13373
BlkPLP1     TAATTCAATTTTCAAGCATCTCCTGAGGACCAGAAAGTGTTTCTTCTCAAAGGGCTCTT 13385
WhtPLP1     TAATTCAATTTTCAAGCATCTCCTGAGGACCAGAAAGTGTTTCTTCTCAAAGGGCTCTT 13385
GOHPLP1     TAATTCAATTTTGAAGCATCTCCTGAGGACCAGAAAGTGTTTCTTCTCAAAGGGCTCTT           13380
            *********** **********************************************
```

```
SumPLP1   CCACTGATGGAAACAAAGTGGAGAGAAAAATGCTCAGGTAGAGAGAAGGGATGTACCCGG   13433
BlkPLP1   CCACTGATGGAAACAAAGTGGAGAGAAAAATGCTCAGGTAGAGAGAAGGGATGTACCCGG   13445
WhtPLP1   CCACTGGTGGAAACAAAGTGGAGAGAAAAATGCTCAGGTAGAGAGAAGGGATGTACCCGG   13445
GOHPLP1   CCACTGATGGAAACAAAGTGGAGAGAAAAATGCTCAGGTAGAGAGAAGGGATGTACCCGG   13440
          ****** *****************************************************

SumPLP1   TCCCCTTGCCATCTATAGGGTAGGGGCCAAATATATTATCCTTAGTGTACAAAGCAGAGA   13493
BlkPLP1   CCCCCTTGCCATCTATAGGGTAGGGGCCAAATATATTCTTCTTAGTGTACAAAGCAGAGA   13505
WhtPLP1   CCCCCTTGCCATCTATAGGGTAGGGGCCAAATATATTCTTCTTAGTGTACAAAGCAGAGA   13505
GOHPLP1   TCCCCTTGCCATCTATAGGGTAGGGGCCAAATATATTCTCCTTAGTGTACAAAGCAGAGA   13500
           ************************************ * ********************

SumPLP1   ACTCACTCCTGTCTCCCTATTACCACTGAAGATAGAAGAAAAAAA   13538
BlkPLP1   ACTCACTCCTGTCTCCCTATTACCACTGAAGATAGAAGAAAAAAA   13550
WhtPLP1   ACTCACTCCTGTCTCCCTATTACCACTGAAGATAGAAGAAAAAAA   13550
GOHPLP1   ACTCACTCCTGTCTCCCTATTACCACTGAAGATAGAAGAAAAAAA   13545
          *********************************************
```

**Supplementary Fig. S2.** Multiple sequence alignment of the sex determining region for Y (SRY) for Sumatran (Sum), black (Blk), and greater one-horned (GOH) rhinos. An asterisk indicates a position that is fully conserved across the 3 species. Oligonucleotide sequences used for qPCR assay indicated in red with bolded text to denote primer locations and underlined text for the probe location.

SumSRY AGAAATA**ACCTTGGACTAGTAAGATAAGTTTCC**TAGTGCTTACACCTTCTCATTTTGCTA 60
GOHSRY AGAAATA**ACCTTGGACTAGTAAGATAAGTTTCC**CACTGCTTACAGCTTCTCATTTTGCTA 60
BlkSRY ------------------------------------------------------------ 0

SumSRY CTGTCTCCCCCTCTTCGACGGTGCCATCTTATGCCTCTGCTATGTTCA**GGGTATCTAGCA**120
GOHSRY CTGTCTCCCCCTCTTCGACGGTGCCATCTTATGCTTCTGCTATGTTCA**GGGTATCTAGCA** 120
BlkSRY ------------------------------------------------------------ 0

SumSRY **GCGATGATTAC**AGCCCAGAGGGACGGGAACAAACTGTTCTCGCCTCGGGGAGAACCTCTC 180
GOHSRY **GCGATGATTAC**AGCCCAGAGGGACAGCAACAAACTGTTCTCGCCTCCGGGAGAATCTCTC 180
BlkSRY ------------------------------------------------------------ 0

SumSRY CCCTACTGTGGACCGGCAACGCTGGCTCAGATGATCGGAGAGAAACAGCGGGAAATGGTA 240
GOHSRY CCCTACTCTGGCCCGGTAACGCTGGCTCAGATGATCGGAGAGAAACAGCGGGAAATGGTA 240
BlkSRY ---------------------CTGGCTCAGATGATCGGAGAGAAACAGCGGGAAATGGTA 39
***************************************

SumSRY GAGAGAGCGGCCAGGACCGCGTCAAACGGCCCATGAATGCATTCATGGTGTGGTGTCGTG 300
GOHSRY CAGAGAGCGGCCAGGACCGTGTCAAACGGCCCATGAATGCATTCATGGTGTGGTCTCGTG 300
BlkSRY GAGAGAGCGGCCAGGACCGTGTCAAACGGCCCATGAACGCATTCATGGTGTGGTCTCGTG 99
***************** **************** *************** *****

SumSRY ATCACAGGCGCAAAATGGCTCTGGAGAATCCCCAAATGCAAAACTCGGAGATCAGCAAGC 360
GOHSRY ATCACAGGCGCAAAGTGGCTCTAGAGAATCCCCAAATGCAAAACTCGGAGATCAGCAAGC 360
BlkSRY ATCACAGGCGCAAAGTGGCTCTAGAGAATCCCCAAATGCAAAACTCGGAGATCAGCAAGC 159

************** ******* *************************************

SumSRY GGCTGGGACGCCAGTGGAAAATGCTTACGGAAGCCGAAAAATGGCCTTTCTTCGAGGAGG 420
GOHSRY GGCTGGGATGCCAGTGGAAAATGCTTACGGAAGCCGAAAAATGGCCTTTCTTCGAGGAGG 420
BlkSRY GGCTGGGATGCCAGTGGAAAATGCTTACGGAAGCCGAAAAATGGCCTTTCTTCGAGGAGG 219
******** ***************************************************

SumSRY CACAGAGACTACGGGCCGTGCACCAAGAGAAATACCCGGACTATAAATATCGACCTCGGA 480
GOHSRY CACAGAGACTACGGGCTGTGCACCAAGAGAAATACCCGGACTATAAATATCGACCTCGGA 480
BlkSRY CACAGAGACTACGGGCCGTGCACCAAGAGAAATACCTGGACTATAAATATCGACCTCGGA 279
*************** ******************** ***********************

SumSRY GGAAGGCCAAGATACCACAGAAAAGTGACAAATCGCTTCCCGCAGACTCCTCTGCGATAG 540
GOHSRY GGAAGGTCAAGATACCACAGAAAAGTGACAAATCGCTTCCCGCAGACTCCTCAGCGATAG 540
BlkSRY GGAAGGCCAAGATACCACAGAAAAGTGACAAATCGCTTCCCGCAGACTCCTCTGCGATAG 339
****** ********************************************* *******

SumSRY TCTGCAGCCAGGCGCACGTGGACGAGAGGTTGTACCCCTTCACATACAGAGATGGCTGTA 600
GOHSRY TCTGCAGCCAGGCGCACGTGGACGAGAGGTTGTACCCCTTCACATACAGGGATGGCTGTA 600
BlkSRY TCTGCAGCCAGGCGCACGTGGACGAGAGGTTGTACCCCTTCACATACAGGGATGGCTGTG 399
************************************************* *********

SumSRY CTAAGGCCACACAGTCGCGAACGGACCGCCCGTTATGCCGCTCACAGCCCATGAACACAG 660
GOHSRY CTCAGGCCACACAGTCGCGAACAGACCGCCCGTTATGCCGCTCACAGCCCGTGAACACAG 660
BlkSRY CTAAGGCCACACAGTTGCGAACAGACCGCCCGTTATGCCGCTCACAGCCCGTGAACACAG 459
** ************ ****** *************************** *********

SumSRY CCAGCTCGCCGCTGGGACAGCACCGTCACAGCAGCTCCGCAAGCCTGCGTGACCTTCGGG 720
GOHSRY CCAGCTCGTCGTTGGGACAGCACCGTCACAGCAGCTCCGCAAGCCTGCATGACCTTCGGG 720
BlkSRY CCAGCTCGCCGTTGGGACAGCACCGTCACAGCAGCTCCGCAAGCCTGCGTGACCTTCCGG 519
******** ** ************************************ ******** **

SumSRY TAACGCTGGCTACGCAGACCTACATAGACGTTCCCTTTCACCGTTAACCTACAGCCCGGA 780

```
GOHSRY     TAACGCTGGCTACGCAGACCTACGTAGACGTTCCCTGTCACCGTAA-CGTACAGCCCGGA            779
BlkSRY     TAACGCTGGCTACGCAGAGCTACGTAGACGTTCCCTTTCACCGTAA-CTTACAGCCCGGA  578
           ***************** **** ************ ******* * * ***********

SumSRY     CTTGCTCACGTTTATTTTCC  800
GOHSRY     CTTGCTCACGTGTATTTTCC 799
BlkSRY     CTTGCTCACGTTTATTTTCC    598
           *********** ********
```

**Supplementary Fig. S3.** Diagram of the 2-round three primer multiplexing method. In the first round, a microsatellite is amplified using a forward primer incorporating a unique tailed sequence on the 5' end (Forward Tailed Primer) and a reverse primer. During the next 29 PCR cycles, multiple copies of the tailed fragment is generated. For the second round of PCR, fluorescently tagged primers for the unique tail sequence (Labeled Universal Primer) is added to the reaction. The universal primers combine with the reverse primary to amplify and label the fragments from round 1 with the fluorescent tag. A final extension cycle is performed to ensure that the majority of amplicons are properly amplified and labeled.

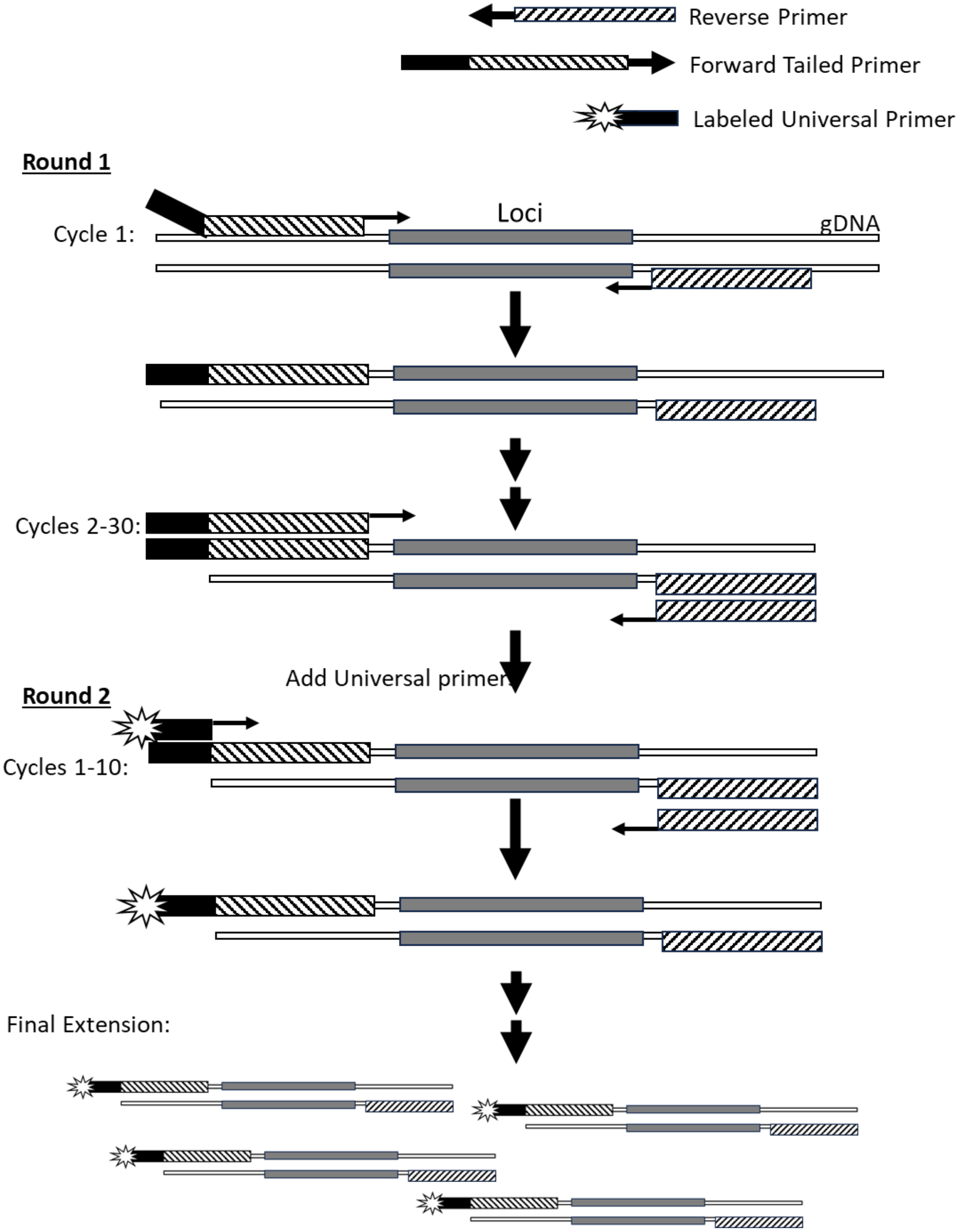

**Supplementary Table S1**

| Genetic Marker for: | Gene | Forward Primer ID | Forward Primer Sequence (5’-3’) |
|---|---|---|---|
| Female | Proteolipid protein 1 | Rh_PLP1_FWD | GCTGCCACTTACAACTTTGC |
| Male | Sex-determining region Y | Rh_SRY_FWD | ACCTTGGACTAGTAAGATAAGTTTCC |

| Reverse Primer ID | Reverse Primer Sequence (5'-3') | Probe ID |
|---|---|---|
| Rh_PLP1_REV | AGGTTAGAGCCTCGCTATTAGA | Rh_PLP1_probe |
| Rh_SRY_REV | GTAATCATCGCTGCTAGATACCC | Rh_SRY_probe |

| **Probe Sequence (modification locations indicated)*** | **Amplicon length** |
| --- | --- |
| 5'**Sun-**CGTCCTTAA(**ZEN™**)ACTCATGGGCCGAGG-**IowaBlack®FQ**3' | 102 bp |
| 5'**FAM**-ACGGTGCCA(**ZEN™**)TCTTATGCTTCTGCT-**IowaBlack®FQ**3' | 124 bp |

*fluorescent tags in bold

**Supplementary Table S2**

| Genetic Marker for: | Gene | Forward Primer ID |
|---|---|---|
| *Diceros bicornis* (Black Rhino) | Cytochrome b | Rh_BR_FWD |
| *Ceratotherium simum* (White Rhino) | Cytochrome b | Rh_WR_FWD |
| *Rhinoceros unicornis* (GOH Rhino) | Cytochrome b | Rh_GOH_FWD |

| Forward Primer Sequence (5’-3’) | Reverse Primer ID | Reverse Primer Sequence (5'-3') | Amplicon length |
|---|---|---|---|
| AATCTGCCTAATCCTACAAATC | Rh_BR_REV | GGTTTCTAGGAAGGTGTAGG | 222 |
| CCACTCATTCATCGATCTGC | Rh_WR_REV | TAATAGATACCGCGTCCTAC | 266 |
| TCTCACCCACTAGTTAAAATCA | Rh_GOH_REV | AGGAAGGTGTAAGATCCATAG | 310 |

**Reference**

Ewart KM et al. (2018) PLoS ONE 13(6):e0198565

**Supplementary Table S3**

| # | Locus | Accession | Forward Primer ID |
|---|---|---|---|
| 1 | 32A | AY138541 | 32A_FWD |
| 2 | 32F | AY138542 | 32F_FWD |
| 3 | 12F | AY138545 | 12F_FWD |
| 4 | BlRh37D | AY606083 | BlRh37D_FWD |
| 5 | DB52 | AF129732 | DB52_FWD |
| 6 | SR281 | AY427974 | SR281_FWD |
| 7 | IR22 | | IR22_FWD |
| 8 | BlRh1C | AY606079 | BlRh1C_FWD |
| 9 | SR74 | AY427967 | SR74_FWD |
| 10 | RH12 | AJ508905 | RH12_FWD |
| 11 | 7C | AY138543 | 7C_FWD |
| 12 | SR262 | AY606077 | SR262_FWD |
| 13 | DB1 | AF129724 | DB1_FWD |
| 14 | DB44 | AF129730 | DB44_FWD |
| 15 | BlRh1B | AY606078 | BlRh1B_FWD |
| 16 | BR6 | | BR6_FWD |
| 17 | DB66 | AF129733 | DB66_FWD |
| 18 | SR63 | AY427965 | SR63_FWD |
| 19 | DB23 | AF129734 | DB23_FWD |
| 20 | SR268 | AY427972 | SR268_FWD |
| 21 | IR10 | | IR10_FWD |
| 22 | IR12 | | IR12_FWD |
| 23 | 7B | AY138544 | 7B_FWD |

| Locus Specific Forward Primer with 5' Universal Tail* (5'-3') | Reverse Primer ID |
|---|---|
| **GCCTCCCTCGCGCCA**CTAGCAAAATCTCAAAGAGG | 32A_REV |
| **GCCTTGCCAGCCCGC**GGCAAAACTAAGAGAACTTG | 32F_REV |
| **GCCTTGCCAGCCCGC**ACAGCTAGAATCACCAAAAC | 12F_REV |
| **CAGGACCAGGCTACCGTG**ACATGTGTAAACTTGGGAAC | BlRh37D_REV |
| **CGGAGAGCCGAGAGGTG**CATGTGAAATGGACCGTCAGG | DB52_REV |
| **GCCTCCCTCGCGCCA**AGGTGATTAGGGAATTGCTGG | SR281_REV |
| **GCCTTGCCAGCCCGC**ATGGTGGAAGAAGTGCAGCC | IR22_REV |
| **CAGGACCAGGCTACCGTG**AGATTCTTGGAAAGGTCACT | BlRh1C_REV |
| **CAGGACCAGGCTACCGTG**CAGCACAATGTTTGGCACTTG | SR74_REV |
| **CGGAGAGCCGAGAGGTG**CTGGTGCATTCATCAGGGCT | RH12_REV |
| **GCCTCCCTCGCGCCA**GTCAGTTCAAGTTTTTGCTC | 7C_REV |
| **GCCTCCCTCGCGCCA**CTGCCTTAACAACTGAACTGC | SR262_REV |
| **GCCTTGCCAGCCCGC**TAAGTCACAGGGACTAATCTG | DB1_REV |
| **GCCTTGCCAGCCCGC**AGGGTGGAATGTCAAGTAG | DB44_REV |
| **CAGGACCAGGCTACCGTG**GATCAGTAACACCAAAGTCC | BlRh1B_REV |
| **CGGAGAGCCGAGAGGTG**TCATTTCTTTGTTCCCCATAGCAC | BR6_REV |
| **CGGAGAGCCGAGAGGTG**CCAGGTGAAGGGTCTTATTATTAGC | DB66_REV |
| **GCCTCCCTCGCGCCA**CTTGAGCAGAGTAGAATTTGG | SR63_REV |
| **GCCTCCCTCGCGCCA**ATCTTCCTCAGCAATAAGG | DB23_REV |
| **GCCTTGCCAGCCCGC**GTTTATACTATGCCCTGCAC | SR268_REV |
| **CAGGACCAGGCTACCGTG**CAGTGAGGAAGATTGGTTGC | IR10_REV |
| **CGGAGAGCCGAGAGGTG**GAATGCTGATCATTTAGTGAC | IR12_REV |
| **CGGAGAGCCGAGAGGTG**AACCAACTTGTAATGAGAGG | 7B_REV |

**5' universal primer sequence bolded and underlined*


| References | Reference for: |
|---|---|
| Harper et al. (2013) FSI Genetics 7(4): P428-433 | loci primers 1-9, |
| Harper et al. (2018) Current Biology 28:R1-R16 | locus primer 10 |
| Blacket et al. (2012) Molecular Ecology Resources 12:456-463 | universal primer |

| Locus Specific Reverse Primer (5'-3') | Multiplex Panel # | Universal Primer ID | Universal Primer Sequence** |
|---|---|---|---|
| TTACTAAGGGAATCACCAAG | 1 | Tail A | 5'**FAM**-GCCTCCCTCGCGCCA |
| GATACCAAACTGGAAATGG | 1 | Tail B | 5'**VIC**-GCCTTGCCAGCCCGC |
| TCCTGCTGCATAAATCTC | 1 | Tail B | 5'**VIC**-GCCTTGCCAGCCCGC |
| TGGTTCATTGATCTCTTCTC | 1 | Tail C | 5'**NED**-CAGGACCAGGCTACCGTG |
| ATTTCTGGGAAGGGGCAGG | 1 | Tail D | 5'**PET**-CGGAGAGCCGAGAGGTG |
| TTCTTCTGTCCTGGCATTGC | 2 | Tail A | 5'**FAM**-GCCTCCCTCGCGCCA |
| ACTTCTGTGTCTCTAGCGCC | 2 | Tail B | 5'**VIC**-GCCTTGCCAGCCCGC |
| AACATTGGGTTTCACCTC | 2 | Tail C | 5'**NED**-CAGGACCAGGCTACCGTG |
| TTGGAGTCTTATGTCACCACC | 2 | Tail C | 5'**NED**-CAGGACCAGGCTACCGTG |
| AGAAGAGGTAGGAGAGGAAGTCA | 2 | Tail D | 5'**PET**-CGGAGAGCCGAGAGGTG |
| CTCATCCATGCTTCTTCTAC | 3 | Tail A | 5'**FAM**-GCCTCCCTCGCGCCA |
| TGGAGGTTATCTCATGCCAC | 3 | Tail A | 5'**FAM**-GCCTCCCTCGCGCCA |
| GAGGGTTTATTGTGAATGAG | 3 | Tail B | 5'**VIC**-GCCTTGCCAGCCCGC |
| CTTCTAGAGGGAGACTAGGAG | 3 | Tail B | 5'**VIC**-GCCTTGCCAGCCCGC |
| AGTGAAGACAGAAGGATCAC | 3 | Tail C | 5'**NED**-CAGGACCAGGCTACCGTG |
| AGCAATATCCACGATATGTGAAGG | 3 | Tail D | 5'**PET**-CGGAGAGCCGAGAGGTG |
| GGATTGGCATGGATGTTACC | 3 | Tail D | 5'**PET**-CGGAGAGCCGAGAGGTG |
| CTCTGTATCCACCTCATTCC | 4 | Tail A | 5'**FAM**-GCCTCCCTCGCGCCA |
| ATCATCAGAGTTTCCAGTTC | 4 | Tail A | 5'**FAM**-GCCTCCCTCGCGCCA |
| GGATGCTACCGAATAGATTG | 4 | Tail B | 5'**VIC**-GCCTTGCCAGCCCGC |
| CCTGACTCACACATCACCAG | 4 | Tail C | 5'**NED**-CAGGACCAGGCTACCGTG |
| GGGTCCAGTTGAGATATCAC | 4 | Tail D | 5'**PET**-CGGAGAGCCGAGAGGTG |
| AATGAACAGGAAGGAAGAC | 4 | Tail D | 5'**PET**-CGGAGAGCCGAGAGGTG |
| | | | **fluorescent tags in bold |

11-23

sequences

| Bin sizes for MSA |
|---|
| 211-223 |
| 184-200 |
| 232-252 |
| 217-263 |
| 224-242 |
| 234-272 |
| 218-240 |
| 135-160 |
| 135-160 |
| 118-150 |
| 145-186 |
| 98-122 |
| 165-180 |
| 220-242 |
| 250-268 |
| 148-175 |
| 198-228 |
| 202-218 |
| 250-269 |
| 190-214 |
| 139-159 |
| 172-218 |
| 238-247 |

**Supplementary Table S4**. List of 12 minerals and metals that were analyzed in rhino horn samples from Group 2 Horns ranked in order from highest to lowest mean concentration. Only samples collected 1-2 cm deep in the horn were included (n=47 samples from 16 horns, 10 rhinos). Concentrations of mercury and cadmium fell below detectable levels.

| **Rank** | **Mineral** | **Mean (ug/gm)** | **Range (ug/gm)** |
|---|---|---|---|
| 1 | Zinc | 99.11 | 39.21 – 181.63 |
| 2 | Iron | 6.529 | 3.24 – 26.03 |
| 3 | Copper | 2.294 | .828 – 4.315 |
| 4 | Aluminum | 3.367 | .200 – 11.437 |
| 5 | Selenium | .5309 | .3849 - .7873 |
| 6 | Barium | .2612 | .250 - .4080 |
| 7 | Chromium | .2508 | .086 - .708 |
| 8 | Arsenic | .1119 | .015 - .220 |
| 9 | Lead | .04147 | .025 - .3239 |
| 10 | Cobalt | .03415 | .002 - .3416 |
| 11 | Mercury | <0.500 | NA |
| 12 | Cadmium | <0.100 | NA |
